# Supplementary figures and images for: Defining the sediment prokaryotic communities of the Indian River Lagoon, FL, USA, an Estuary of National Significance
Source: PLoS One. 2020 Oct 26;15(10):e0236305. doi: 10.1371/journal.pone.0236305 (PMC7588086; doi:10.1371/journal.pone.0236305)

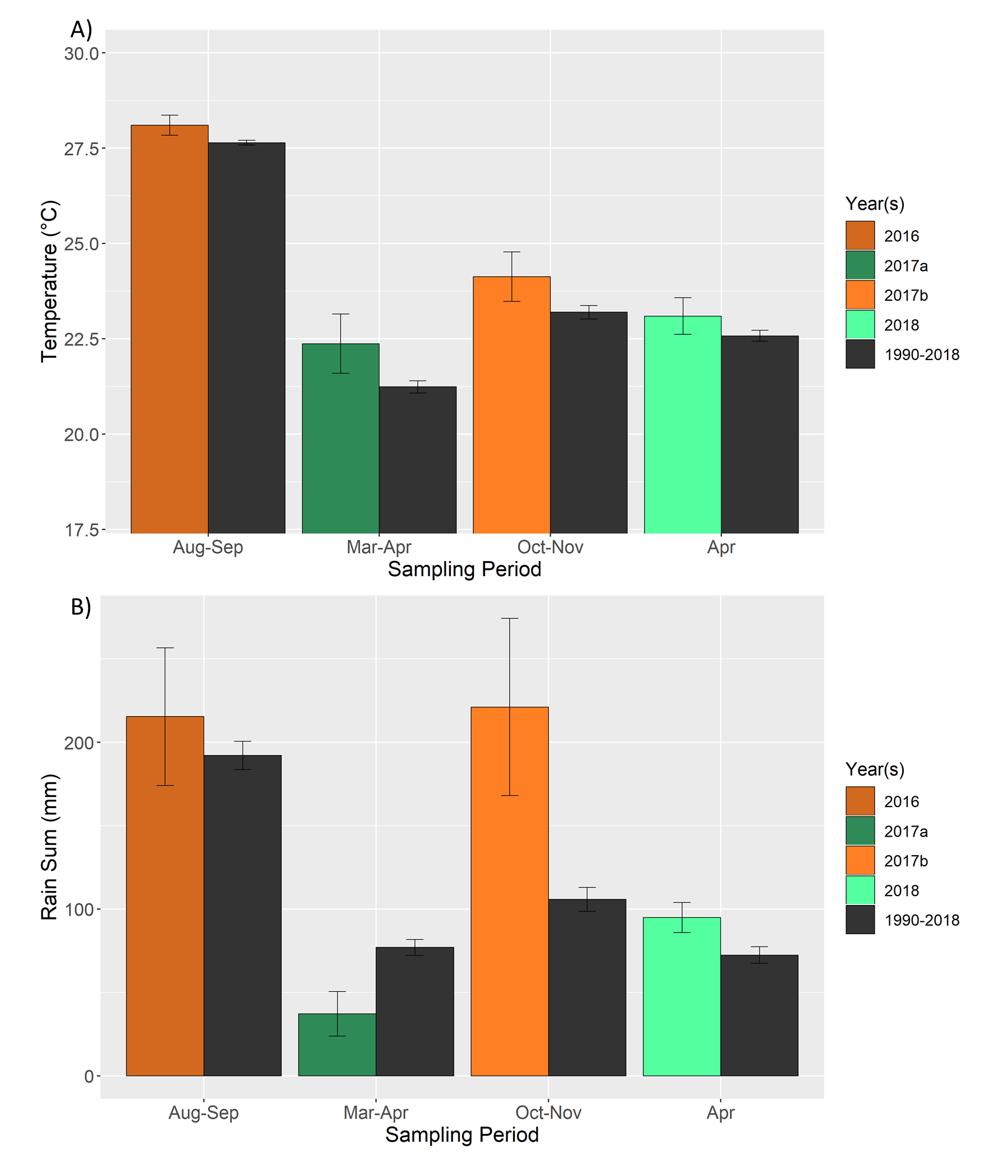

Supplement: S1 Fig — National Weather Service NOWData showing the four sampling periods (Aug-Sep 2016 (dark orange), Mar-Apr 2017a (dark green), Oct-Nov 2017b (light orange), Apr 2018 (light green)) as well as the historical temperature (A) and rain sum (B) during those months for the years 1990–2018 (dark gray). Bars denote standard error. (TIF) [file pone.0236305.s001.tif]

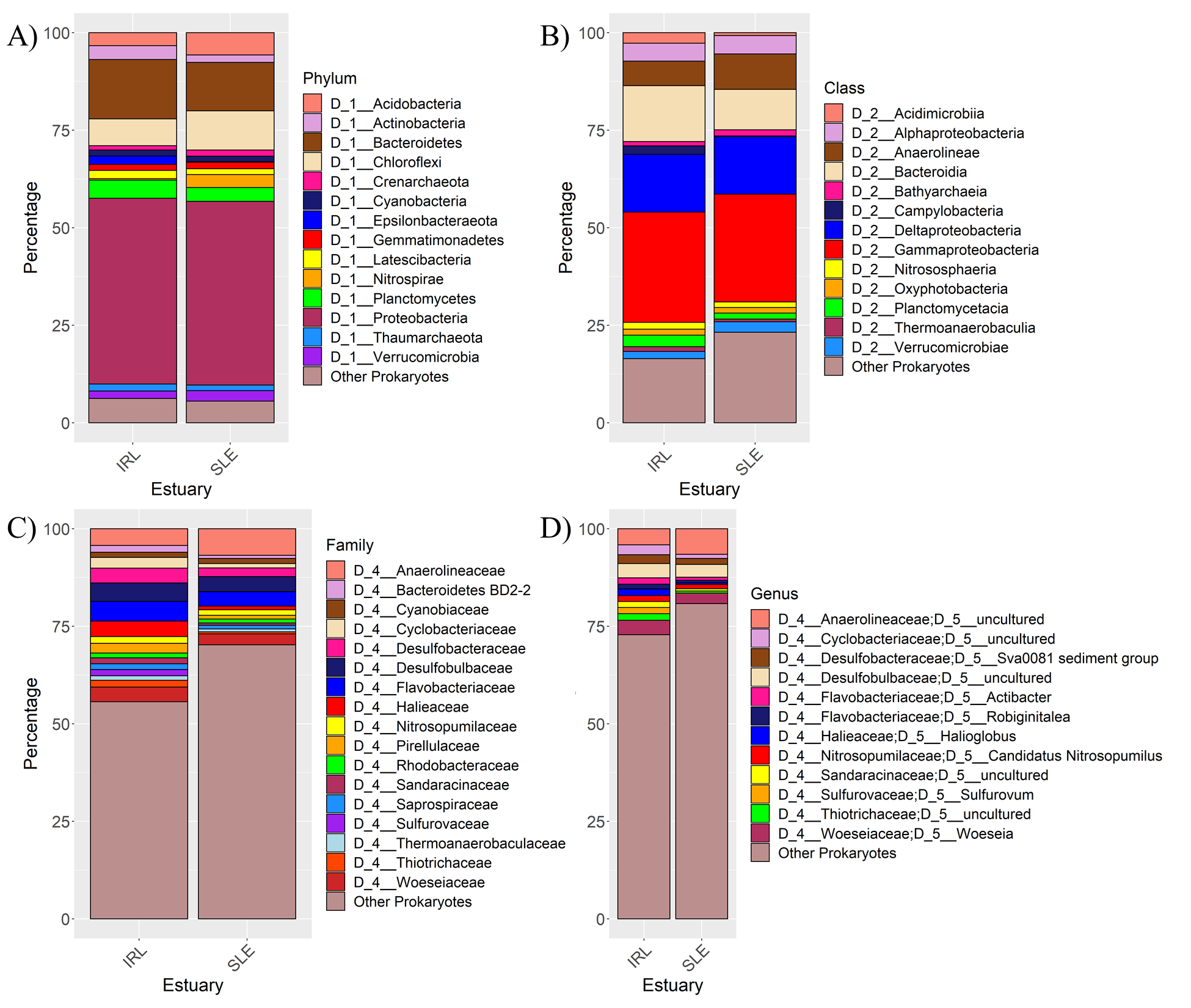

Supplement: S2 Fig — Stacked bar graphs showing the phyla (A), classes (B), orders (C), families (D), and genera (E) that have a mean of greater than 1% across all samples. These graphs show the differences between the two main basins of the study, (Indian River Lagoon (IRL) or St. Lucie Estuary (SLE)). (TIF) [file pone.0236305.s002.tif]

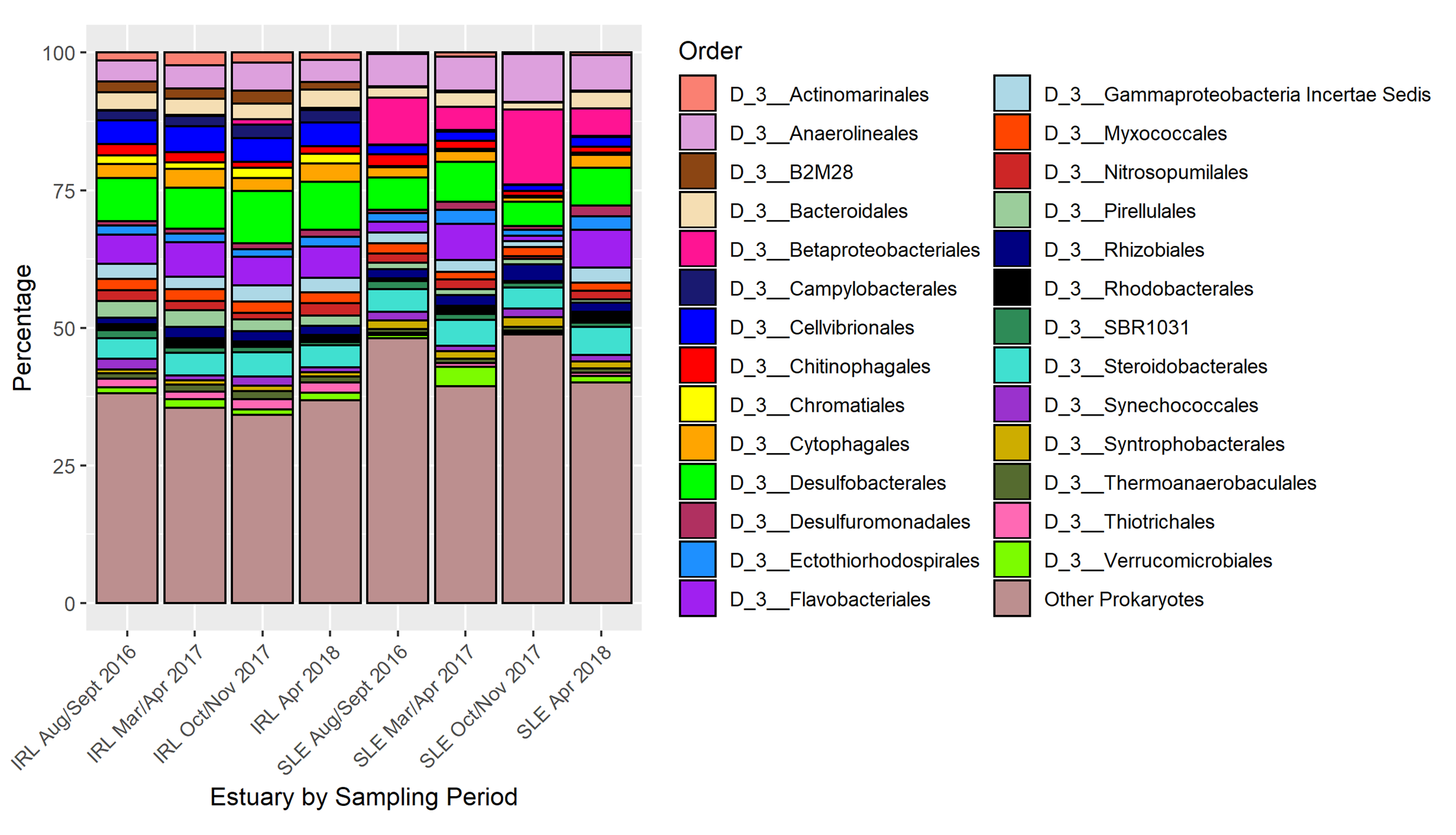

Supplement: S3 Fig — Stacked bar graph showing the orders with a mean greater than 1% across all samples grouped by estuary (Indian River Lagoon (IRL) or St. Lucie Estuary (SLE)) and sampling period (Aug/Sept 2016, Mar/Apr 2017, Oct/Nov 2017, and Apr 2018). (TIF) [file pone.0236305.s003.tif]

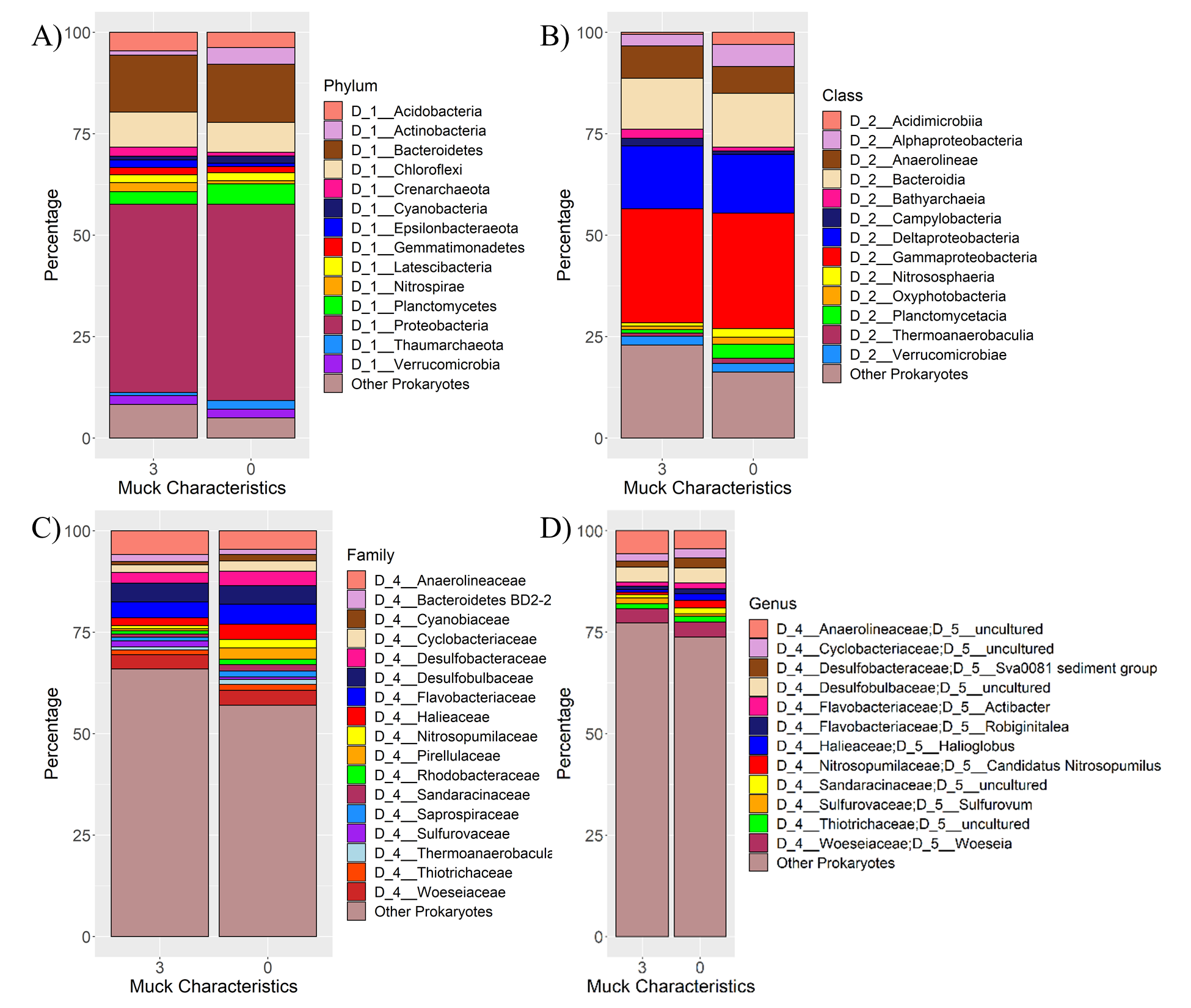

Supplement: S4 Fig — Stacked bar graphs showing the phyla (A), classes (B), orders (C), families (D), and genera (E) that have a mean of greater than 1% across all samples. These graphs show the differences between the samples with three and zero muck characteristics. (TIF) [file pone.0236305.s004.tif]

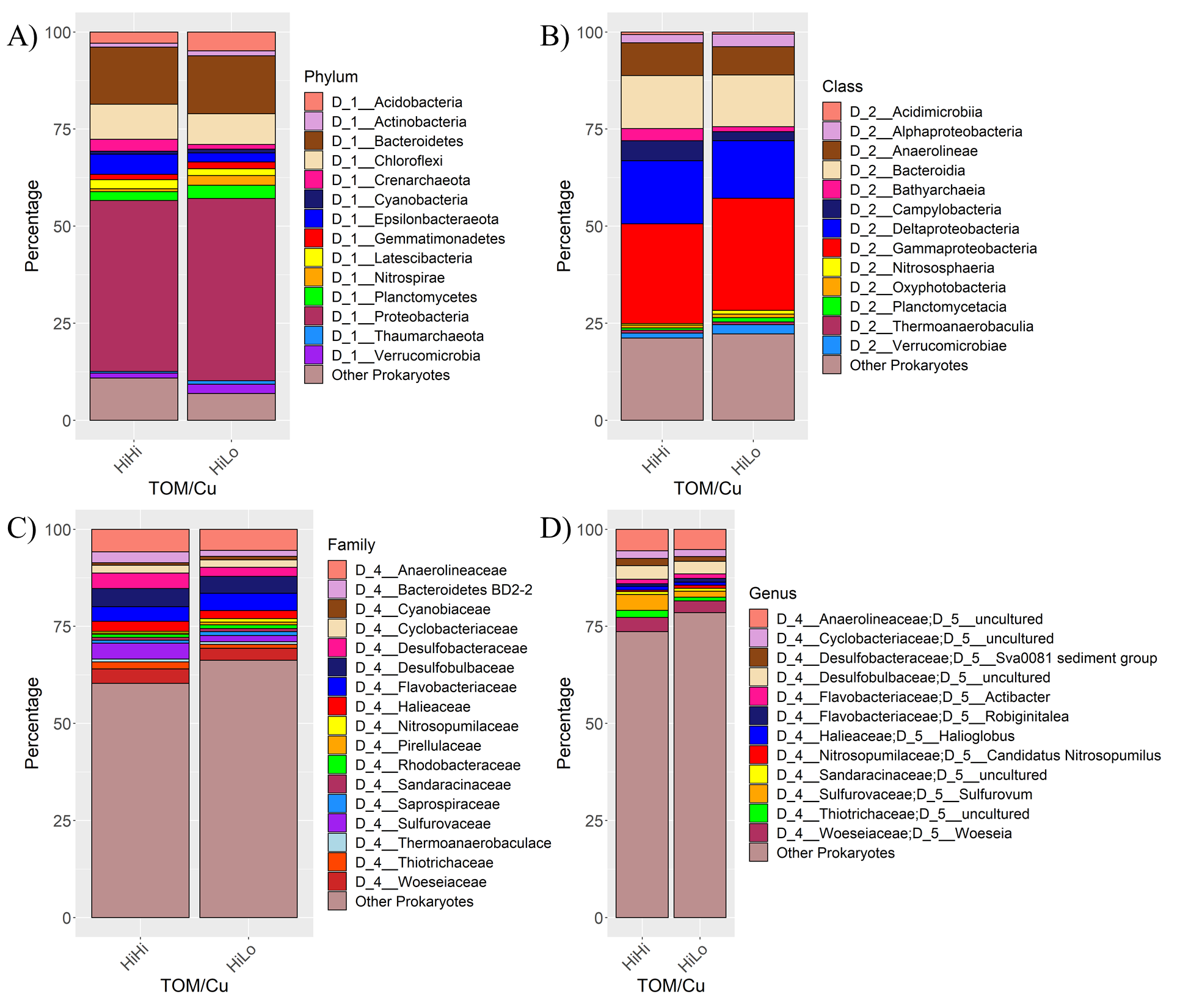

Supplement: S5 Fig — Other taxonomic levels by Total Organic Matter/Copper classification. Stacked bar graphs showing the phyla (A), classes (B), orders (C), families (D), and genera (E) that have a mean of greater than 1% across all samples. These graphs show the differences between the samples with high TOM and copper (HiHi) and high TOM and low copper (HiLo). (TIF) [file pone.0236305.s005.tif]
