# Supplementary material for: Defining the sediment prokaryotic communities of the Indian River Lagoon, FL, USA, an Estuary of National Significance
Source: PLoS One. 2020 Oct 26;15(10):e0236305. doi: 10.1371/journal.pone.0236305 (PMC7588086; doi:10.1371/journal.pone.0236305)
Supplement: S1 Table — aNWS stands for National Weather Service [30], bIRFWCD for Indian River Farms Water Control District, cUSGS for United States Geological Service [32], dSFWMD for South Florida Water Management District [31]. (DOCX) [file pone.0236305.s006.docx]

S1 Table: GPS coordinates

| Latitude | Longitude | Name | Description |
| --- | --- | --- | --- |
| 27°39'12.54" N | 80°22'30.54" W | Barber Bridge (BB) | Site |
| 27°28'28.44" N | 80°19'37.44" W | Fort Pierce (FP) | Site |
| 27°32'05.22" N | 80°21'20.16" W | Harbor Branch Channel (HB) | Site |
| 27°28'07.32" N | 80°19'41.04" W | Harbortown Marina (HT) | Site |
| 27°02'17.04" N | 80°06'38.40" W | Hobe Sound (HS) | Site |
| 27°14'06.48" N | 80°12'32.82" W | Jensen Beach (JB) | Site |
| 27°05'05.64" N | 80°07'49.26" W | Jupiter Narrows (JN) | Site |
| 27°32'05.10" N | 80°20'36.36" W | Linkport (LP) | Site |
| 27°09'05.10" N | 80°11'42.42" W | Manatee Pocket (MP) | Site |
| 28°05'02.28" N | 80°35'43.86" W | Melbourne Causeway (MC) | Site |
| 28°21'30.84" N | 80°38'47.94" W | Merritt Island Causeway (MI) | Site |
| 27°12'24.72" N | 80°15'03.90" W | Middle Estuary (ME) | Site |
| 27°12'32.40" N | 80°16'10.80" W | North Fork (NF) | Site |
| 27°33'41.58" N | 80°20'43.62" W | Round Island (RI) | Site |
| 27°50'22.02" N | 80°28'17.10" W | Sebastian Inlet (SI) | Site |
| 27°11'19.02" N | 80°15'52.02" W | South Fork (SF) | Site |
| 27°06'51.18" N | 80°16'58.62" W | South Fork 2 (ST) | Site |
| 27°33'55.86" N | 80°20'57.78" W | Vero Beach (VB) | Site |
| 27°39'24.66" N | 80°22'13.02" W | Vero Beach City Marina (VM) | Site |
| 27°29'5316'' N | 80°22'34.68'' W | Fort Pierce Area | NWS^a^ Weather Station |
| 28°5'5892'' N | 80°38'8.16'' W | Melbourne Area | NWS Weather Station |
| 27°11'2256'' N | 80°14'15.36'' W | Stuart 4E | NWS Weather Station |
| 27°41'34" N | 80°25'13" W | IRFWCD^b^ North Canal | USGS^c^ Streamflow Station |
| 27°38'51" N | 80°24'21" W | IRFWCD Main Canal | USGS Streamflow Station |
| 27°36'15" N | 80°23'12.5" W | IRFWCD South Canal | USGS Streamflow Station |
| 28°07'36" N | 80°38'49" W | Eau Gallie River | USGS Streamflow Station |
| 28°04'45" N | 80°37'47" W | Crane Creek | USGS Streamflow Station |
| 28°01'01" N | 80°35'46" W | Turkey Creek | USGS Streamflow Station |
| 27°51'21" N | 80°31'28" W | North Prong St. Sebastian River | USGS Streamflow Station |
| 27°49'49" N | 80°32'04" W | Fellsmere Canal | USGS Streamflow Station |
| 27°46'09" N | 80°30'22" W | South Prong St. Sebastian River | USGS Streamflow Station |
| 27° 06'31'' N | 80°17'14'' W | C44 | SFWMD^d^ Streamflow Station |
| 27°15'41' 'N | 80°21'33'' W | C24 | SFWMD Streamflow Station |
| 27°28'07'' N | 80°20'17'' W | C25 (Taylor Creek) | SFWMD Streamflow Station |
| 27°12'06'' N | 80°17'57'' W | C23 | SFWMD Streamflow Station |
| 27°24'11'' N | 80°23'57'' W | Ten Mile Creek | SFWMD Streamflow Station |

^a^NWS stands for National Weather Service (1), ^b^IRFWCD for Indian River Farms Water Control District, ^c^USGS for United States Geological Service (2), ^d^SFWMD for South Florida Water Management District (3).
