# Supplementary material for: Defining the sediment prokaryotic communities of the Indian River Lagoon, FL, USA, an Estuary of National Significance
Source: PLoS One. 2020 Oct 26;15(10):e0236305. doi: 10.1371/journal.pone.0236305 (PMC7588086; doi:10.1371/journal.pone.0236305)
Supplement: S2 Table — Sediment temperature was determined using a thermometer, water content by weight loss during oven drying, total organic matter by weight loss in a muffle furnace, grain size fractions (gravel, sand, and silt/clay) were determined using wet sieving, and copper (Cu) and iron (Fe) were measured using an atomic adsorption spectrometer. See manuscript for details. (DOCX) [file pone.0236305.s007.docx]

S2 Table: Measured environmental variables per sample

| Sample Name | Sediment Temperature (°C) | Water Content (%) | Total Organic Matter (%) | Porewater Salinity (ppt) | Gravel Fraction (%) | Sand Fraction (%) | Silt/Clay Fraction (%) | Cu (µg/g) | Fe  (µg/g) |
| --- | --- | --- | --- | --- | --- | --- | --- | --- | --- |
| BB1D18S | 25.1 | 35.490 | 4.331 | 41 | 0.383 | 87.816 | 11.802 | 7.550 | 2062.316 |
| BB1W17S | 25 | 33.606 | 3.081 | 24 | 1.270 | 93.715 | 5.015 | 6.429 | 1423.694 |
| BB2D18S | 25.1 | 41.788 | 4.891 | 42 | 0.025 | 84.557 | 15.418 | 9.440 | 2493.996 |
| BB2W17S | 25 | 28.690 | 1.590 | 15 | 0.458 | 95.525 | 4.017 | 3.160 | 711.420 |
| BB3D18S | 25.1 | 37.869 | 4.571 | 42 | 0.780 | 85.354 | 13.866 | 7.344 | 2124.940 |
| BB3W17S | 25 | 29.097 | 1.680 | 23 | 0.072 | 95.963 | 3.965 | 3.794 | 804.697 |
| FP1D17S | 20 | 26.347 | 2.680 | 42 | 5.621 | 90.076 | 4.303 | 1.662 | 1115.279 |
| FP1D18S | 23.2 | 23.771 | 1.959 | 40 | 2.632 | 94.613 | 2.755 | 1.264 | 940.500 |
| FP1W16S | 30.5 | 26.699 | 2.700 | 40 | 12.627 | 84.905 | 2.469 | 1.880 | 1050.497 |
| FP1W17S-3 | 21.5 | 29.245 | 2.361 | 34 | 4.270 | 90.658 | 5.072 | 2.413 | 1279.792 |
| FP2D17S | 20 | 28.423 | 2.609 | 40 | 7.685 | 88.701 | 3.614 | 1.385 | 1056.474 |
| FP2D18S | 23.2 | 25.539 | 1.879 | 41 | 2.831 | 94.859 | 2.310 | 1.264 | 829.042 |
| FP2W16S | 30.5 | 28.162 | 3.521 | 41 | 7.322 | 89.147 | 3.532 | 1.879 | 943.642 |
| FP2W17S-3 | 21.5 | 32.690 | 2.841 | 33 | 4.030 | 89.967 | 6.003 | 2.853 | 1390.322 |
| FP3D17S | 20 | 28.531 | 2.320 | 40 | 15.381 | 81.781 | 2.839 | 1.662 | 997.881 |
| FP3D18S | 23.2 | 25.294 | 1.570 | 41 | 4.530 | 93.169 | 2.301 | 1.264 | 730.198 |
| FP3W16S | 30.5 | 27.494 | 2.779 | 40 | 15.604 | 82.069 | 2.327 | 1.879 | 837.018 |
| FP3W17S-3 | 21.5 | 31.522 | 2.421 | 34 | 2.923 | 91.121 | 5.956 | 2.851 | 1389.488 |
| HB1D17S | 23.7 | 75.713 | 15.355 | 46 | 3.937 | 49.187 | 46.876 | 109.689 | 6712.425 |
| HB1D18S | 25.3 | 76.422 | 13.774 | 47 | 0.296 | 42.804 | 56.900 | 85.126 | 5074.257 |
| HB1W16S | 30 | 84.609 | 19.334 | 37 | 0 | 34.073 | 65.927 | 106.327 | 6638.981 |
| HB1W17S-3 | 23.6 | 77.171 | 14.026 | 16 | 5.455 | 63.771 | 30.775 | 109.664 | 5728.591 |
| HB2D17S | 23.7 | 79.647 | 18.275 | 46 | 0 | 36.375 | 63.625 | 125.213 | 7962.044 |
| HB2D18S | 25.3 | 75.282 | 13.436 | 46 | 1.001 | 39.631 | 59.368 | 76.713 | 4858.645 |
| HB2W16S | 30 | 82.955 | 19.644 | 36 | 0 | 16.143 | 83.857 | 111.708 | 6330.824 |
| HB2W17S-3 | 23.6 | 76.543 | 9.151 | 7 | 8.597 | 57.898 | 33.505 | 114.997 | 4756.365 |
| Sample Name | Sediment Temperature (°C) | Water Content (%) | Total Organic Matter (%) | Porewater Salinity (ppt) | Gravel Fraction (%) | Sand Fraction (%) | Silt/Clay Fraction (%) | Cu (ppm) | Fe  (ppm) |
| HB3D17S | 23.7 | 74.898 | 15.565 | 47 | 0.333 | 48.939 | 50.728 | 123.033 | 5778.118 |
| HB3D18S | 25.3 | 64.697 | 9.071 | 40 | 4.294 | 62.301 | 33.405 | 84.258 | 3216.857 |
| HB3W16S | 30 | 82.894 | 18.498 | 36 | 0.697 | 30.203 | 69.100 | 104.189 | 6485.235 |
| HB3W17S-3 | 23.6 | 80.268 | 17.237 | 30 | 0 | 38.340 | 61.660 | 107.471 | 5728.591 |
| HS1D17S | 25 | 21.734 | 0.970 | 42 | 0.096 | 96.642 | 3.263 | 0.554 | 303.785 |
| HS1D18S | 23.4 | 30.519 | 2.790 | 43 | 0.207 | 92.788 | 7.005 | 3.582 | 1237.624 |
| HS1W16S | 31.7 | 29.005 | 2.471 | 42 | 0.064 | 93.864 | 6.073 | 2.954 | 1081.055 |
| HS1W17S | 25.8 | 27.985 | 2.230 | 27 | 0.106 | 92.795 | 7.099 | 2.762 | 845.182 |
| HS2D17S | 25 | 25.591 | 2.050 | 43 | 0.307 | 93.865 | 5.827 | 1.939 | 888.891 |
| HS2D18S | 23.4 | 30.361 | 3.061 | 45 | 0.252 | 91.658 | 8.090 | 3.792 | 1435.213 |
| HS2W16S | 31.7 | 21.396 | 0.910 | 41 | 0.374 | 96.362 | 3.264 | 0.806 | 243.545 |
| HS2W17S | 25.8 | 25.990 | 1.480 | 26 | 0.300 | 95.681 | 4.018 | 1.913 | 509.748 |
| HS3D17S | 25 | 23.352 | 2.030 | 43 | 0.420 | 95.063 | 4.517 | 1.662 | 874.000 |
| HS3D18S | 23.4 | 28.709 | 2.681 | 42 | 0.115 | 93.225 | 6.659 | 3.371 | 1107.673 |
| HS3W16S | 31.7 | 26.347 | 1.760 | 41.5 | 0.249 | 92.420 | 7.330 | 2.148 | 837.269 |
| HS3W17S | 25.8 | 26.622 | 1.560 | 27 | 0.217 | 95.356 | 4.427 | 1.700 | 472.544 |
| HT1D18S | 24.1 | 76.377 | 18.652 | 47 | 0 | 41.897 | 58.103 | 63.776 | 3263.748 |
| HT1W17S-3 | 20.7 | 82.888 | 19.004 | 19 | 0 | 62.391 | 37.609 | 65.522 | 5433.560 |
| HT2D18S | 24.1 | 68.394 | 13.826 | 47 | 0 | 63.274 | 36.726 | 47.822 | 3817.206 |
| HT2W17S-3 | 20.7 | 82.626 | 27.318 | 29 | 0 | 38.150 | 61.850 | 78.054 | 6747.808 |
| HT3D18S | 24.1 | 65.157 | 11.230 | 48 | 0 | 82.535 | 17.465 | 46.149 | 4802.771 |
| HT3W17S-3 | 20.7 | 75.956 | 16.235 | 20 | 0 | 58.873 | 41.127 | 73.712 | 5246.196 |
| JB1D17S | 24.5 | 42.354 | 6.999 | 42 | 1.044 | 77.057 | 21.900 | 3.602 | 2767.628 |
| JB1D18S | 26.1 | 33.223 | 3.771 | 45 | 1.042 | 85.101 | 13.857 | 2.736 | 1561.048 |
| JB1W16S | 29.4 | 38.824 | 5.271 | 33 | 0.421 | 81.136 | 18.444 | 3.222 | 2285.168 |
| JB1W17S | 23.8 | 29.673 | 2.390 | 21 | 0.221 | 96.451 | 3.328 | 1.913 | 982.393 |
| JB2D17S | 24.5 | 40.513 | 5.961 | 43 | 0.096 | 78.957 | 20.947 | 3.878 | 2518.601 |
| JB2D18S | 26.1 | 32.124 | 3.201 | 43 | 0 | 92.420 | 7.580 | 2.314 | 1387.321 |
| Sample Name | Sediment Temperature (°C) | Water Content (%) | Total Organic Matter (%) | Porewater Salinity (ppt) | Gravel Fraction (%) | Sand Fraction (%) | Silt/Clay Fraction (%) | Cu (ppm) | Fe  (ppm) |
| JB2W16S | 29.4 | 42.835 | 6.389 | 31 | 0.179 | 73.821 | 26.000 | 4.028 | 2448.640 |
| JB2W17S | 23.8 | 42.736 | 4.900 | 25 | 0.681 | 85.661 | 13.658 | 3.613 | 1959.405 |
| JB3D17S | 24.5 | 46.567 | 8.098 | 42 | 0.738 | 78.289 | 20.973 | 4.709 | 3202.665 |
| JB3D18S | 26.1 | 30.954 | 3.311 | 43 | 7.091 | 83.914 | 8.995 | 2.315 | 1350.701 |
| JB3W16S | 29.4 | 41.898 | 5.502 | 33 | 0.655 | 79.130 | 20.214 | 3.492 | 2332.737 |
| JB3W17S | 23.8 | 33.333 | 3.149 | 22 | 0.167 | 87.936 | 11.897 | 2.549 | 1441.780 |
| JN1D17S | 25 | 46.376 | 5.689 | 43 | 0 | 89.247 | 10.753 | 7.760 | 1555.314 |
| JN1D18S | 24 | 30.896 | 2.440 | 41 | 0.995 | 94.580 | 4.424 | 2.947 | 966.556 |
| JN1W16S | 31.5 | 28.261 | 2.570 | 39 | 0.114 | 96.793 | 3.093 | 2.416 | 708.100 |
| JN1W17S | 27 | 29.003 | 1.590 | 25 | 0.318 | 96.945 | 2.737 | 2.338 | 609.332 |
| JN2D17S | 25 | 32.868 | 3.089 | 42 | 0.069 | 94.646 | 5.285 | 3.880 | 902.082 |
| JN2D18S | 24 | 32.658 | 2.850 | 40 | 1.026 | 93.147 | 5.827 | 3.578 | 1115.369 |
| JN2W16S | 31.5 | 33.728 | 3.420 | 38 | 0.230 | 93.716 | 6.055 | 4.028 | 1069.790 |
| JN2W17S | 27 | 28.629 | 1.869 | 20 | 0.884 | 95.753 | 3.363 | 2.763 | 690.093 |
| JN3D17S | 25 | 41.310 | 4.350 | 43 | 0 | 90.367 | 9.633 | 6.093 | 1243.505 |
| JN3D18S | 24 | 29.585 | 2.541 | 40 | 1.343 | 93.044 | 5.612 | 2.735 | 891.849 |
| JN3W16S | 31.5 | 29.555 | 2.820 | 39 | 0.139 | 95.041 | 4.820 | 2.686 | 693.311 |
| JN3W17S | 27 | 26.418 | 1.220 | 25 | 0.388 | 95.006 | 4.606 | 1.912 | 484.785 |
| LP1D17S | 21 | 27.088 | 2.030 | 37 | 0.268 | 95.822 | 3.910 | 2.493 | 645.752 |
| LP1D18S | 25 | 38.380 | 3.861 | 47 | 0.328 | 91.142 | 8.530 | 5.262 | 1065.797 |
| LP1W16S | 32 | 25.108 | 1.900 | 39 | 0.159 | 98.667 | 1.174 | 1.343 | 391.793 |
| LP1W17S-3 | 20.9 | 35.904 | 3.520 | 25 | 0.156 | 92.669 | 7.175 | 5.173 | 1236.603 |
| LP2D17S | 21 | 32.930 | 2.791 | 38 | 0.106 | 95.245 | 4.649 | 3.602 | 1027.539 |
| LP2D18S | 25 | 62.505 | 10.507 | 46 | 0.128 | 62.477 | 37.395 | 17.041 | 3303.369 |
| LP2W16S | 32 | 25.433 | 1.990 | 40 | 0.419 | 97.001 | 2.580 | 1.880 | 497.375 |
| LP2W17S-3 | 20.9 | 38.179 | 3.840 | 25 | 0.052 | 89.912 | 10.036 | 5.173 | 1249.094 |
| LP3D17S | 21 | 36.168 | 4.370 | 39 | 0.088 | 90.548 | 9.364 | 4.710 | 1394.517 |
| LP3D18S | 25 | 66.854 | 12.579 | 46 | 0 | 52.377 | 47.623 | 21.670 | 3350.225 |
| Sample Name | Sediment Temperature (°C) | Water Content (%) | Total Organic Matter (%) | Porewater Salinity (ppt) | Gravel Fraction (%) | Sand Fraction (%) | Silt/Clay Fraction (%) | Cu (ppm) | Fe  (ppm) |
| LP3W16S | 32 | 27.797 | 2.729 | 40 | 0.260 | 96.311 | 3.429 | 2.148 | 602.699 |
| LP3W17S-3 | 20.9 | 37.207 | 3.229 | 25 | 0.054 | 94.664 | 5.282 | 5.173 | 1186.640 |
| MC1D17S | 22.9 | 85.231 | 21.404 | 36 | 0 | 1.916 | 98.084 | 46.187 | 8399.923 |
| MC1D18S | 25.3 | 47.374 | 4.700 | 39 | 0.365 | 81.678 | 17.957 | 8.418 | 1858.576 |
| MC1W16S | 30 | 75.046 | 15.365 | 38 | 4.531 | 34.868 | 60.601 | 26.850 | 4741.810 |
| MC1W17S | 22.5 | 75.961 | 13.830 | 27 | 0.086 | 72.568 | 27.347 | 25.490 | 6027.530 |
| MC2D17S | 22.9 | 85.959 | 21.526 | 35 | 1.818 | 16.304 | 81.878 | 45.099 | 8397.404 |
| MC2D18S | 25.3 | 63.151 | 7.309 | 40 | 0.330 | 83.216 | 16.454 | 13.887 | 3530.589 |
| MC2W16S | 30 | 81.718 | 19.538 | 38 | 4.901 | 7.094 | 88.005 | 33.039 | 6299.021 |
| MC2W17S | 22.5 | 41.630 | 2.780 | 27 | 0.076 | 93.010 | 6.914 | 4.462 | 1056.583 |
| MC3D17S | 22.9 | 69.107 | 9.447 | 35 | 2.574 | 18.367 | 79.059 | 22.561 | 3830.143 |
| MC3D18S | 25.3 | 76.603 | 16.402 | 37 | 0 | 38.749 | 61.251 | 30.942 | 7621.688 |
| MC3W16S | 30 | 77.974 | 18.138 | 39 | 10.671 | 33.601 | 55.727 | 31.152 | 5442.510 |
| MC3W17S | 22.5 | 80.274 | 17.200 | 27 | 0 | 40.320 | 59.680 | 28.897 | 6713.078 |
| ME1D17S | 24.5 | 78.573 | 25.385 | 36 | 0.098 | 9.567 | 90.334 | 27.428 | 6816.157 |
| ME1D18S | 25.9 | 80.627 | 25.957 | 36 | 3.480 | 13.374 | 83.146 | 26.940 | 6319.792 |
| ME1W16S | 29.8 | 81.647 | 26.683 | 18 | 25.211 | 9.442 | 65.347 | 26.861 | 6487.181 |
| ME1W17S | 22.8 | 88.567 | 29.731 | 5 | 0 | 5.500 | 94.500 | 22.094 | 5965.987 |
| ME2D17S | 24.5 | 79.308 | 25.400 | 37 | 0.551 | 20.357 | 79.093 | 25.770 | 6507.634 |
| ME2D18S | 25.9 | 81.234 | 26.453 | 37 | 0.298 | 12.245 | 87.458 | 25.254 | 3469.343 |
| ME2W16S | 29.8 | 81.920 | 26.342 | 17 | 40.385 | 8.559 | 51.056 | 26.055 | 6564.409 |
| ME2W17S | 22.8 | 89.271 | 30.184 | 5 | 0 | 4.747 | 95.253 | 21.250 | 5905.613 |
| ME3D17S | 24.5 | 78.198 | 25.138 | 35 | 0 | 8.645 | 91.355 | 26.868 | 6892.235 |
| ME3D18S | 25.9 | 75.341 | 22.857 | 37 | 1.890 | 24.617 | 73.493 | 24.417 | 5019.160 |
| ME3W16S | 29.8 | 83.447 | 27.285 | 16 | 33.927 | 25.677 | 40.395 | 26.047 | 7025.671 |
| ME3W17S | 22.8 | 88.838 | 30.401 | 5 | 0 | 7.756 | 92.244 | 22.098 | 5967.181 |
| MI1D17S | 25.5 | 24.389 | 1.550 | 33 | 1.073 | 92.998 | 5.929 | 1.662 | 416.479 |
| MI1D18S | 23.9 | 23.982 | 1.490 | 29 | 4.610 | 94.248 | 1.142 | 1.262 | 247.761 |
| Sample Name | Sediment Temperature (°C) | Water Content (%) | Total Organic Matter (%) | Porewater Salinity (ppt) | Gravel Fraction (%) | Sand Fraction (%) | Silt/Clay Fraction (%) | Cu (ppm) | Fe  (ppm) |
| MI1W16S | 29 | 28.019 | 2.431 | 33 | 4.424 | 94.508 | 1.068 | 2.148 | 271.187 |
| MI1W17S | 23.6 | 20.515 | 1.110 | 25 | 5.594 | 93.532 | 0.874 | 1.275 | 159.270 |
| MI2D17S | 25.5 | 26.588 | 2.189 | 34 | 1.039 | 94.912 | 4.050 | 1.386 | 323.578 |
| MI2D18S | 23.9 | 23.189 | 1.320 | 29 | 1.885 | 96.772 | 1.343 | 1.052 | 223.051 |
| MI2W16S | 29 | 31.063 | 2.640 | 33 | 3.224 | 93.842 | 2.934 | 1.880 | 361.691 |
| MI2W17S | 23.6 | 22.363 | 1.190 | 26 | 12.959 | 86.010 | 1.030 | 1.487 | 135.661 |
| MI3D17S | 25.5 | 29.637 | 2.200 | 34 | 0.872 | 96.116 | 3.013 | 0.831 | 191.205 |
| MI3D18S | 23.9 | 24.219 | 1.330 | 30 | 2.123 | 96.310 | 1.567 | 1.052 | 159.349 |
| MI3W16S | 29 | 24.619 | 1.430 | 34 | 2.362 | 94.054 | 3.584 | 1.342 | 210.902 |
| MI3W17S | 23.6 | 19.553 | 1.550 | 25 | 10.593 | 87.184 | 2.223 | 1.488 | 177.037 |
| MP1D17S | 25.5 | 58.972 | 9.161 | 45 | 0.912 | 56.807 | 42.281 | 59.094 | 2224.176 |
| MP1D18S | 26.6 | 73.646 | 17.063 | 45 | 0 | 44.434 | 55.566 | 109.198 | 4081.002 |
| MP1W16S | 29.2 | 77.185 | 19.994 | 40 | 0 | 24.895 | 75.105 | 109.582 | 4169.913 |
| MP1W17S | 25.1 | 78.152 | 20.584 | 37 | 0 | 29.892 | 70.108 | 114.097 | 4699.960 |
| MP2D17S | 25.5 | 79.004 | 20.296 | 45 | 0 | 24.992 | 75.008 | 136.426 | 4694.544 |
| MP2D18S | 26.6 | 76.321 | 18.194 | 45 | 0.419 | 34.898 | 64.684 | 131.917 | 4886.301 |
| MP2W16S | 29.2 | 80.148 | 22.113 | 41 | 0 | 18.596 | 81.404 | 142.900 | 4633.700 |
| MP2W17S | 25.1 | 79.471 | 22.687 | 37 | 0.124 | 15.375 | 84.501 | 125.929 | 5627.584 |
| MP3D17S | 25.5 | 64.513 | 11.141 | 45 | 0.605 | 61.694 | 37.701 | 78.434 | 2965.568 |
| MP3D18S | 26.6 | 72.923 | 18.120 | 45 | 0 | 34.488 | 65.512 | 125.157 | 4484.040 |
| MP3W16S | 29.2 | 81.178 | 22.184 | 41 | 0 | 15.165 | 84.835 | 141.741 | 4630.921 |
| MP3W17S | 25.1 | 76.245 | 19.456 | 37 | 0.199 | 31.684 | 68.117 | 118.335 | 4514.887 |
| NF1D17S | 24.5 | 25.854 | 5.231 | 34 | 5.112 | 87.118 | 7.770 | 6.713 | 1287.978 |
| NF1D18S | 25.3 | 33.648 | 3.480 | 35 | 4.771 | 84.321 | 10.909 | 5.462 | 766.964 |
| NF1W16S | 29.7 | 56.346 | 7.648 | 9 | 0 | 80.172 | 19.828 | 9.133 | 2280.242 |
| NF1W17S | 23.2 | 50.325 | 6.289 | 7 | 5.517 | 81.170 | 13.313 | 9.301 | 1460.045 |
| NF2D17S | 24.5 | 39.967 | 5.060 | 35 | 3.760 | 86.955 | 9.285 | 6.444 | 1241.142 |
| NF2D18S | 25.3 | 42.569 | 4.820 | 35 | 4.921 | 85.490 | 9.589 | 6.719 | 1088.050 |
| Sample Name | Sediment Temperature (°C) | Water Content (%) | Total Organic Matter (%) | Porewater Salinity (ppt) | Gravel Fraction (%) | Sand Fraction (%) | Silt/Clay Fraction (%) | Cu (ppm) | Fe  (ppm) |
| NF2W16S | 29.7 | 55.199 | 7.059 | 8 | 0 | 86.702 | 13.298 | 8.863 | 2186.315 |
| NF2W17S | 23.2 | 44.835 | 5.159 | 6 | 6.998 | 83.099 | 9.902 | 7.186 | 1088.738 |
| NF3D17S | 24.5 | 35.382 | 3.811 | 34 | 5.223 | 87.111 | 7.666 | 5.371 | 890.053 |
| NF3D18S | 25.3 | 38.517 | 3.219 | 35 | 8.028 | 85.873 | 6.098 | 5.460 | 766.810 |
| NF3W16S | 29.7 | 54.054 | 7.159 | 9 | 0 | 78.522 | 21.478 | 8.058 | 2217.770 |
| NF3W17S | 23.2 | 45.613 | 4.751 | 7 | 6.380 | 82.500 | 11.120 | 6.552 | 1014.506 |
| RI1D18S | 24.8 | 29.081 | 1.530 | 43 | 0.170 | 95.977 | 3.854 | 1.889 | 776.143 |
| RI1W17S | 24.7 | 32.106 | 1.850 | 22 | 0.049 | 95.377 | 4.574 | 2.845 | 841.837 |
| RI2D18S | 24.8 | 25.724 | 1.260 | 45 | 0.563 | 97.122 | 2.315 | 1.259 | 492.789 |
| RI2W17S | 24.7 | 33.253 | 2.010 | 22 | 0.060 | 94.083 | 5.857 | 3.160 | 866.077 |
| RI3D18S | 24.8 | 29.951 | 1.730 | 45 | 0.214 | 95.183 | 4.604 | 2.098 | 825.174 |
| RI3W17S | 24.7 | 43.251 | 3.170 | 23 | 0.078 | 87.595 | 12.327 | 5.478 | 1497.375 |
| SF1D17S | 24.5 | 82.147 | 26.998 | 34 | 0 | 12.269 | 87.731 | 29.010 | 4803.734 |
| SF1D18S | 25.6 | 79.838 | 27.488 | 35 | 0.330 | 14.448 | 85.221 | 32.763 | 5318.201 |
| SF1W16S | 30 | 85.747 | 29.543 | 10 | 0 | 14.107 | 85.893 | 35.456 | 5656.009 |
| SF1W17S | 22.9 | 81.798 | 26.290 | 6 | 0 | 5.913 | 94.087 | 23.669 | 5690.563 |
| SF2D17S | 24.5 | 83.510 | 26.825 | 34 | 0 | 8.849 | 91.151 | 29.004 | 4802.773 |
| SF2D18S | 25.6 | 84.121 | 27.185 | 35 | 0 | 9.686 | 90.314 | 34.450 | 5257.413 |
| SF2W16S | 30 | 86.338 | 29.521 | 9 | 0 | 7.559 | 92.441 | 30.081 | 5035.669 |
| SF2W17S | 22.9 | 77.899 | 28.723 | 5 | 0 | 12.840 | 87.160 | 22.822 | 5782.766 |
| SF3D17S | 24.5 | 76.880 | 28.128 | 35 | 0.392 | 7.246 | 92.362 | 31.677 | 5342.884 |
| SF3D18S | 25.6 | 83.350 | 28.529 | 35 | 0.394 | 6.779 | 92.827 | 35.272 | 5378.427 |
| SF3W16S | 30 | 85.114 | 29.071 | 9 | 0 | 6.463 | 93.537 | 35.707 | 5498.300 |
| SF3W17S | 22.9 | 83.214 | 26.658 | 6 | 60.046 | 6.529 | 33.425 | 23.672 | 5567.412 |
| SI1D17S | 22.7 | 24.597 | 1.960 | 41 | 0 | 97.619 | 2.381 | 0.805 | 677.900 |
| SI1D18S | 25.5 | 24.979 | 1.790 | 42 | 1.141 | 93.518 | 5.342 | 1.470 | 964.890 |
| SI1W16S | 29.5 | 29.849 | 2.770 | 40 | 1.614 | 92.108 | 6.278 | 1.342 | 928.329 |
| SI1W17S | 24.1 | 25.113 | 1.670 | 37 | 0.586 | 96.877 | 2.537 | 1.269 | 643.538 |
| Sample Name | Sediment Temperature (°C) | Water Content (%) | Total Organic Matter (%) | Porewater Salinity (ppt) | Gravel Fraction (%) | Sand Fraction (%) | Silt/Clay Fraction (%) | Cu (ppm) | Fe  (ppm) |
| SI2D17S | 22.7 | 25.403 | 1.830 | 42 | 0 | 97.588 | 2.412 | 1.074 | 753.223 |
| SI2D18S | 25.5 | 27.450 | 2.010 | 42 | 1.136 | 93.892 | 4.972 | 1.470 | 828.401 |
| SI2W16S | 29.5 | 26.416 | 1.830 | 40 | 0.704 | 95.873 | 3.423 | 1.074 | 608.862 |
| SI2W17S | 24.1 | 34.406 | 3.411 | 40 | 1.867 | 91.017 | 7.117 | 2.958 | 606.108 |
| SI3D17S | 22.7 | 24.331 | 1.420 | 42 | 0 | 97.568 | 2.432 | 0.806 | 452.114 |
| SI3D18S | 25.5 | 23.487 | 1.800 | 40 | 1.141 | 92.642 | 6.216 | 1.260 | 841.102 |
| SI3W16S | 29.5 | 25.174 | 1.540 | 40 | 0.530 | 96.069 | 3.401 | 0.805 | 487.041 |
| SI3W17S | 24.1 | 22.132 | 1.620 | 40 | 1.007 | 95.859 | 3.134 | 1.057 | 1484.792 |
| ST1D17S | 24 | 57.271 | 8.330 | 24 | 0.559 | 78.348 | 21.093 | 12.087 | 1686.755 |
| ST1D18S | 25.4 | 50.013 | 6.649 | 25 | 0 | 78.343 | 21.657 | 14.905 | 1557.733 |
| ST1W16S | 29.7 | 38.511 | 2.691 | 5 | 0.591 | 88.851 | 10.558 | 5.372 | 715.699 |
| ST1W17S | 22.2 | 45.041 | 3.851 | 8 | 0 | 76.719 | 23.281 | 8.031 | 494.832 |
| ST2D17S | 24 | 62.810 | 8.873 | 26 | 0.587 | 72.382 | 27.031 | 13.162 | 1874.172 |
| ST2D18S | 25.4 | 57.013 | 7.538 | 25 | 0 | 83.820 | 16.180 | 12.182 | 2943.857 |
| ST2W16S | 29.7 | 42.141 | 2.870 | 5 | 0.266 | 88.906 | 10.827 | 6.446 | 883.115 |
| ST2W17S | 22.2 | 51.160 | 5.491 | 7 | 0.105 | 77.783 | 22.112 | 14.799 | 224.269 |
| ST3D17S | 24 | 68.440 | 11.299 | 25 | 0.019 | 75.816 | 24.165 | 16.645 | 2872.293 |
| ST3D18S | 25.4 | 47.961 | 5.919 | 21 | 12.886 | 80.758 | 6.356 | 7.141 | 1929.587 |
| ST3W16S | 29.7 | 42.571 | 3.870 | 6 | 1.115 | 89.606 | 9.279 | 7.790 | 913.659 |
| ST3W17S | 22.2 | 44.536 | 3.879 | 8 | 12.438 | 83.841 | 3.721 | 6.551 | 668.023 |
| VB1D17S | 20 | 28.420 | 2.461 | 38 | 0 | 94.366 | 5.634 | 2.417 | 994.850 |
| VB1D18S | 25.1 | 29.619 | 2.310 | 43 | 0.550 | 93.929 | 5.521 | 2.101 | 779.334 |
| VB1W16S | 31.5 | 30.722 | 2.890 | 37 | 0.793 | 94.576 | 4.631 | 1.880 | 1070.218 |
| VB1W17S | 24.7 | 25.855 | 1.709 | 21 | 2.708 | 95.222 | 2.070 | 1.691 | 705.417 |
| VB2D17S | 20 | 25.389 | 2.430 | 36 | 0 | 99.183 | 0.817 | 2.148 | 979.287 |
| VB2D18S | 25.1 | 28.233 | 1.990 | 42 | 1.461 | 93.525 | 5.013 | 1.680 | 494.716 |
| VB2W16S | 31.5 | 28.436 | 2.400 | 36 | 0.717 | 95.359 | 3.925 | 1.611 | 964.511 |
| VB2W17S | 24.7 | 28.659 | 2.031 | 24 | 0.679 | 94.926 | 4.395 | 2.114 | 1014.608 |
| Sample Name | Sediment Temperature (°C) | Water Content (%) | Total Organic Matter (%) | Porewater Salinity (ppt) | Gravel Fraction (%) | Sand Fraction (%) | Silt/Clay Fraction (%) | Cu (ppm) | Fe  (ppm) |
| VB3D17S | 20 | 27.269 | 2.110 | 38 | 0 | 93.479 | 6.521 | 2.148 | 964.414 |
| VB3D18S | 25.1 | 29.151 | 1.921 | 43 | 0.557 | 94.087 | 5.357 | 1.890 | 828.567 |
| VB3W16S | 31.5 | 29.243 | 2.370 | 38 | 1.511 | 94.345 | 4.144 | 1.611 | 904.048 |
| VB3W17S | 24.7 | 28.294 | 1.780 | 21 | 0.663 | 96.492 | 2.846 | 1.796 | 841.046 |
| VM1D18S | 24.4 | 37.812 | 4.891 | 41 | 16.873 | 71.108 | 12.020 | 10.068 | 2154.874 |
| VM1W17S | 24.4 | 44.843 | 5.160 | 27 | 2.017 | 79.024 | 18.959 | 12.857 | 2537.890 |
| VM2D18S | 24.4 | 35.766 | 4.291 | 41 | 6.992 | 81.119 | 11.889 | 9.233 | 1909.557 |
| VM2W17S | 24.4 | 54.104 | 7.272 | 28 | 1.525 | 71.874 | 26.601 | 18.747 | 3773.620 |
| VM3D18S | 24.4 | 38.638 | 4.371 | 42 | 3.451 | 78.809 | 17.739 | 10.700 | 2155.521 |
| VM3W17S | 24.4 | 50.683 | 6.830 | 27 | 1.573 | 71.237 | 27.191 | 17.072 | 3342.587 |

Sediment temperature was determined using a thermometer, water content by weight loss during oven drying, total organic matter by weight loss in a muffle furnace, grain size fractions (gravel, sand, and silt/clay) were determined using wet sieving, and copper (Cu) and iron (Fe) were measured using an atomic adsorption spectrometer. See manuscript for details.
