# Supplementary material for: Defining the sediment prokaryotic communities of the Indian River Lagoon, FL, USA, an Estuary of National Significance
Source: PLoS One. 2020 Oct 26;15(10):e0236305. doi: 10.1371/journal.pone.0236305 (PMC7588086; doi:10.1371/journal.pone.0236305)
Supplement: S3 Table — Table containing information pertaining to each sample such as when (Sampling Season, Season) and where (Site, Location, Estuary) it was taken. A sample was considered to have a muck characteristic if it exceeded one of the muck thresholds: 10% for total organic matter, 60% for silt/clay fraction, and 75% for water content [8]. Additionally, a sample was considered to have high copper if it exceeded 65 μg/g [70]. aIRL for Indian River Lagoon, bSLE for St. Lucie Estuary, cTOM stands for total organic matter, dCu for copper, eLoLo for low TOM/low Cu, fHiHi for high TOM/low Cu, gLoHi for low TOM/low Cu, and hHiLo stands for high TOM/low Cu. (DOCX) [file pone.0236305.s008.docx]

S3 Table: Metadata per sample

| Sample Name | Sampling Season | Season | Site | Location | Estuary | Muck Characteristics | TOM^c^/Cu^d^ Classification | Replicate |
| --- | --- | --- | --- | --- | --- | --- | --- | --- |
| BB1D18S | Apr 2018 | Dry | Barber Bridge | North Central | IRL^a^ | 0 | LoLo^e^ | 1 |
| BB1W17S | Oct-Nov 2017 | Wet | Barber Bridge | North Central | IRL | 0 | LoLo | 1 |
| BB2D18S | Apr 2018 | Dry | Barber Bridge | North Central | IRL | 0 | LoLo | 2 |
| BB2W17S | Oct-Nov 2017 | Wet | Barber Bridge | North Central | IRL | 0 | LoLo | 2 |
| BB3D18S | Apr 2018 | Dry | Barber Bridge | North Central | IRL | 0 | LoLo | 3 |
| BB3W17S | Oct-Nov 2017 | Wet | Barber Bridge | North Central | IRL | 0 | LoLo | 3 |
| FP1D17S | Mar-Apr 2017 | Dry | Fort Pierce | South Central | IRL | 0 | LoLo | 1 |
| FP1D18S | Apr 2018 | Dry | Fort Pierce | South Central | IRL | 0 | LoLo | 1 |
| FP1W16S | Aug-Sept 2016 | Wet | Fort Pierce | South Central | IRL | 0 | LoLo | 1 |
| FP1W17S-3 | Oct-Nov 2017 | Wet | Fort Pierce | South Central | IRL | 0 | LoLo | 1 |
| FP2D17S | Mar-Apr 2017 | Dry | Fort Pierce | South Central | IRL | 0 | LoLo | 2 |
| FP2D18S | Apr 2018 | Dry | Fort Pierce | South Central | IRL | 0 | LoLo | 2 |
| FP2W16S | Aug-Sept 2016 | Wet | Fort Pierce | South Central | IRL | 0 | LoLo | 2 |
| FP2W17S-3 | Oct-Nov 2017 | Wet | Fort Pierce | South Central | IRL | 0 | LoLo | 2 |
| FP3D17S | Mar-Apr 2017 | Dry | Fort Pierce | South Central | IRL | 0 | LoLo | 3 |
| FP3D18S | Apr 2018 | Dry | Fort Pierce | South Central | IRL | 0 | LoLo | 3 |
| FP3W16S | Aug-Sept 2016 | Wet | Fort Pierce | South Central | IRL | 0 | LoLo | 3 |
| FP3W17S-3 | Oct-Nov 2017 | Wet | Fort Pierce | South Central | IRL | 0 | LoLo | 3 |
| HB1D17S | Mar-Apr 2017 | Dry | Harbor Branch Channel | South Central | IRL | 2 | HiHi^f^ | 1 |
| HB1D18S | Apr 2018 | Dry | Harbor Branch Channel | South Central | IRL | 2 | HiHi | 1 |
| HB1W16S | Aug-Sept 2016 | Wet | Harbor Branch Channel | South Central | IRL | 3 | HiHi | 1 |
| HB1W17S-3 | Oct-Nov 2017 | Wet | Harbor Branch Channel | South Central | IRL | 2 | HiHi | 1 |
| HB2D17S | Mar-Apr 2017 | Dry | Harbor Branch Channel | South Central | IRL | 3 | HiHi | 2 |
| HB2D18S | Apr 2018 | Dry | Harbor Branch Channel | South Central | IRL | 2 | HiHi | 2 |
| HB2W16S | Aug-Sept 2016 | Wet | Harbor Branch Channel | South Central | IRL | 3 | HiHi | 2 |
| HB2W17S-3 | Oct-Nov 2017 | Wet | Harbor Branch Channel | South Central | IRL | 1 | LoHi^g^ | 2 |
| HB3D17S | Mar-Apr 2017 | Dry | Harbor Branch Channel | South Central | IRL | 1 | HiHi | 3 |
| HB3D18S | Apr 2018 | Dry | Harbor Branch Channel | South Central | IRL | 0 | LoHi | 3 |
| HB3W16S | Aug-Sept 2016 | Wet | Harbor Branch Channel | South Central | IRL | 3 | HiHi | 3 |
| HB3W17S-3 | Oct-Nov 2017 | Wet | Harbor Branch Channel | South Central | IRL | 3 | HiHi | 3 |
| HS1D17S | Mar-Apr 2017 | Dry | Hobe Sound | South | IRL | 0 | LoLo | 1 |
| HS1D18S | Apr 2018 | Dry | Hobe Sound | South | IRL | 0 | LoLo | 1 |
| HS1W16S | Aug-Sept 2016 | Wet | Hobe Sound | South | IRL | 0 | LoLo | 1 |
| HS1W17S | Oct-Nov 2017 | Wet | Hobe Sound | South | IRL | 0 | LoLo | 1 |
| HS2D17S | Mar-Apr 2017 | Dry | Hobe Sound | South | IRL | 0 | LoLo | 2 |
| HS2D18S | Apr 2018 | Dry | Hobe Sound | South | IRL | 0 | LoLo | 2 |
| HS2W16S | Aug-Sept 2016 | Wet | Hobe Sound | South | IRL | 0 | LoLo | 2 |
| HS2W17S | Oct-Nov 2017 | Wet | Hobe Sound | South | IRL | 0 | LoLo | 2 |
| HS3D17S | Mar-Apr 2017 | Dry | Hobe Sound | South | IRL | 0 | LoLo | 3 |
| HS3D18S | Apr 2018 | Dry | Hobe Sound | South | IRL | 0 | LoLo | 3 |
| HS3W16S | Aug-Sept 2016 | Wet | Hobe Sound | South | IRL | 0 | LoLo | 3 |
| HS3W17S | Oct-Nov 2017 | Wet | Hobe Sound | South | IRL | 0 | LoLo | 3 |
| HT1D18S | Apr 2018 | Dry | Harbortown Marina | South Central | IRL | 2 | HiLo^h^ | 1 |
| HT1W17S-3 | Oct-Nov 2017 | Wet | Harbortown Marina | South Central | IRL | 2 | HiHi | 1 |
| HT2D18S | Apr 2018 | Dry | Harbortown Marina | South Central | IRL | 1 | HiLo | 2 |
| HT2W17S-3 | Oct-Nov 2017 | Wet | Harbortown Marina | South Central | IRL | 3 | HiHi | 2 |
| HT3D18S | Apr 2018 | Dry | Harbortown Marina | South Central | IRL | 1 | HiLo | 3 |
| HT3W17S-3 | Oct-Nov 2017 | Wet | Harbortown Marina | South Central | IRL | 2 | HiHi | 3 |
| JB1D17S | Mar-Apr 2017 | Dry | Jensen Beach | South | IRL | 0 | LoLo | 1 |
| JB1D18S | Apr 2018 | Dry | Jensen Beach | South | IRL | 0 | LoLo | 1 |
| JB1W16S | Aug-Sept 2016 | Wet | Jensen Beach | South | IRL | 0 | LoLo | 1 |
| JB1W17S | Oct-Nov 2017 | Wet | Jensen Beach | South | IRL | 0 | LoLo | 1 |
| JB2D17S | Mar-Apr 2017 | Dry | Jensen Beach | South | IRL | 0 | LoLo | 2 |
| JB2D18S | Apr 2018 | Dry | Jensen Beach | South | IRL | 0 | LoLo | 2 |
| JB2W16S | Aug-Sept 2016 | Wet | Jensen Beach | South | IRL | 0 | LoLo | 2 |
| JB2W17S | Oct-Nov 2017 | Wet | Jensen Beach | South | IRL | 0 | LoLo | 2 |
| JB3D17S | Mar-Apr 2017 | Dry | Jensen Beach | South | IRL | 0 | LoLo | 3 |
| JB3D18S | Apr 2018 | Dry | Jensen Beach | South | IRL | 0 | LoLo | 3 |
| JB3W16S | Aug-Sept 2016 | Wet | Jensen Beach | South | IRL | 0 | LoLo | 3 |
| JB3W17S | Oct-Nov 2017 | Wet | Jensen Beach | South | IRL | 0 | LoLo | 3 |
| JN1D17S | Mar-Apr 2017 | Dry | Jupiter Narrows | South | IRL | 0 | LoLo | 1 |
| JN1D18S | Apr 2018 | Dry | Jupiter Narrows | South | IRL | 0 | LoLo | 1 |
| JN1W16S | Aug-Sept 2016 | Wet | Jupiter Narrows | South | IRL | 0 | LoLo | 1 |
| JN1W17S | Oct-Nov 2017 | Wet | Jupiter Narrows | South | IRL | 0 | LoLo | 1 |
| JN2D17S | Mar-Apr 2017 | Dry | Jupiter Narrows | South | IRL | 0 | LoLo | 2 |
| JN2D18S | Apr 2018 | Dry | Jupiter Narrows | South | IRL | 0 | LoLo | 2 |
| JN2W16S | Aug-Sept 2016 | Wet | Jupiter Narrows | South | IRL | 0 | LoLo | 2 |
| JN2W17S | Oct-Nov 2017 | Wet | Jupiter Narrows | South | IRL | 0 | LoLo | 2 |
| JN3D17S | Mar-Apr 2017 | Dry | Jupiter Narrows | South | IRL | 0 | LoLo | 3 |
| JN3D18S | Apr 2018 | Dry | Jupiter Narrows | South | IRL | 0 | LoLo | 3 |
| JN3W16S | Aug-Sept 2016 | Wet | Jupiter Narrows | South | IRL | 0 | LoLo | 3 |
| JN3W17S | Oct-Nov 2017 | Wet | Jupiter Narrows | South | IRL | 0 | LoLo | 3 |
| LP1D17S | Mar-Apr 2017 | Dry | Linkport | South Central | IRL | 0 | LoLo | 1 |
| LP1D18S | Apr 2018 | Dry | Linkport | South Central | IRL | 0 | LoLo | 1 |
| LP1W16S | Aug-Sept 2016 | Wet | Linkport | South Central | IRL | 0 | LoLo | 1 |
| LP1W17S-3 | Oct-Nov 2017 | Wet | Linkport | South Central | IRL | 0 | LoLo | 1 |
| LP2D17S | Mar-Apr 2017 | Dry | Linkport | South Central | IRL | 0 | LoLo | 2 |
| LP2D18S | Apr 2018 | Dry | Linkport | South Central | IRL | 1 | HiLo | 2 |
| LP2W16S | Aug-Sept 2016 | Wet | Linkport | South Central | IRL | 0 | LoLo | 2 |
| LP2W17S-3 | Oct-Nov 2017 | Wet | Linkport | South Central | IRL | 0 | LoLo | 2 |
| LP3D17S | Mar-Apr 2017 | Dry | Linkport | South Central | IRL | 0 | LoLo | 3 |
| LP3D18S | Apr 2018 | Dry | Linkport | South Central | IRL | 1 | HiLo | 3 |
| LP3W16S | Aug-Sept 2016 | Wet | Linkport | South Central | IRL | 0 | LoLo | 3 |
| LP3W17S-3 | Oct-Nov 2017 | Wet | Linkport | South Central | IRL | 0 | LoLo | 3 |
| MC1D17S | Mar-Apr 2017 | Dry | Melbourne Cswy | North | IRL | 3 | HiLo | 1 |
| MC1D18S | Apr 2018 | Dry | Melbourne Cswy | North | IRL | 0 | LoLo | 1 |
| MC1W16S | Aug-Sept 2016 | Wet | Melbourne Cswy | North | IRL | 3 | HiLo | 1 |
| MC1W17S | Oct-Nov 2017 | Wet | Melbourne Cswy | North | IRL | 2 | HiLo | 1 |
| MC2D17S | Mar-Apr 2017 | Dry | Melbourne Cswy | North | IRL | 3 | HiLo | 2 |
| MC2D18S | Apr 2018 | Dry | Melbourne Cswy | North | IRL | 0 | LoLo | 2 |
| MC2W16S | Aug-Sept 2016 | Wet | Melbourne Cswy | North | IRL | 3 | HiLo | 2 |
| MC2W17S | Oct-Nov 2017 | Wet | Melbourne Cswy | North | IRL | 0 | LoLo | 2 |
| MC3D17S | Mar-Apr 2017 | Dry | Melbourne Cswy | North | IRL | 1 | LoLo | 3 |
| MC3D18S | Apr 2018 | Dry | Melbourne Cswy | North | IRL | 3 | HiLo | 3 |
| MC3W16S | Aug-Sept 2016 | Wet | Melbourne Cswy | North | IRL | 2 | HiLo | 3 |
| MC3W17S | Oct-Nov 2017 | Wet | Melbourne Cswy | North | IRL | 2 | HiLo | 3 |
| ME1D17S | Mar-Apr 2017 | Dry | Middle Estuary | SLE | SLE^b^ | 3 | HiLo | 1 |
| ME1D18S | Apr 2018 | Dry | Middle Estuary | SLE | SLE | 3 | HiLo | 1 |
| ME1W16S | Aug-Sept 2016 | Wet | Middle Estuary | SLE | SLE | 3 | HiLo | 1 |
| ME1W17S | Oct-Nov 2017 | Wet | Middle Estuary | SLE | SLE | 3 | HiLo | 1 |
| ME2D17S | Mar-Apr 2017 | Dry | Middle Estuary | SLE | SLE | 3 | HiLo | 2 |
| ME2D18S | Apr 2018 | Dry | Middle Estuary | SLE | SLE | 3 | HiLo | 2 |
| ME2W16S | Aug-Sept 2016 | Wet | Middle Estuary | SLE | SLE | 2 | HiLo | 2 |
| ME2W17S | Oct-Nov 2017 | Wet | Middle Estuary | SLE | SLE | 3 | HiLo | 2 |
| ME3D17S | Mar-Apr 2017 | Dry | Middle Estuary | SLE | SLE | 3 | HiLo | 3 |
| ME3D18S | Apr 2018 | Dry | Middle Estuary | SLE | SLE | 3 | HiLo | 3 |
| ME3W16S | Aug-Sept 2016 | Wet | Middle Estuary | SLE | SLE | 2 | HiLo | 3 |
| ME3W17S | Oct-Nov 2017 | Wet | Middle Estuary | SLE | SLE | 3 | HiLo | 3 |
| MI1D17S | Mar-Apr 2017 | Dry | Merritt Island Cswy | North | IRL | 0 | LoLo | 1 |
| MI1D18S | Apr 2018 | Dry | Merritt Island Cswy | North | IRL | 0 | LoLo | 1 |
| MI1W16S | Aug-Sept 2016 | Wet | Merritt Island Cswy | North | IRL | 0 | LoLo | 1 |
| MI1W17S | Oct-Nov 2017 | Wet | Merritt Island Cswy | North | IRL | 0 | LoLo | 1 |
| MI2D17S | Mar-Apr 2017 | Dry | Merritt Island Cswy | North | IRL | 0 | LoLo | 2 |
| MI2D18S | Apr 2018 | Dry | Merritt Island Cswy | North | IRL | 0 | LoLo | 2 |
| MI2W16S | Aug-Sept 2016 | Wet | Merritt Island Cswy | North | IRL | 0 | LoLo | 2 |
| MI2W17S | Oct-Nov 2017 | Wet | Merritt Island Cswy | North | IRL | 0 | LoLo | 2 |
| MI3D17S | Mar-Apr 2017 | Dry | Merritt Island Cswy | North | IRL | 0 | LoLo | 3 |
| MI3D18S | Apr 2018 | Dry | Merritt Island Cswy | North | IRL | 0 | LoLo | 3 |
| MI3W16S | Aug-Sept 2016 | Wet | Merritt Island Cswy | North | IRL | 0 | LoLo | 3 |
| MI3W17S | Oct-Nov 2017 | Wet | Merritt Island Cswy | North | IRL | 0 | LoLo | 3 |
| MP1D17S | Mar-Apr 2017 | Dry | Manatee Pocket | South | IRL | 0 | LoLo | 1 |
| MP1D18S | Apr 2018 | Dry | Manatee Pocket | South | IRL | 1 | HiHi | 1 |
| MP1W16S | Aug-Sept 2016 | Wet | Manatee Pocket | South | IRL | 3 | HiHi | 1 |
| MP1W17S | Oct-Nov 2017 | Wet | Manatee Pocket | South | IRL | 3 | HiHi | 1 |
| MP2D17S | Mar-Apr 2017 | Dry | Manatee Pocket | South | IRL | 3 | HiHi | 2 |
| MP2D18S | Apr 2018 | Dry | Manatee Pocket | South | IRL | 3 | HiHi | 2 |
| MP2W16S | Aug-Sept 2016 | Wet | Manatee Pocket | South | IRL | 3 | HiHi | 2 |
| MP2W17S | Oct-Nov 2017 | Wet | Manatee Pocket | South | IRL | 3 | HiHi | 2 |
| MP3D17S | Mar-Apr 2017 | Dry | Manatee Pocket | South | IRL | 1 | HiHi | 3 |
| MP3D18S | Apr 2018 | Dry | Manatee Pocket | South | IRL | 2 | HiHi | 3 |
| MP3W16S | Aug-Sept 2016 | Wet | Manatee Pocket | South | IRL | 3 | HiHi | 3 |
| MP3W17S | Oct-Nov 2017 | Wet | Manatee Pocket | South | IRL | 3 | HiHi | 3 |
| NF1D17S | Mar-Apr 2017 | Dry | North Fork | SLE | SLE | 0 | LoLo | 1 |
| NF1D18S | Apr 2018 | Dry | North Fork | SLE | SLE | 0 | LoLo | 1 |
| NF1W16S | Aug-Sept 2016 | Wet | North Fork | SLE | SLE | 0 | LoLo | 1 |
| NF1W17S | Oct-Nov 2017 | Wet | North Fork | SLE | SLE | 0 | LoLo | 1 |
| NF2D17S | Mar-Apr 2017 | Dry | North Fork | SLE | SLE | 0 | LoLo | 2 |
| NF2D18S | Apr 2018 | Dry | North Fork | SLE | SLE | 0 | LoLo | 2 |
| NF2W16S | Aug-Sept 2016 | Wet | North Fork | SLE | SLE | 0 | LoLo | 2 |
| NF2W17S | Oct-Nov 2017 | Wet | North Fork | SLE | SLE | 0 | LoLo | 2 |
| NF3D17S | Mar-Apr 2017 | Dry | North Fork | SLE | SLE | 0 | LoLo | 3 |
| NF3D18S | Apr 2018 | Dry | North Fork | SLE | SLE | 0 | LoLo | 3 |
| NF3W16S | Aug-Sept 2016 | Wet | North Fork | SLE | SLE | 0 | LoLo | 3 |
| NF3W17S | Oct-Nov 2017 | Wet | North Fork | SLE | SLE | 0 | LoLo | 3 |
| RI1D18S | Apr 2018 | Dry | Round Island | North Central | IRL | 0 | LoLo | 1 |
| RI1W17S | Oct-Nov 2017 | Wet | Round Island | North Central | IRL | 0 | LoLo | 1 |
| RI2D18S | Apr 2018 | Dry | Round Island | North Central | IRL | 0 | LoLo | 2 |
| RI2W17S | Oct-Nov 2017 | Wet | Round Island | North Central | IRL | 0 | LoLo | 2 |
| RI3D18S | Apr 2018 | Dry | Round Island | North Central | IRL | 0 | LoLo | 3 |
| RI3W17S | Oct-Nov 2017 | Wet | Round Island | North Central | IRL | 0 | LoLo | 3 |
| SF1D17S | Mar-Apr 2017 | Dry | South Fork | SLE | SLE | 3 | HiLo | 1 |
| SF1D18S | Apr 2018 | Dry | South Fork | SLE | SLE | 3 | HiLo | 1 |
| SF1W16S | Aug-Sept 2016 | Wet | South Fork | SLE | SLE | 3 | HiLo | 1 |
| SF1W17S | Oct-Nov 2017 | Wet | South Fork | SLE | SLE | 3 | HiLo | 1 |
| SF2D17S | Mar-Apr 2017 | Dry | South Fork | SLE | SLE | 3 | HiLo | 2 |
| SF2D18S | Apr 2018 | Dry | South Fork | SLE | SLE | 3 | HiLo | 2 |
| SF2W16S | Aug-Sept 2016 | Wet | South Fork | SLE | SLE | 3 | HiLo | 2 |
| SF2W17S | Oct-Nov 2017 | Wet | South Fork | SLE | SLE | 3 | HiLo | 2 |
| SF3D17S | Mar-Apr 2017 | Dry | South Fork | SLE | SLE | 3 | HiLo | 3 |
| SF3D18S | Apr 2018 | Dry | South Fork | SLE | SLE | 3 | HiLo | 3 |
| SF3W16S | Aug-Sept 2016 | Wet | South Fork | SLE | SLE | 3 | HiLo | 3 |
| SF3W17S | Oct-Nov 2017 | Wet | South Fork | SLE | SLE | 2 | HiLo | 3 |
| SI1D17S | Mar-Apr 2017 | Dry | Sebastian Inlet | North | IRL | 0 | LoLo | 1 |
| SI1D18S | Apr 2018 | Dry | Sebastian Inlet | North | IRL | 0 | LoLo | 1 |
| SI1W16S | Aug-Sept 2016 | Wet | Sebastian Inlet | North | IRL | 0 | LoLo | 1 |
| SI1W17S | Oct-Nov 2017 | Wet | Sebastian Inlet | North | IRL | 0 | LoLo | 1 |
| SI2D17S | Mar-Apr 2017 | Dry | Sebastian Inlet | North | IRL | 0 | LoLo | 2 |
| SI2D18S | Apr 2018 | Dry | Sebastian Inlet | North | IRL | 0 | LoLo | 2 |
| SI2W16S | Aug-Sept 2016 | Wet | Sebastian Inlet | North | IRL | 0 | LoLo | 2 |
| SI2W17S | Oct-Nov 2017 | Wet | Sebastian Inlet | North | IRL | 0 | LoLo | 2 |
| SI3D17S | Mar-Apr 2017 | Dry | Sebastian Inlet | North | IRL | 0 | LoLo | 3 |
| SI3D18S | Apr 2018 | Dry | Sebastian Inlet | North | IRL | 0 | LoLo | 3 |
| SI3W16S | Aug-Sept 2016 | Wet | Sebastian Inlet | North | IRL | 0 | LoLo | 3 |
| SI3W17S | Oct-Nov 2017 | Wet | Sebastian Inlet | North | IRL | 0 | LoLo | 3 |
| ST1D17S | Mar-Apr 2017 | Dry | South Fork 2 | SLE | SLE | 0 | LoLo | 1 |
| ST1D18S | Apr 2018 | Dry | South Fork 2 | SLE | SLE | 0 | LoLo | 1 |
| ST1W16S | Aug-Sept 2016 | Wet | South Fork 2 | SLE | SLE | 0 | LoLo | 1 |
| ST1W17S | Oct-Nov 2017 | Wet | South Fork 2 | SLE | SLE | 0 | LoLo | 1 |
| ST2D17S | Mar-Apr 2017 | Dry | South Fork 2 | SLE | SLE | 0 | LoLo | 2 |
| ST2D18S | Apr 2018 | Dry | South Fork 2 | SLE | SLE | 0 | LoLo | 2 |
| ST2W16S | Aug-Sept 2016 | Wet | South Fork 2 | SLE | SLE | 0 | LoLo | 2 |
| ST2W17S | Oct-Nov 2017 | Wet | South Fork 2 | SLE | SLE | 0 | LoLo | 2 |
| ST3D17S | Mar-Apr 2017 | Dry | South Fork 2 | SLE | SLE | 1 | HiLo | 3 |
| ST3D18S | Apr 2018 | Dry | South Fork 2 | SLE | SLE | 0 | LoLo | 3 |
| ST3W16S | Aug-Sept 2016 | Wet | South Fork 2 | SLE | SLE | 0 | LoLo | 3 |
| ST3W17S | Oct-Nov 2017 | Wet | South Fork 2 | SLE | SLE | 0 | LoLo | 3 |
| VB1D17S | Mar-Apr 2017 | Dry | Vero Beach | North Central | IRL | 0 | LoLo | 1 |
| VB1D18S | Apr 2018 | Dry | Vero Beach | North Central | IRL | 0 | LoLo | 1 |
| VB1W16S | Aug-Sept 2016 | Wet | Vero Beach | North Central | IRL | 0 | LoLo | 1 |
| VB1W17S | Oct-Nov 2017 | Wet | Vero Beach | North Central | IRL | 0 | LoLo | 1 |
| VB2D17S | Mar-Apr 2017 | Dry | Vero Beach | North Central | IRL | 0 | LoLo | 2 |
| VB2D18S | Apr 2018 | Dry | Vero Beach | North Central | IRL | 0 | LoLo | 2 |
| VB2W16S | Aug-Sept 2016 | Wet | Vero Beach | North Central | IRL | 0 | LoLo | 2 |
| VB2W17S | Oct-Nov 2017 | Wet | Vero Beach | North Central | IRL | 0 | LoLo | 2 |
| VB3D17S | Mar-Apr 2017 | Dry | Vero Beach | North Central | IRL | 0 | LoLo | 3 |
| VB3D18S | Apr 2018 | Dry | Vero Beach | North Central | IRL | 0 | LoLo | 3 |
| VB3W16S | Aug-Sept 2016 | Wet | Vero Beach | North Central | IRL | 0 | LoLo | 3 |
| VB3W17S | Oct-Nov 2017 | Wet | Vero Beach | North Central | IRL | 0 | LoLo | 3 |
| VM1D18S | Apr 2018 | Dry | Vero Beach Marina | North Central | IRL | 0 | LoLo | 1 |
| VM1W17S | Oct-Nov 2017 | Wet | Vero Beach Marina | North Central | IRL | 0 | LoLo | 1 |
| VM2D18S | Apr 2018 | Dry | Vero Beach Marina | North Central | IRL | 0 | LoLo | 2 |
| VM2W17S | Oct-Nov 2017 | Wet | Vero Beach Marina | North Central | IRL | 0 | LoLo | 2 |
| VM3D18S | Apr 2018 | Dry | Vero Beach Marina | North Central | IRL | 0 | LoLo | 3 |
| VM3W17S | Oct-Nov 2017 | Wet | Vero Beach Marina | North Central | IRL | 0 | LoLo | 3 |

Table containing information pertaining to each sample such as when (Sampling Season, Season) and where (Site, Location, Estuary) it was taken. A sample was considered to have a muck characteristic if it exceeded one of the muck thresholds: 10% for total organic matter, 60% for silt/clay fraction, and 75 % for water content [8]. Additionally, a sample was considered to have high copper if it exceeded 65 µg/g [70]. ^a^IRL for Indian River Lagoon, ^b^SLE for St. Lucie Estuary, ^c^TOM stands for total organic matter, ^d^Cu for copper, ^e^LoLo for low TOM/low Cu, ^f^HiHi for high TOM/low Cu, ^g^LoHi for low TOM/low Cu, and ^h^HiLo stands for high TOM/low Cu.
