# Supplementary material for: Defining the sediment prokaryotic communities of the Indian River Lagoon, FL, USA, an Estuary of National Significance
Source: PLoS One. 2020 Oct 26;15(10):e0236305. doi: 10.1371/journal.pone.0236305 (PMC7588086; doi:10.1371/journal.pone.0236305)
Supplement: S4 Table — The average streamflow from all fourteen stream/canals separately (A), averaged together regionally (B), and all together (C) during the months before and during each sampling period. Data was taken from the *United States Geological Services online database [32] or the **South Florida Water Management District’s DBHYDRO online database [31]. aThe regional location each canal/stream was found in, bdata from the month before and months during each sampling period and cthe entire survey. dIRL stands for Indian River Lagoon. (DOCX) [file pone.0236305.s009.docx]

S4 Table: Average monthly means for canal daily discharges (ft^3^/s)

A)

| Stream/Canal | Location^a^ | 07/16-09/16^b^ | 02/17-04/17^b^ | 09/17-11/17^b^ | 03/18-04/18^b^ | 06/16-06/18^c^ |
| --- | --- | --- | --- | --- | --- | --- |
| Eau Gallie River* | North IRL^d^ | 14 +/- 13 | 5.7 +/- 2.6 | 41 +/- 25 | 6.8 +/- 0.18 | 15 +/- 16 |
| Crane Creek* | North IRL | 43 +/- 13 | 9.8 +/- 3.3 | 95 +/- 64 | 8.5 +/- 2.2 | 32 +/- 34 |
| Turkey Creek* | North IRL | 211 +/- 206 | 38 +/- 14 | 667 +/- 400 | 41 +/- 9.0 | 192 +/- 250 |
| North Prong Sebastian River* | North IRL | 76 +/- 61 | 8.4 +/- 2.0 | 267 +/- 125 | 13 +/- 2.9 | 74 +/- 91 |
| Fellsmere Canal* | North IRL | 104 +/- 37 | 41 +/- 5.5 | 253 +/- 103 | 47 +/- 1.6 | 106 +/- 76 |
| South Prong Sebastian River* | North IRL | 89 +/- 32 | 33 +/- 2.2 | 415 +/- 171 | 37 +/- 1.2 | 129 +/- 132 |
| North Canal* | North Central IRL | 40 +/- 18 | 15 +/- 1.8 | 141 +/- 43 | 16 +/- 0.71 | 49 +/- 43 |
| Main Canal* | North Central IRL | 76 +/- 47 | 16 +/- 2.9 | 188 +/- 87 | 11 +/- 12 | 68 +/- 67 |
| South Canal* | North Central IRL | 76 +/- 40 | 22 +/- 1.1 | 182 +/- 87 | 43 +/- 4.6 | 67 +/- 56 |
| C25** | South Central IRL | 518 +/- 274 | 0.028 +/- 0.048 | 968 +/- 652 | 16 | 378 +/- 461 |
| Ten Mile Creek** | St. Lucie Estuary | 265 +/- 187 | 51 +/- 5.8 | 436 +/- 134 | 66 +/- 4.5 | 192 +/- 150 |
| C24** | St. Lucie Estuary | 190 +/- 228 | 0.0027 +/- 0.0046 | 835 +/- 432 | 0 | 242 +/- 319 |
| C23** | St. Lucie Estuary | 154 +/- 97 | 0.10 +/- 0.12 | 857 +/- 474 | 0.034 +/- 0.047 | 227 +/- 322 |
| C44** | St. Lucie Estuary | 905 +/- 266 | 0 | 3282 +/- 929 | 0.97 +/- 1.4 | 792 +/- 1141 |

B)

| Region | 07/16-09/16 | 02/17-04/17 | 09/17-11/17 | 03/18-04/18 | 06/16-06/18 |
| --- | --- | --- | --- | --- | --- |
| IRL | 125 +/- 171 | 19 +/- 14 | 322 +/- 352 | 24 +/- 16 | 109 +/- 200 |
| SLE | 378 +/- 365 | 12 +/- 23 | 1352 +/- 1273 | 17 +/- 30 | 363 +/- 658 |

C)

| Period | 07/16-09/16 | 02/17-04/17 | 09/17-11/17 | 03/18-04/18 |
| --- | --- | --- | --- | --- |
| Period Average | 197 +/- 264 | 17 +/- 17 | 616 +/- 863 | 22 +/- 21 |

The average streamflow from all fourteen stream/canals separately (A), averaged together regionally (B), and all together (C) during the months before and during each sampling period. Data was taken from the *United States Geological Services online database [32] or the **South Florida Water Management District’s DBHYDRO online database [31]. ^a^The regional location each canal/stream was found in, ^b^data from the month before and months during each sampling period and ^c^the entire survey. ^d^IRL stands for Indian River Lagoon.
