# Supplementary material for: Defining the sediment prokaryotic communities of the Indian River Lagoon, FL, USA, an Estuary of National Significance
Source: PLoS One. 2020 Oct 26;15(10):e0236305. doi: 10.1371/journal.pone.0236305 (PMC7588086; doi:10.1371/journal.pone.0236305)
Supplement: S5 Table — aBold text is associated with testing the overall differences within a category with Kruskal-Wallis. bRegular text is associated with pair-wise Dunn testing. cBH stands for Benjamini-Hochberg, dIRL stands for Indian River Lagoon and eSLE stands for St. Lucie Estuary. (DOCX) [file pone.0236305.s010.docx]

S5 Table: Environmental parameters statistical analysis

| **Kruskal-Wallis Overall Test Category^a^** | **chi²** | p value | BH^c^-adjusted  p value |
| --- | --- | --- | --- |
| Dunn Pair-wise test category^b^ | Z |  |  |
| **IRL^d^ Porewater Salinity by Sampling Season** | **96** | **<2.2E-16** | **<1.1E-15** |
| IRL Mar/Apr 2017 - IRL Apr 2018 | -1.4 | 0.16 | 0.16 |
| IRL Mar/Apr 2017 - IRL Aug/Sept 2016 | 2.3 | 0.022 | 0.026 |
| IRL Apr 2018 - IRL Aug/Sept 2016 | 3.9 | 0.00011 | 0.00016 |
| IRL Mar/Apr 2017 - IRL Oct/Nov 2017 | 7.1 | 1.3E-12 | 3.9E-12 |
| IRL Apr 2018 - IRL Oct/Nov 2017 | 9.2 | 2.3E-20 | 1.4E-19 |
| IRL Aug/Sept 2016 - IRL Oct/Nov 2017 | 4.6 | 3.6E-06 | 7.2E-06 |
| **SLE^e^ Porewater Salinity by Sampling Season** | **38** | **3.5E-08** | **7.5E-08** |
| SLE Mar/Apr 2017 - SLE Apr 2018 | -0.45 | 0.65 | 0.65 |
| SLE Mar/Apr 2017 - SLE Aug/Sept 2016 | 3.3 | 0.00083 | 0.00124 |
| SLE Apr 2018 - SLE Aug/Sept 2016 | 3.8 | 0.00015 | 0.00029 |
| SLE Mar/Apr 2017 - SLE Oct/Nov 2017 | 4.6 | 3.4E-06 | 1.0E-05 |
| SLE Apr 2018 - SLE Oct/Nov 2017 | 5.1 | 3.4E-07 | 2.0E-06 |
| SLE Aug/Sept 2016 - SLE Oct/Nov 2017 | 1.3 | 0.19 | 0.23 |
| **Porewater Salinity by Location** | **63** | **0.0014** | **0.0023** |
| North IRL - North Central IRL | -0.16 | 0.88 | 0.88 |
| North IRL - SLE | 4.4 | 1.2E-05 | 3.9E-05 |
| North Central IRL - SLE | 4.3 | 1.6E-05 | 4.0E-05 |
| North IRL – South IRL | -2.3 | 0.022 | 0.045 |
| North Central IRL – South IRL | -2.0 | 0.046 | 0.076 |
| SLE – South IRL | -7.2 | 6.0E-13 | 6.0E-12 |
| North IRL - South Central IRL | -1.6 | 0.10 | 0.14 |
| North Central IRL - South Central IRL | -1.4 | 0.16 | 0.20 |
| SLE - South Central IRL | -6.3 | 2.4E-10 | 1.2E-09 |
| South IRL - South Central IRL | 0.62 | 0.53 | 0.59 |
| **IRL Sediment Temperature by Sampling Season** | **86** | **<2.2E-16** | **<1.1E-15** |
| IRL Mar/Apr 2017 - IRL Apr 2018 | -2.6 | 0.010 | 0.015 |
| IRL Mar/Apr 2017 - IRL Aug/Sept 2016 | -8.1 | 6.8E-16 | 2.1E-15 |
| IRL Apr 2018 - IRL Aug/Sept 2016 | -6.1 | 1.1E-09 | 2.1E-09 |
| IRL Mar/Apr 2017 - IRL Oct/Nov 2017 | -0.52 | 0.60 | 0.60 |
| IRL Apr 2018 - IRL Oct/Nov 2017 | 2.2 | 0.026 | 0.031 |
| IRL Aug/Sept 2016 - IRL Oct/Nov 2017 | 8.1 | 3.7E-16 | 2.2E-15 |
| **SLE Sediment Temperature by Sampling Season** | **45** | **1.1E-09** | **2.9E-09** |
| SLE Mar/Apr 2017 - SLE Apr 2018 | -2.1 | 0.035 | 0.035 |
| SLE Mar/Apr 2017 - SLE Aug/Sept 2016 | -4.2 | 2.4E-05 | 4.8E-05 |
| SLE Apr 2018 - SLE Aug/Sept 2016 | -2.1 | 0.035 | 0.042 |
| SLE Mar/Apr 2017 - SLE Oct/Nov 2017 | 2.1 | 0.035 | 0.052 |
| SLE Apr 2018 - SLE Oct/Nov 2017 | 4.2 | 2.4E-05 | 7.3E-05 |
| SLE Aug/Sept 2016 - SLE Oct/Nov 2017 | 6.3 | 2.4E-10 | 1.4E-09 |
| **Sediment Temperature by Location** | **18** | **7.8E-13** | **2.4E-12** |
| North IRL - North Central IRL | 0.043 | 0.97 | 0.97 |
| North IRL - SLE | -0.64 | 0.52 | 0.58 |
| North Central IRL - SLE | -0.66 | 0.51 | 0.64 |
| North IRL - South IRL | -2.3 | 0.020 | 0.067 |
| North Central IRL - South IRL | -2.2 | 0.025 | 0.062 |
| SLE - South IRL | -1.8 | 0.070 | 0.14 |
| North - South Central IRL | 1.6 | 0.11 | 0.19 |
| North Central IRL - South Central IRL | 1.5 | 0.14 | 0.21 |
| SLE - South Central IRL | 2.4 | 0.018 | 0.088 |
| South IRL - South Central IRL | 4.1 | 3.7E-05 | 0.0003719 |

^a^Bold text is associated with testing the overall differences within a category with Kruskal-Wallis. ^b^Regular text is associated with pair-wise Dunn testing. ^c^BH stands for Benjamini-Hochberg, ^d^IRL stands for Indian River Lagoon and ^e^SLE stands for St. Lucie Estuary.
