# Supplementary material for: Defining the sediment prokaryotic communities of the Indian River Lagoon, FL, USA, an Estuary of National Significance
Source: PLoS One. 2020 Oct 26;15(10):e0236305. doi: 10.1371/journal.pone.0236305 (PMC7588086; doi:10.1371/journal.pone.0236305)
Supplement: S6 Table — aBold text is associated with testing the overall differences within a category with Kruskal-Wallis or Mann-Whitney use with a * indicating the latter was used. bRegular text is associated with pair-wise Dunn testing. cBH stands for Benjami-Hochberg, dTOM for total organic matter, eCu for copper, fIRL for Indian River Lagoon and gSLE for St. Lucie Estuary. (DOCX) [file pone.0236305.s011.docx]

S6 Table: Shannon diversity statistical analysis

| **Mann Whitney U/Kruskal-Wallis Overall Test Category^a^** | **W*/chi²** | p value | BH^c^-adjusted  p value |
| --- | --- | --- | --- |
| Dunn Pair-wise test category^b^ | Z |  |  |
| **Estuary** | **2952*** | **0.027** | **0.034^a^** |
| **Season** | **5935*** | **0.082** | **0.095** |
| **Muck Characteristics** | **2.9** | **0.40** | **0.43** |
| 3 - 1 | 1.7 | 0.094 | 0.56 |
| 3 - 2 | 0.018 | 0.99 | 0.99 |
| 1 - 2 | -1.3 | 0.21 | 0.42 |
| 3 - 0 | 0.63 | 0.53 | 0.79 |
| 1 - 0 | -1.5 | 0.15 | 0.44 |
| 2 - 0 | 0.32 | 0.75 | 0.90 |
| **TOM/Cu** | **0.49** | **0.92** | **0.92** |
| High TOM^d^/High Cu^e^ - High TOM/Low Cu | -0.22 | 0.83 | 0.99 |
| High TOM/High Cu - Low TOM/High Cu | 0.59 | 0.55 | 1.0 |
| High TOM/Low Cu - Low TOM/High Cu | 0.68 | 0.50 | 1.0 |
| High TOM/High Cu - Low TOM/Low Cu | -0.0023 | 1.00 | 1.00 |
| High TOM/Low Cu - Low TOM/Low Cu | 0.31 | 0.76 | 1.0 |
| Low TOM/High Cu - Low TOM/Low Cu | -0.61 | 0.54 | 1.0 |
| **Site** | **34** | **0.014** | **0.020** |
| Merritt Island Causeway - South Fork | -3.3 | 0.0011 | 0.18 |
| Merritt Island Causeway - North Fork | -3.2 | 0.0013 | 0.11 |
| Merritt Island Causeway - Vero Beach | -3.1 | 0.0022 | 0.12 |
| Melbourne Causeway - South Fork | -3.0 | 0.0025 | 0.11 |
| Jupiter Narrows - Merritt Island Causeway | 3.0 | 0.0026 | 0.089 |
| Melbourne Causeway - North Fork | -3.0 | 0.0030 | 0.086 |
| Fort Pierce - Merritt Island Causeway | 2.9 | 0.0038 | 0.093 |
| Linkport - Merritt Island Causeway | 2.9 | 0.0040 | 0.086 |
| Melbourne Causeway - Vero Beach | -2.8 | 0.0049 | 0.093 |
| Jupiter Narrows - Melbourne Causeway | 2.8 | 0.0057 | 0.098 |
| Fort Pierce - Melbourne Causeway | 2.6 | 0.0082 | 0.13 |
| Linkport - Melbourne Causeway | 2.6 | 0.0086 | 0.12 |
| Harbor Branch Channel - Merritt Island Causeway | 2.6 | 0.010 | 0.14 |
| Merritt Island Causeway - Middle Estuary | -2.5 | 0.014 | 0.17 |
| Harbor Branch Channel - Melbourne Causeway | 2.3 | 0.021 | 0.24 |
| Merritt Island Causeway - Sebastian Inlet | -2.3 | 0.023 | 0.24 |
| Hobe Sound - Merritt Island Causeway | 2.2 | 0.027 | 0.27 |
| Barber Bridge - South Fork | -2.2 | 0.027 | 0.26 |
| Melbourne Causeway - Middle Estuary | -2.2 | 0.027 | 0.24 |
| Merritt Island Causeway - South Fork 2 | -2.2 | 0.028 | 0.24 |
| Barber Bridge - North Fork | -2.2 | 0.030 | 0.25 |
| **Mann Whitney U/Kruskal-Wallis Overall Test Category** | **W*/chi²** | p value | BH-adjusted  p value |
| Dunn Pair-wise test category | Z |  |  |
| **Site** | | | |
| Barber Bridge - Vero Beach | -2.0 | 0.041 | 0.32 |
| Melbourne Causeway - Sebastian Inlet | -2.0 | 0.043 | 0.32 |
| Barber Bridge - Jupiter Narrows | -2.0 | 0.045 | 0.32 |
| Hobe Sound - Melbourne Causeway | 2.0 | 0.049 | 0.34 |
| Melbourne Causeway - South Fork 2 | -2.0 | 0.051 | 0.33 |
| Round Island - South Fork | -1.9 | 0.052 | 0.33 |
| Barber Bridge - Fort Pierce | -1.9 | 0.057 | 0.35 |
| North Fork - Round Island | 1.9 | 0.058 | 0.34 |
| Barber Bridge - Linkport | -1.9 | 0.059 | 0.33 |
| South Fork - Vero Beach Marina | 1.8 | 0.073 | 0.41 |
| Round Island - Vero Beach | -1.8 | 0.076 | 0.41 |
| North Fork - Vero Beach Marina | 1.7 | 0.081 | 0.42 |
| Manatee Pocket - Merritt Island Causeway | 1.7 | 0.081 | 0.41 |
| Jensen Beach - South Fork | -1.7 | 0.082 | 0.40 |
| Jupiter Narrows - Round Island | 1.7 | 0.084 | 0.40 |
| Jensen Beach - North Fork | -1.7 | 0.092 | 0.43 |
| Barber Bridge - Harbor Branch Channel | -1.6 | 0.10 | 0.45 |
| Fort Pierce - Round Island | 1.6 | 0.10 | 0.46 |
| Linkport - Round Island | 1.6 | 0.11 | 0.44 |
| Vero Beach - Vero Beach Marina | 1.6 | 0.11 | 0.45 |
| Jupiter Narrows - Vero Beach Marina | 1.6 | 0.11 | 0.47 |
| Barber Bridge - Middle Estuary | -1.5 | 0.12 | 0.48 |
| Jensen Beach - Merritt Island Causeway | 1.5 | 0.13 | 0.48 |
| Jensen Beach - Vero Beach | -1.5 | 0.13 | 0.49 |
| Manatee Pocket - South Fork | -1.5 | 0.13 | 0.47 |
| Manatee Pocket - Melbourne Causeway | 1.5 | 0.14 | 0.49 |
| Fort Pierce - Vero Beach Marina | 1.5 | 0.14 | 0.49 |
| Jensen Beach - Jupiter Narrows | -1.5 | 0.14 | 0.48 |
| Harbortown Marina - Merritt Island Causeway | 1.5 | 0.14 | 0.48 |
| Manatee Pocket - North Fork | -1.5 | 0.14 | 0.47 |
| Linkport - Vero Beach Marina | 1.5 | 0.14 | 0.47 |
| Barber Bridge - Sebastian Inlet | -1.4 | 0.16 | 0.52 |
| Harbor Branch Channel - Round Island | 1.4 | 0.17 | 0.55 |
| Fort Pierce - Jensen Beach | 1.4 | 0.17 | 0.54 |
| Barber Bridge - Hobe Sound | -1.4 | 0.18 | 0.54 |
| Jensen Beach - Linkport | -1.3 | 0.18 | 0.54 |
| Barber Bridge - South Fork 2 | -1.3 | 0.18 | 0.53 |
| Manatee Pocket - Vero Beach | -1.3 | 0.19 | 0.54 |
| Jensen Beach - Melbourne Causeway | 1.3 | 0.20 | 0.57 |
| Middle Estuary - Round Island | 1.3 | 0.20 | 0.56 |
| **Mann Whitney U/Kruskal-Wallis Overall Test Category** | **W*/chi²** | p value | BH-adjusted  p value |
| Dunn Pair-wise test category | Z |  |  |
| **Site** | | | |
| Harbortown Marina - Melbourne Causeway | 1.3 | 0.20 | 0.56 |
| Jupiter Narrows - Manatee Pocket | 1.3 | 0.20 | 0.55 |
| Harbor Branch Channel - Vero Beach Marina | 1.2 | 0.23 | 0.60 |
| Harbortown Marina - South Fork | -1.2 | 0.23 | 0.61 |
| Harbortown Marina - North Fork | -1.2 | 0.25 | 0.65 |
| Fort Pierce - Manatee Pocket | 1.2 | 0.25 | 0.64 |
| Linkport - Manatee Pocket | 1.1 | 0.26 | 0.65 |
| Round Island - Sebastian Inlet | -1.1 | 0.26 | 0.64 |
| Middle Estuary - Vero Beach Marina | 1.1 | 0.26 | 0.64 |
| Hobe Sound - Round Island | 1.1 | 0.28 | 0.67 |
| Round Island - South Fork 2 | -1.1 | 0.28 | 0.68 |
| South Fork - South Fork 2 | 1.1 | 0.29 | 0.67 |
| Hobe Sound - South Fork | -1.1 | 0.29 | 0.67 |
| Harbor Branch Channel - Jensen Beach | 1.0 | 0.30 | 0.69 |
| Harbortown Marina - Vero Beach | -1.0 | 0.30 | 0.68 |
| North Fork - South Fork 2 | 1.0 | 0.31 | 0.69 |
| Hobe Sound - North Fork | -1.0 | 0.32 | 0.70 |
| Sebastian Inlet - South Fork | -1.0 | 0.32 | 0.69 |
| Harbortown Marina - Jupiter Narrows | -0.99 | 0.32 | 0.69 |
| Sebastian Inlet - Vero Beach Marina | 0.98 | 0.33 | 0.69 |
| Barber Bridge - Manatee Pocket | -0.97 | 0.33 | 0.70 |
| North Fork - Sebastian Inlet | 0.94 | 0.35 | 0.71 |
| Hobe Sound - Vero Beach Marina | 0.93 | 0.35 | 0.72 |
| Jensen Beach - Middle Estuary | -0.93 | 0.35 | 0.71 |
| South Fork 2 - Vero Beach Marina | 0.92 | 0.36 | 0.71 |
| Fort Pierce - Harbortown Marina | 0.89 | 0.37 | 0.73 |
| Merritt Island Causeway - Vero Beach Marina | -0.88 | 0.38 | 0.74 |
| Barber Bridge - Harbortown Marina | -0.88 | 0.38 | 0.73 |
| Harbortown Marina - Linkport | -0.88 | 0.38 | 0.72 |
| South Fork 2 - Vero Beach | -0.86 | 0.39 | 0.73 |
| Hobe Sound - Vero Beach | -0.85 | 0.40 | 0.74 |
| Harbor Branch Channel - Manatee Pocket | 0.82 | 0.41 | 0.76 |
| Middle Estuary - South Fork | -0.81 | 0.42 | 0.76 |
| Jupiter Narrows - South Fork 2 | 0.81 | 0.42 | 0.75 |
| Hobe Sound - Jupiter Narrows | -0.80 | 0.43 | 0.76 |
| Barber Bridge - Jensen Beach | -0.79 | 0.43 | 0.75 |
| Sebastian Inlet - Vero Beach | -0.79 | 0.43 | 0.75 |
| Middle Estuary - North Fork | -0.76 | 0.45 | 0.78 |
| Jensen Beach - Sebastian Inlet | -0.74 | 0.46 | 0.78 |
| Jupiter Narrows - Sebastian Inlet | 0.74 | 0.46 | 0.78 |
| **Mann Whitney U/Kruskal-Wallis Overall Test Category** | **W*/chi²** | p value | BH-adjusted  p value |
| Dunn Pair-wise test category | Z |  |  |
| **Site** | | | |
| Merritt Island Causeway - Round Island | -0.73 | 0.47 | 0.78 |
| Manatee Pocket - Middle Estuary | -0.72 | 0.47 | 0.79 |
| Harbor Branch Channel - South Fork | -0.71 | 0.48 | 0.79 |
| Manatee Pocket - Round Island | 0.69 | 0.49 | 0.79 |
| Fort Pierce - South Fork 2 | 0.69 | 0.49 | 0.79 |
| Hobe Sound - Jensen Beach | 0.68 | 0.49 | 0.79 |
| Fort Pierce - Hobe Sound | 0.68 | 0.50 | 0.79 |
| Melbourne Causeway - Vero Beach Marina | -0.68 | 0.50 | 0.78 |
| Linkport - South Fork 2 | 0.67 | 0.50 | 0.78 |
| Jensen Beach - South Fork 2 | -0.67 | 0.50 | 0.77 |
| Hobe Sound - Linkport | -0.66 | 0.51 | 0.78 |
| Harbor Branch Channel - North Fork | -0.65 | 0.51 | 0.78 |
| Harbortown Marina - Round Island | 0.65 | 0.52 | 0.78 |
| Fort Pierce - Sebastian Inlet | 0.62 | 0.54 | 0.80 |
| Harbor Branch Channel - Harbortown Marina | 0.62 | 0.54 | 0.79 |
| Middle Estuary - Vero Beach | -0.61 | 0.55 | 0.80 |
| Linkport - Sebastian Inlet | 0.60 | 0.55 | 0.79 |
| Jupiter Narrows - Middle Estuary | 0.55 | 0.58 | 0.83 |
| Manatee Pocket - Vero Beach Marina | 0.54 | 0.59 | 0.84 |
| Harbortown Marina - Middle Estuary | -0.53 | 0.59 | 0.84 |
| Manatee Pocket - Sebastian Inlet | -0.53 | 0.59 | 0.83 |
| Melbourne Causeway - Round Island | -0.53 | 0.60 | 0.83 |
| Jensen Beach - Round Island | 0.52 | 0.60 | 0.83 |
| Harbortown Marina - Vero Beach Marina | 0.51 | 0.61 | 0.83 |
| Harbor Branch Channel - Vero Beach | -0.50 | 0.62 | 0.84 |
| Hobe Sound - Manatee Pocket | 0.47 | 0.64 | 0.86 |
| Manatee Pocket - South Fork 2 | -0.46 | 0.65 | 0.86 |
| Barber Bridge - Merritt Island Causeway | 0.46 | 0.65 | 0.86 |
| Harbor Branch Channel - Jupiter Narrows | -0.45 | 0.65 | 0.86 |
| Fort Pierce - Middle Estuary | 0.44 | 0.66 | 0.87 |
| Linkport - Middle Estuary | 0.42 | 0.68 | 0.88 |
| Linkport - South Fork | -0.39 | 0.69 | 0.89 |
| Harbortown Marina - Sebastian Inlet | -0.38 | 0.70 | 0.89 |
| Fort Pierce - South Fork | -0.38 | 0.71 | 0.89 |
| Jensen Beach - Vero Beach Marina | 0.37 | 0.71 | 0.89 |
| Barber Bridge - Vero Beach Marina | -0.37 | 0.71 | 0.89 |
| Harbor Branch Channel - South Fork 2 | 0.36 | 0.72 | 0.89 |
| Harbor Branch Channel - Hobe Sound | 0.35 | 0.73 | 0.90 |
| Linkport - North Fork | -0.34 | 0.73 | 0.90 |
| Harbortown Marina - Hobe Sound | -0.34 | 0.74 | 0.89 |
| **Mann Whitney U/Kruskal-Wallis Overall Test Category** | **W*/chi²** | p value | BH-adjusted  p value |
| Dunn Pair-wise test category | Z |  |  |
| **Site** | | | |
| Fort Pierce - Harbor Branch Channel | 0.33 | 0.74 | 0.89 |
| Harbortown Marina - South Fork 2 | -0.32 | 0.75 | 0.89 |
| Fort Pierce - North Fork | -0.32 | 0.75 | 0.89 |
| Harbor Branch Channel - Linkport | -0.31 | 0.75 | 0.89 |
| Harbor Branch Channel - Sebastian Inlet | 0.29 | 0.77 | 0.91 |
| Jupiter Narrows - South Fork | -0.26 | 0.80 | 0.93 |
| Middle Estuary - South Fork 2 | 0.26 | 0.80 | 0.92 |
| Barber Bridge - Melbourne Causeway | 0.25 | 0.80 | 0.92 |
| Melbourne Causeway - Merritt Island Causeway | 0.25 | 0.80 | 0.92 |
| Hobe Sound - Middle Estuary | -0.24 | 0.81 | 0.92 |
| Barber Bridge - Round Island | -0.23 | 0.81 | 0.92 |
| Harbortown Marina - Jensen Beach | 0.22 | 0.82 | 0.92 |
| Jensen Beach - Manatee Pocket | -0.21 | 0.83 | 0.92 |
| South Fork - Vero Beach | 0.21 | 0.84 | 0.92 |
| Jupiter Narrows - North Fork | -0.20 | 0.84 | 0.92 |
| Linkport - Vero Beach | -0.19 | 0.85 | 0.93 |
| Middle Estuary - Sebastian Inlet | 0.18 | 0.85 | 0.92 |
| Fort Pierce - Vero Beach | -0.17 | 0.87 | 0.93 |
| North Fork - Vero Beach | 0.15 | 0.88 | 0.94 |
| Jupiter Narrows - Linkport | 0.13 | 0.89 | 0.95 |
| Round Island - Vero Beach Marina | -0.13 | 0.89 | 0.94 |
| Fort Pierce - Jupiter Narrows | -0.12 | 0.91 | 0.95 |
| Harbor Branch Channel - Middle Estuary | 0.10 | 0.92 | 0.96 |
| Sebastian Inlet - South Fork 2 | 0.073 | 0.94 | 0.98 |
| Hobe Sound - Sebastian Inlet | -0.059 | 0.95 | 0.98 |
| North Fork - South Fork | -0.055 | 0.96 | 0.98 |
| Jupiter Narrows - Vero Beach | -0.052 | 0.96 | 0.98 |
| Harbortown Marina - Manatee Pocket | 0.051 | 0.96 | 0.97 |
| Fort Pierce - Linkport | 0.017 | 0.99 | 0.99 |
| Hobe Sound - South Fork 2 | 0.014 | 0.99 | 0.99 |
| **Location** | **15** | **0.0055** | **0.0083** |
| North IRL^f^- South Central IRL | -3.2 | 0.0012 | 0.0062 |
| North IRL- SLE | -3.4 | 0.0008 | 0.0077 |
| North IRL- South IRL | -2.4 | 0.014 | 0.048 |
| North IRL- North Central IRL | -1.5 | 0.14 | 0.24 |
| North Central IRL – SLE^g^ | -1.5 | 0.12 | 0.24 |
| North Central IRL - South Central IRL | -1.5 | 0.12 | 0.30 |
| North Central IRL - South IRL | -0.86 | 0.39 | 0.56 |
| SLE - South IRL | 0.63 | 0.53 | 0.59 |
| South IRL - South Central IRL | -0.69 | 0.49 | 0.61 |
| SLE - South Central IRL | -0.12 | 0.90 | 0.90 |
| **Mann Whitney U/Kruskal-Wallis Overall Test Category** | **W*/chi²** | p value | BH-adjusted  p value |
| Dunn Pair-wise test category | Z |  |  |
| **Overall Sampling Season** | **106** | **< 2.2E-16** | **<1.1E-15** |
| Mar/Apr 2017 - Apr 2018 | 1.3 | 0.19 | 0.19 |
| Mar/Apr 2017 - Aug/Sept 2016 | -3.5 | 0.00051 | 0.00062 |
| Apr 2018 - Aug/Sept 2016 | -5.0 | 6.5E-07 | 9.7E-07 |
| Mar/Apr 2017 - Oct/Nov 2017 | 6.4 | 1.7E-10 | 5.1E-10 |
| Apr 2018 - Oct/Nov 2017 | 5.4 | 6.2E-08 | 1.2E-07 |
| Aug/Sept 2016 - Oct/Nov 2017 | 10 | 8.4E-24 | 5.0E-23 |
| **IRL Sampling Season** | **73** | **1.0E-15** | **3.8E-15** |
| IRL Mar/Apr 2017 - IRL Apr 2018 | 1.4 | 0.16 | 0.16 |
| IRL Mar/Apr 2017 - IRL Aug/Sept 2016 | -2.5 | 0.013 | 0.015 |
| IRL Apr 2018 - IRL Aug/Sept 2016 | -4.1 | 4.3E-05 | 6.5E-05 |
| IRL Mar/Apr 2017 - IRL Oct/Nov 2017 | 5.6 | 2.8E-08 | 8.4E-08 |
| IRL Apr 2018 - IRL Oct/Nov 2017 | 4.5 | 6.8E-06 | 1.4E-05 |
| IRL Aug/Sept 2016 - IRL Oct/Nov 2017 | 8.2 | 1.9E-16 | 1.1E-15 |
| **SLE Sampling Season** | **34** | **2.0E-07** | **3.7E-07** |
| SLE Mar/Apr 2017 - SLE Apr 2018 | 0.087 | 0.93 | 0.93 |
| SLE Mar/Apr 2017 - SLE Aug/Sept 2016 | -2.9 | 0.00338 | 0.00677 |
| SLE Apr 2018 - SLE Aug/Sept 2016 | -3.0 | 0.00254 | 0.00763 |
| SLE Mar/Apr 2017 - SLE Oct/Nov 2017 | 2.9 | 0.00371 | 0.00557 |
| SLE Apr 2018 - SLE Oct/Nov 2017 | 2.8 | 0.00489 | 0.00587 |
| SLE Aug/Sept 2016 - SLE Oct/Nov 2017 | 5.8 | 5.5E-09 | 3.3E-08 |

^a^Bold text is associated with testing the overall differences within a category with Kruskal-Wallis or Mann-Whitney use with a * indicating the latter was used. ^b^Regular text is associated with pair-wise Dunn testing. ^c^BH stands for Benjami-Hochberg, ^d^TOM for total organic matter, ^e^Cu for copper, ^f^IRL for Indian River Lagoon and ^g^SLE for St. Lucie Estuary.
