# Supplementary material for: Defining the sediment prokaryotic communities of the Indian River Lagoon, FL, USA, an Estuary of National Significance
Source: PLoS One. 2020 Oct 26;15(10):e0236305. doi: 10.1371/journal.pone.0236305 (PMC7588086; doi:10.1371/journal.pone.0236305)
Supplement: S7 Table — aBold text is associated with testing the overall differences within a category and bregular text is associated with pair-wise testing. cP(perms) stands for permutational p value, P(MC) for Monte-Carlo p value, eTOM for total organic matter, fCu for copper, gIRL stands for Indian River Lagoon and hSLE for St. Lucie Estuary. (DOCX) [file pone.0236305.s012.docx]

S7 Table: Full results from the permutational analysis of variance tests.

| **Overall Parameter Category^a^** | **Pseudo-F** | P(perms)^c^ | P(MC)^d^ |
| --- | --- | --- | --- |
| Pair-wise test category^b^ | t statistic |  |  |
| **Estuary** | **42** | **0.0001** | **0.0001^a^** |
| **TOM^e^/Cu^f^** | **7.2** | **0.0001** | **0.0001** |
| High TOM/High Cu - High TOM/Low Cu | 2.7 | 0.0001 | 0.0001 |
| High TOM/High Cu - Low TOM/High Cu | 1.2 | 0.050 | 0.072 |
| High TOM/Low Cu - Low TOM/High Cu | 3.7 | 0.0001 | 0.0001 |
| High TOM/High Cu - Low TOM/Low Cu | 1.1 | 0.47 | 0.32 |
| High TOM/Low Cu - Low TOM/Low Cu | 2.8 | 0.0001 | 0.0001 |
| Low TOM/High Cu - Low TOM/Low Cu | 1.4 | 0.034 | 0.061 |
| **Muck Characteristics** | **5.4** | **0.0001** | **0.0001** |
| 3 - 1 | 1.9 | 0.0002 | 0.0001 |
| 3 - 2 | 3.4 | 0.0001 | 0.0001 |
| 1 - 2 | 1.6 | 0.0028 | 0.0022 |
| 3 - 0 | 1.4 | 0.030 | 0.034 |
| 1 - 0 | 0.8 | 0.88 | 0.76 |
| 2 - 0 | 1.5 | 0.013 | 0.017 |
| **Estuary by Season** | **17** | **0.0001** | **0.0001** |
| IRL^f^ Dry, IRL Wet | 1.8 | 0.0001 | 0.0001 |
| IRL Dry, SLE^g^ Dry | 5.0 | 0.0001 | 0.0001 |
| IRL Dry, SLE Wet | 5.2 | 0.0001 | 0.0001 |
| IRL Wet, SLE Dry | 4.6 | 0.0001 | 0.0001 |
| IRL Wet, SLE Wet | 4.7 | 0.0001 | 0.0001 |
| SLE Dry, SLE Wet | 2.1 | 0.0001 | 0.0001 |
| **Location** | **10.05** | **0.0001** | **0.0001** |
| North Central IRL, South Central IRL | 2.7 | 0.0001 | 0.0001 |
| North Central IRL, South IRL | 2.9 | 0.0001 | 0.0001 |
| North Central IRL, North IRL | 2.9 | 0.0001 | 0.0001 |
| North Central IRL, SLE | 5.6 | 0.0001 | 0.0001 |
| South Central IRL, South IRL | 3.0 | 0.0001 | 0.0001 |
| South Central IRL, North IRL | 2.6 | 0.0001 | 0.0001 |
| South Central IRL, SLE | 5.3 | 0.0001 | 0.0001 |
| South IRL, North IRL | 3.6 | 0.0001 | 0.0001 |
| South IRL, SLE | 5.6 | 0.0001 | 0.0001 |
| North IRL, SLE | 5.3 | 0.0001 | 0.0001 |
| **Estuary by Sampling** | **10** | **0.0001** | **0.0001** |
| IRL Apr 2018, IRL Oct/Nov 2017 | 2.0 | 0.0001 | 0.0001 |
| IRL Apr 2018, IRL Mar/Apr 2017 | 1.4 | 0.0049 | 0.0067 |
| IRL Apr 2018, IRL Aug/Sept 2016 | 2.4 | 0.0001 | 0.0001 |
| **Overall Parameter Category** | **Pseudo-F** | P(perms) | P(MC) |
| Pair-wise test category | t statistic |  |  |
| **Estuary by Sampling** | | | |
| IRL Apr 2018, SLE Mar/Apr 2017 | 3.6 | 0.0001 | 0.0001 |
| IRL Apr 2018, SLE Apr 2018 | 3.6 | 0.0001 | 0.0001 |
| IRL Apr 2018, SLE Aug/Sept 2016 | 4.1 | 0.0001 | 0.0001 |
| IRL Apr 2018, SLE Oct/Nov 2017 | 3.9 | 0.0001 | 0.0001 |
| IRL Oct/Nov 2017, IRL Mar/Apr 2017 | 2.2 | 0.0001 | 0.0001 |
| IRL Oct/Nov 2017, IRL Aug/Sept 2016 | 2.9 | 0.0001 | 0.0001 |
| IRL Oct/Nov 2017, SLE Mar/Apr 2017 | 3.4 | 0.0001 | 0.0001 |
| IRL Oct/Nov 2017, SLE Apr 2018 | 3.4 | 0.0001 | 0.0001 |
| IRL Oct/Nov 2017, SLE Aug/Sept 2016 | 3.9 | 0.0001 | 0.0001 |
| IRL Oct/Nov 2017, SLE Oct/Nov 2017 | 3.6 | 0.0001 | 0.0001 |
| IRL Mar/Apr 2017, IRL Aug/Sept 2016 | 1.8 | 0.0004 | 0.001 |
| IRL Mar/Apr 2017, SLE Mar/Apr 2017 | 3.6 | 0.0001 | 0.0001 |
| IRL Mar/Apr 2017, SLE Apr 2018 | 3.7 | 0.0001 | 0.0001 |
| IRL Mar/Apr 2017, SLE Aug/Sept 2016 | 4.2 | 0.0001 | 0.0001 |
| IRL Mar/Apr 2017, SLE Oct/Nov 2017 | 4.0 | 0.0001 | 0.0001 |
| IRL Aug/Sept 2016, SLE Mar/Apr 2017 | 3.7 | 0.0001 | 0.0001 |
| IRL Aug/Sept 2016, SLE Apr 2018 | 3.8 | 0.0001 | 0.0001 |
| IRL Aug/Sept 2016, SLE Aug/Sept 2016 | 4.1 | 0.0001 | 0.0001 |
| IRL Aug/Sept 2016, SLE Oct/Nov 2017 | 3.9 | 0.0001 | 0.0001 |
| SLE Mar/Apr 2017, SLE Apr 2018 | 1.1 | 0.25 | 0.29 |
| SLE Mar/Apr 2017, SLE Aug/Sept 2016 | 1.7 | 0.011 | 0.017 |
| SLE Mar/Apr 2017, SLE Oct/Nov 2017 | 2.2 | 0.0001 | 0.0005 |
| SLE Apr 2018, SLE Aug/Sept 2016 | 1.9 | 0.0022 | 0.0049 |
| SLE Apr 2018, SLE Oct/Nov 2017 | 2.0 | 0.0001 | 0.0011 |
| SLE Aug/Sept 2016, SLE Oct/Nov 2017 | 2.2 | 0.0003 | 0.0007 |
| **Site** | **11** | **0.0001** | **0.0001** |
| Barber Bridge, Fort Pierce | 2.7 | 0.0001 | 0.0001 |
| Barber Bridge, Harbor Branch Channel | 2.7 | 0.0001 | 0.0001 |
| Barber Bridge, Hobe Sound | 2.3 | 0.0002 | 0.0002 |
| Barber Bridge, Harbortown Marina | 2.7 | 0.0015 | 0.0004 |
| Barber Bridge, Jensen Beach | 2.2 | 0.0001 | 0.0001 |
| Barber Bridge, Jupiter Narrows | 2.2 | 0.0001 | 0.0001 |
| Barber Bridge, Linkport | 1.8 | 0.0002 | 0.0022 |
| Barber Bridge, Melbourne Causeway | 2.6 | 0.0001 | 0.0002 |
| Barber Bridge, Middle Estuary | 2.7 | 0.0001 | 0.0001 |
| Barber Bridge, Merritt Island Causeway | 2.8 | 0.0001 | 0.0001 |
| Barber Bridge, Manatee Pocket | 2.9 | 0.0001 | 0.0001 |
| Barber Bridge, North Fork | 3.2 | 0.0001 | 0.0001 |
| Barber Bridge, Round Island | 1.4 | 0.0054 | 0.0463 |
| **Overall Parameter Category** | **Pseudo-F** | P(perms) | P(MC) |
| Pair-wise test category | t statistic |  |  |
| **Site** | | | |
| Barber Bridge, South Fork | 3.5 | 0.0001 | 0.0001 |
| Barber Bridge, Sebastian Inlet | 2.1 | 0.0001 | 0.0001 |
| Barber Bridge, South Fork 2 | 3.5 | 0.0004 | 0.0001 |
| Barber Bridge, Vero Beach | 1.7 | 0.0031 | 0.0098 |
| Barber Bridge, Vero Beach Marina | 1.0 | 0.31 | 0.40 |
| Fort Pierce, Harbor Branch Channel | 3.8 | 0.0001 | 0.0001 |
| Fort Pierce, Hobe Sound | 2.7 | 0.0001 | 0.0001 |
| Fort Pierce, Harbortown Marina | 3.0 | 0.0001 | 0.0001 |
| Fort Pierce, Jensen Beach | 2.8 | 0.0001 | 0.0001 |
| Fort Pierce, Jupiter Narrows | 2.9 | 0.0001 | 0.0001 |
| Fort Pierce, Linkport | 2.7 | 0.0002 | 0.0001 |
| Fort Pierce, Melbourne Causeway | 4.1 | 0.0001 | 0.0001 |
| Fort Pierce, Middle Estuary | 3.7 | 0.0001 | 0.0001 |
| Fort Pierce, Merritt Island Causeway | 3.7 | 0.0001 | 0.0001 |
| Fort Pierce, Manatee Pocket | 4.0 | 0.0001 | 0.0001 |
| Fort Pierce, North Fork | 4.3 | 0.0001 | 0.0001 |
| Fort Pierce, Round Island | 2.4 | 0.0001 | 0.0002 |
| Fort Pierce, South Fork | 4.5 | 0.0001 | 0.0001 |
| Fort Pierce, Sebastian Inlet | 2.4 | 0.0001 | 0.0001 |
| Fort Pierce, South Fork 2 | 4.5 | 0.0001 | 0.0001 |
| Fort Pierce, Vero Beach | 2.5 | 0.0001 | 0.0001 |
| Fort Pierce, Vero Beach Marina | 2.7 | 0.0001 | 0.0002 |
| Harbor Branch Channel, Hobe Sound | 3.1 | 0.0001 | 0.0001 |
| Harbor Branch Channel, Harbortown Marina | 2.7 | 0.0001 | 0.0001 |
| Harbor Branch Channel, Jensen Beach | 3.7 | 0.0001 | 0.0001 |
| Harbor Branch Channel, Jupiter Narrows | 3.4 | 0.0001 | 0.0001 |
| Harbor Branch Channel, Linkport | 2.7 | 0.0001 | 0.0001 |
| Harbor Branch Channel, Melbourne Causeway | 3.1 | 0.0001 | 0.0001 |
| Harbor Branch Channel, Middle Estuary | 3.6 | 0.0001 | 0.0001 |
| Harbor Branch Channel, Merritt Island Causeway | 3.3 | 0.0001 | 0.0001 |
| Harbor Branch Channel, Manatee Pocket | 3.7 | 0.0001 | 0.0001 |
| Harbor Branch Channel, North Fork | 4.2 | 0.0001 | 0.0001 |
| Harbor Branch Channel, Round Island | 2.6 | 0.0002 | 0.0002 |
| Harbor Branch Channel, South Fork | 4.3 | 0.0001 | 0.0001 |
| Harbor Branch Channel, Sebastian Inlet | 3.1 | 0.0001 | 0.0001 |
| Harbor Branch Channel, South Fork 2 | 4.3 | 0.0001 | 0.0001 |
| Harbor Branch Channel, Vero Beach | 3.4 | 0.0001 | 0.0001 |
| Harbor Branch Channel, Vero Beach Marina | 2.6 | 0.0001 | 0.0001 |
| Hobe Sound, Harbortown Marina | 2.9 | 0.0001 | 0.0001 |
| Hobe Sound, Jensen Beach | 2.5 | 0.0001 | 0.0001 |
| **Overall Parameter Category** | **Pseudo-F** | P(perms) | P(MC) |
| Pair-wise test category | t statistic |  |  |
| **Site** | | | |
| Hobe Sound, Jupiter Narrows | 2.2 | 0.0001 | 0.0001 |
| Hobe Sound, Linkport | 2.1 | 0.0001 | 0.0001 |
| Hobe Sound, Melbourne Causeway | 3.6 | 0.0001 | 0.0001 |
| Hobe Sound, Middle Estuary | 3.5 | 0.0001 | 0.0001 |
| Hobe Sound, Merritt Island Causeway | 3.3 | 0.0001 | 0.0001 |
| Hobe Sound, Manatee Pocket | 3.4 | 0.0001 | 0.0001 |
| Hobe Sound, North Fork | 4.1 | 0.0001 | 0.0001 |
| Hobe Sound, Round Island | 2.0 | 0.0001 | 0.001 |
| Hobe Sound, South Fork | 4.3 | 0.0001 | 0.0001 |
| Hobe Sound, Sebastian Inlet | 2.3 | 0.0001 | 0.0001 |
| Hobe Sound, South Fork 2 | 4.3 | 0.0001 | 0.0001 |
| Hobe Sound, Vero Beach | 2.4 | 0.0001 | 0.0001 |
| Hobe Sound, Vero Beach Marina | 2.2 | 0.0002 | 0.0001 |
| Harbortown Marina, Jensen Beach | 3.1 | 0.0002 | 0.0001 |
| Harbortown Marina, Jupiter Narrows | 3.0 | 0.0001 | 0.0001 |
| Harbortown Marina, Linkport | 2.8 | 0.0001 | 0.0001 |
| Harbortown Marina, Melbourne Causeway | 3.1 | 0.0001 | 0.0001 |
| Harbortown Marina, Middle Estuary | 2.6 | 0.0001 | 0.0001 |
| Harbortown Marina, Merritt Island Causeway | 3.1 | 0.0001 | 0.0001 |
| Harbortown Marina, Manatee Pocket | 3.3 | 0.0002 | 0.0001 |
| Harbortown Marina, North Fork | 3.2 | 0.0001 | 0.0001 |
| Harbortown Marina, Round Island | 2.5 | 0.0016 | 0.0003 |
| Harbortown Marina, South Fork | 2.9 | 0.0002 | 0.0001 |
| Harbortown Marina, Sebastian Inlet | 2.8 | 0.0002 | 0.0003 |
| Harbortown Marina, South Fork 2 | 2.7 | 0.0001 | 0.0001 |
| Harbortown Marina, Vero Beach | 3.1 | 0.0002 | 0.0001 |
| Harbortown Marina, Vero Beach Marina | 2.6 | 0.0018 | 0.0006 |
| Jensen Beach, Jupiter Narrows | 2.4 | 0.0001 | 0.0001 |
| Jensen Beach, Linkport | 2.6 | 0.0001 | 0.0001 |
| Jensen Beach, Melbourne Causeway | 3.8 | 0.0001 | 0.0001 |
| Jensen Beach, Middle Estuary | 3.3 | 0.0001 | 0.0001 |
| Jensen Beach, Merritt Island Causeway | 3.9 | 0.0001 | 0.0001 |
| Jensen Beach, Manatee Pocket | 3.2 | 0.0001 | 0.0001 |
| Jensen Beach, North Fork | 3.9 | 0.0001 | 0.0001 |
| Jensen Beach, Round Island | 2.3 | 0.0001 | 0.0001 |
| Jensen Beach, South Fork | 4.3 | 0.0001 | 0.0001 |
| Jensen Beach, Sebastian Inlet | 2.5 | 0.0001 | 0.0001 |
| Jensen Beach, South Fork 2 | 4.5 | 0.0001 | 0.0001 |
| Jensen Beach, Vero Beach | 2.2 | 0.0001 | 0.0001 |
| Jensen Beach, Vero Beach Marina | 2.1 | 0.0002 | 0.0006 |
| **Overall Parameter Category** | **Pseudo-F** | P(perms) | P(MC) |
| Pair-wise test category | t statistic |  |  |
| **Site** | | | |
| Jupiter Narrows, Linkport | 2.6 | 0.0001 | 0.0002 |
| Jupiter Narrows, Melbourne Causeway | 3.7 | 0.0001 | 0.0001 |
| Jupiter Narrows, Middle Estuary | 3.2 | 0.0001 | 0.0001 |
| Jupiter Narrows, Merritt Island Causeway | 3.6 | 0.0001 | 0.0001 |
| Jupiter Narrows, Manatee Pocket | 2.9 | 0.0001 | 0.0001 |
| Jupiter Narrows, North Fork | 3.8 | 0.0001 | 0.0001 |
| Jupiter Narrows, Round Island | 2.2 | 0.0003 | 0.0001 |
| Jupiter Narrows, South Fork | 4.1 | 0.0001 | 0.0001 |
| Jupiter Narrows, Sebastian Inlet | 2.7 | 0.0001 | 0.0001 |
| Jupiter Narrows, South Fork 2 | 4.3 | 0.0001 | 0.0001 |
| Jupiter Narrows, Vero Beach | 2.5 | 0.0001 | 0.0001 |
| Jupiter Narrows, Vero Beach Marina | 2.1 | 0.0001 | 0.0002 |
| Linkport, Melbourne Causeway | 2.9 | 0.0001 | 0.0001 |
| Linkport, Middle Estuary | 3.5 | 0.0001 | 0.0001 |
| Linkport, Merritt Island Causeway | 2.9 | 0.0001 | 0.0001 |
| Linkport, Manatee Pocket | 3.6 | 0.0001 | 0.0001 |
| Linkport, North Fork | 4.1 | 0.0001 | 0.0001 |
| Linkport, Round Island | 1.5 | 0.0041 | 0.0215 |
| Linkport, South Fork | 4.4 | 0.0001 | 0.0001 |
| Linkport, Sebastian Inlet | 2.0 | 0.0001 | 0.0004 |
| Linkport, South Fork 2 | 4.3 | 0.0001 | 0.0001 |
| Linkport, Vero Beach | 1.9 | 0.0002 | 0.0006 |
| Linkport, Vero Beach Marina | 1.8 | 0.0001 | 0.0024 |
| Melbourne Causeway, Middle Estuary | 3.6 | 0.0001 | 0.0001 |
| Melbourne Causeway, Merritt Island Causeway | 3.1 | 0.0001 | 0.0001 |
| Melbourne Causeway, Manatee Pocket | 3.9 | 0.0001 | 0.0001 |
| Melbourne Causeway, North Fork | 4.2 | 0.0001 | 0.0001 |
| Melbourne Causeway, Round Island | 2.7 | 0.0001 | 0.0001 |
| Melbourne Causeway, South Fork | 4.3 | 0.0001 | 0.0001 |
| Melbourne Causeway, Sebastian Inlet | 3.4 | 0.0001 | 0.0001 |
| Melbourne Causeway, South Fork 2 | 4.4 | 0.0001 | 0.0001 |
| Melbourne Causeway, Vero Beach | 3.4 | 0.0001 | 0.0001 |
| Melbourne Causeway, Vero Beach Marina | 2.5 | 0.0001 | 0.0001 |
| Middle Estuary, Merritt Island Causeway | 3.7 | 0.0001 | 0.0001 |
| Middle Estuary, Manatee Pocket | 3.0 | 0.0001 | 0.0001 |
| Middle Estuary, North Fork | 1.9 | 0.0002 | 0.002 |
| Middle Estuary, Round Island | 2.8 | 0.0001 | 0.0001 |
| Middle Estuary, South Fork | 1.7 | 0.015 | 0.018 |
| Middle Estuary, Sebastian Inlet | 3.4 | 0.0001 | 0.0001 |
| Middle Estuary, South Fork 2 | 2.5 | 0.0001 | 0.0001 |
| **Overall Parameter Category** | **Pseudo-F** | P(perms) | P(MC) |
| Pair-wise test category | t statistic |  |  |
| **Site** | | | |
| Middle Estuary, Vero Beach | 3.5 | 0.0001 | 0.0001 |
| Middle Estuary, Vero Beach Marina | 2.6 | 0.0001 | 0.0001 |
| Merritt Island Causeway, Manatee Pocket | 4.1 | 0.0001 | 0.0001 |
| Merritt Island Causeway, North Fork | 4.2 | 0.0001 | 0.0001 |
| Merritt Island Causeway, Round Island | 2.5 | 0.0002 | 0.0003 |
| Merritt Island Causeway, South Fork | 4.3 | 0.0001 | 0.0001 |
| Merritt Island Causeway, Sebastian Inlet | 3.1 | 0.0001 | 0.0001 |
| Merritt Island Causeway, South Fork 2 | 4.3 | 0.0001 | 0.0001 |
| Merritt Island Causeway, Vero Beach | 3.4 | 0.0001 | 0.0001 |
| Merritt Island Causeway, Vero Beach Marina | 2.8 | 0.0003 | 0.0001 |
| Manatee Pocket, North Fork | 3.8 | 0.0001 | 0.0001 |
| Manatee Pocket, Round Island | 3.0 | 0.0002 | 0.0001 |
| Manatee Pocket, South Fork | 4.1 | 0.0001 | 0.0001 |
| Manatee Pocket, Sebastian Inlet | 3.4 | 0.0001 | 0.0001 |
| Manatee Pocket, South Fork 2 | 4.4 | 0.0001 | 0.0001 |
| Manatee Pocket, Vero Beach | 3.6 | 0.0001 | 0.0001 |
| Manatee Pocket, Vero Beach Marina | 2.7 | 0.0001 | 0.0001 |
| North Fork, Round Island | 3.3 | 0.0001 | 0.0001 |
| North Fork, South Fork | 2.6 | 0.0001 | 0.0001 |
| North Fork, Sebastian Inlet | 4.1 | 0.0001 | 0.0001 |
| North Fork, South Fork 2 | 3.2 | 0.0001 | 0.0001 |
| North Fork, Vero Beach | 4.0 | 0.0001 | 0.0001 |
| North Fork, Vero Beach Marina | 3.2 | 0.0001 | 0.0001 |
| Round Island, South Fork | 3.5 | 0.0002 | 0.0001 |
| Round Island, Sebastian Inlet | 1.8 | 0.0007 | 0.0031 |
| Round Island, South Fork 2 | 3.5 | 0.0001 | 0.0001 |
| Round Island, Vero Beach | 1.7 | 0.0054 | 0.013 |
| Round Island, Vero Beach Marina | 1.5 | 0.0088 | 0.045 |
| South Fork, Sebastian Inlet | 4.3 | 0.0001 | 0.0001 |
| South Fork, South Fork 2 | 1.9 | 0.0002 | 0.0008 |
| South Fork, Vero Beach | 4.3 | 0.0001 | 0.0001 |
| South Fork, Vero Beach Marina | 3.5 | 0.0002 | 0.0001 |
| Sebastian Inlet, South Fork 2 | 4.3 | 0.0001 | 0.0001 |
| Sebastian Inlet, Vero Beach | 2.2 | 0.0001 | 0.0001 |
| Sebastian Inlet, Vero Beach Marina | 2.0 | 0.0002 | 0.0012 |
| South Fork 2, Vero Beach | 4.4 | 0.0001 | 0.0001 |
| South Fork 2, Vero Beach Marina | 3.5 | 0.0001 | 0.0001 |
| Vero Beach, Vero Beach Marina | 1.7 | 0.0055 | 0.0086 |

| **Overall Parameter Category^a^** | | **Pseudo-F** | | P(perms)^c^ | | P(MC)^d^ | |
| --- | --- | --- | --- | --- | --- | --- | --- |
| Pair-wise test category^b^ | | t statistic | |  |  |  |  |
| **Aug/Sept 2016 by Site** | | **12.957** | | **0.0001** | | **0.0001** | |
| Fort Pierce, Harbor Branch Channel | | 3.4225 | | 0.1055 | | 0.0041 | |
| Fort Pierce, Hobe Sound NWR | | 2.7316 | | 0.0979 | | 0.0106 | |
| Fort Pierce, Jensen Beach | | 2.8805 | | 0.1045 | | 0.0061 | |
| Fort Pierce, Jupiter Narrows | | 2.8065 | | 0.0945 | | 0.0078 | |
| Fort Pierce, Linkport | | 2.4549 | | 0.0986 | | 0.0146 | |
| Fort Pierce, Melbourne Causeway | | 4.0605 | | 0.1002 | | 0.002 | |
| Fort Pierce, Middle Estuary | | 4.2741 | | 0.1009 | | 0.0016 | |
| Fort Pierce, Merritt Island Causeway | | 4.0153 | | 0.1007 | | 0.0021 | |
| Fort Pierce, Manatee Pocket | | 3.6181 | | 0.1005 | | 0.0033 | |
| Fort Pierce, North Fork | | 4.4585 | | 0.0972 | | 0.0025 | |
| Fort Pierce, South Fork | | 4.5035 | | 0.099 | | 0.0012 | |
| Fort Pierce, Sebastian Inlet | | 2.392 | | 0.1015 | | 0.0121 | |
| Fort Pierce, South Fork 2 | | 4.4071 | | 0.1017 | | 0.0018 | |
| Fort Pierce, Vero Beach | | 2.4763 | | 0.1015 | | 0.0111 | |
| Harbor Branch Channel, Hobe Sound NWR | | 2.9262 | | 0.0946 | | 0.0077 | |
| Harbor Branch Channel, Jensen Beach | | 3.3534 | | 0.1022 | | 0.005 | |
| Harbor Branch Channel, Jupiter Narrows | | 2.9444 | | 0.0952 | | 0.0066 | |
| Harbor Branch Channel, Linkport | | 2.7703 | | 0.0985 | | 0.0092 | |
| Harbor Branch Channel, Melbourne Causeway | | 2.9759 | | 0.102 | | 0.0055 | |
| Harbor Branch Channel, Middle Estuary | | 3.9253 | | 0.1023 | | 0.0023 | |
| Harbor Branch Channel, Merritt Island Causeway | | 3.3044 | | 0.1003 | | 0.0046 | |
| Harbor Branch Channel, Manatee Pocket | | 3.1206 | | 0.0982 | | 0.0074 | |
| Harbor Branch Channel, North Fork | | 4.1815 | | 0.1037 | | 0.0015 | |
| Harbor Branch Channel, South Fork | | 4.1452 | | 0.0992 | | 0.0021 | |
| Harbor Branch Channel, Sebastian Inlet | | 2.8821 | | 0.1021 | | 0.0086 | |
| Harbor Branch Channel, South Fork 2 | | 4.2048 | | 0.0959 | | 0.0021 | |
| Harbor Branch Channel, Vero Beach | | 3.2762 | | 0.1049 | | 0.0051 | |
| Hobe Sound NWR, Jensen Beach | | 2.7124 | | 0.1023 | | 0.0111 | |
| Hobe Sound NWR, Jupiter Narrows | | 2.1871 | | 0.0995 | | 0.021 | |
| Hobe Sound NWR, Linkport | | 2.2296 | | 0.1038 | | 0.0147 | |
| Hobe Sound NWR, Melbourne Causeway | | 3.5134 | | 0.1029 | | 0.0041 | |
| Hobe Sound NWR, Middle Estuary | | 4.0042 | | 0.1016 | | 0.0031 | |
| Hobe Sound NWR, Merritt Island Causeway | | 3.5318 | | 0.0984 | | 0.0035 | |
| Hobe Sound NWR, Manatee Pocket | | 3.1459 | | 0.0989 | | 0.006 | |
| Hobe Sound NWR, North Fork | | 4.2929 | | 0.1002 | | 0.0022 | |
| Hobe Sound NWR, South Fork | | 4.3481 | | 0.099 | | 0.0011 | |
| Hobe Sound NWR, Sebastian Inlet | | 2.2688 | | 0.0943 | | 0.0182 | |
| Hobe Sound NWR, South Fork 2 | | 4.3337 | | 0.098 | | 0.0024 | |
| Hobe Sound NWR, Vero Beach | | 2.3793 | | 0.1013 | | 0.0153 | |
| **Overall Parameter Category** | **Pseudo-F** | | P(perms) | | P(MC) | |  |
| Pair-wise test category | t statistic | |  |  |  |  |  |
| **Aug/Sept 2016 by Site** | | | | | | |  |
| Jensen Beach, Jupiter Narrows | | 2.387 | | 0.0991 | | 0.0131 | |
| Jensen Beach, Linkport | | 2.5965 | | 0.1027 | | 0.0098 | |
| Jensen Beach, Melbourne Causeway | | 3.7228 | | 0.1001 | | 0.0031 | |
| Jensen Beach, Middle Estuary | | 3.9576 | | 0.0986 | | 0.0027 | |
| Jensen Beach, Merritt Island Causeway | | 4.1502 | | 0.0984 | | 0.0017 | |
| Jensen Beach, Manatee Pocket | | 2.9498 | | 0.098 | | 0.0056 | |
| Jensen Beach, North Fork | | 4.435 | | 0.0994 | | 0.0026 | |
| Jensen Beach, South Fork | | 4.5482 | | 0.1004 | | 0.0012 | |
| Jensen Beach, Sebastian Inlet | | 2.5899 | | 0.1093 | | 0.0109 | |
| Jensen Beach, South Fork 2 | | 4.5946 | | 0.0946 | | 0.0016 | |
| Jensen Beach, Vero Beach | | 2.5052 | | 0.1009 | | 0.0116 | |
| Jupiter Narrows, Linkport | | 2.3199 | | 0.104 | | 0.0161 | |
| Jupiter Narrows, Melbourne Causeway | | 3.3587 | | 0.0996 | | 0.0054 | |
| Jupiter Narrows, Middle Estuary | | 3.5961 | | 0.1042 | | 0.0023 | |
| Jupiter Narrows, Merritt Island Causeway | | 3.5968 | | 0.0986 | | 0.004 | |
| Jupiter Narrows, Manatee Pocket | | 2.5468 | | 0.1014 | | 0.0111 | |
| Jupiter Narrows, North Fork | | 3.9748 | | 0.1015 | | 0.0029 | |
| Jupiter Narrows, South Fork | | 4.0693 | | 0.0967 | | 0.0023 | |
| Jupiter Narrows, Sebastian Inlet | | 2.5122 | | 0.101 | | 0.0106 | |
| Jupiter Narrows, South Fork 2 | | 4.1591 | | 0.1064 | | 0.0018 | |
| Jupiter Narrows, Vero Beach | | 2.5995 | | 0.1035 | | 0.0106 | |
| Linkport, Melbourne Causeway | | 3.3415 | | 0.1022 | | 0.004 | |
| Linkport, Middle Estuary | | 4.007 | | 0.1039 | | 0.0021 | |
| Linkport, Merritt Island Causeway | | 3.2604 | | 0.1045 | | 0.0045 | |
| Linkport, Manatee Pocket | | 3.1213 | | 0.1019 | | 0.0063 | |
| Linkport, North Fork | | 4.313 | | 0.0977 | | 0.0017 | |
| Linkport, South Fork | | 4.3735 | | 0.1004 | | 0.0021 | |
| Linkport, Sebastian Inlet | | 1.9057 | | 0.1016 | | 0.0339 | |
| Linkport, South Fork 2 | | 4.3797 | | 0.0998 | | 0.0013 | |
| Linkport, Vero Beach | | 1.9547 | | 0.0974 | | 0.0318 | |
| Melbourne Causeway, Middle Estuary | | 4.1255 | | 0.1005 | | 0.0027 | |
| Melbourne Causeway, Merritt Island Causeway | | 3.8725 | | 0.0996 | | 0.0023 | |
| Melbourne Causeway, Manatee Pocket | | 3.4144 | | 0.1027 | | 0.0036 | |
| Melbourne Causeway, North Fork | | 4.5093 | | 0.0969 | | 0.0017 | |
| Melbourne Causeway, South Fork | | 4.6123 | | 0.1013 | | 0.0014 | |
| Melbourne Causeway, Sebastian Inlet | | 3.3816 | | 0.1066 | | 0.0053 | |
| Melbourne Causeway, South Fork 2 | | 4.7351 | | 0.0944 | | 0.0012 | |
| Melbourne Causeway, Vero Beach | | 3.6533 | | 0.1023 | | 0.0046 | |
| Middle Estuary, Merritt Island Causeway | | 4.6321 | | 0.0958 | | 0.002 | |
| **Overall Parameter Category** | **Pseudo-F** | | P(perms) | | P(MC) | |  |
| Pair-wise test category | t statistic | |  |  |  |  |  |
| **Aug/Sept 2016 by Site** | | | | | | |  |
| Middle Estuary, Manatee Pocket | | 3.315 | | 0.1011 | | 0.0047 | |
| Middle Estuary, North Fork | | 2.2311 | | 0.0969 | | 0.0192 | |
| Middle Estuary, South Fork | | 2.8869 | | 0.0965 | | 0.0079 | |
| Middle Estuary, Sebastian Inlet | | 3.9898 | | 0.1013 | | 0.0022 | |
| Middle Estuary, South Fork 2 | | 4.0098 | | 0.0979 | | 0.002 | |
| Middle Estuary, Vero Beach | | 4.1579 | | 0.0989 | | 0.0031 | |
| Merritt Island Causeway, Manatee Pocket | | 3.9923 | | 0.0988 | | 0.0023 | |
| Merritt Island Causeway, North Fork | | 4.8256 | | 0.1086 | | 0.0019 | |
| Merritt Island Causeway, South Fork | | 4.8509 | | 0.0999 | | 0.0013 | |
| Merritt Island Causeway, Sebastian Inlet | | 3.2927 | | 0.0998 | | 0.0046 | |
| Merritt Island Causeway, South Fork 2 | | 4.7956 | | 0.0981 | | 0.0013 | |
| Merritt Island Causeway, Vero Beach | | 3.8149 | | 0.1002 | | 0.0032 | |
| Manatee Pocket, North Fork | | 4.0564 | | 0.0978 | | 0.0022 | |
| Manatee Pocket, South Fork | | 4.0576 | | 0.1046 | | 0.002 | |
| Manatee Pocket, Sebastian Inlet | | 3.1079 | | 0.1057 | | 0.0051 | |
| Manatee Pocket, South Fork 2 | | 4.3606 | | 0.0955 | | 0.0019 | |
| Manatee Pocket, Vero Beach | | 3.3912 | | 0.0993 | | 0.004 | |
| North Fork, South Fork | | 2.8797 | | 0.1035 | | 0.0087 | |
| North Fork, Sebastian Inlet | | 4.2381 | | 0.0967 | | 0.0017 | |
| North Fork, South Fork 2 | | 3.8762 | | 0.0996 | | 0.0027 | |
| North Fork, Vero Beach | | 4.4692 | | 0.0986 | | 0.0024 | |
| South Fork, Sebastian Inlet | | 4.2857 | | 0.0935 | | 0.0029 | |
| South Fork, South Fork 2 | | 2.4223 | | 0.0987 | | 0.0127 | |
| South Fork, Vero Beach | | 4.5706 | | 0.0992 | | 0.0017 | |
| Sebastian Inlet, South Fork 2 | | 4.2692 | | 0.0969 | | 0.0011 | |
| Sebastian Inlet, Vero Beach | | 2.2184 | | 0.1004 | | 0.0207 | |
| South Fork 2, Vero Beach | | 4.5578 | | 0.1002 | | 0.0012 | |
| **Mar/Apr 2017 by Site** | | **8.5855** | | **0.0001** | | **0.0001** | |
| Fort Pierce, Harbor Branch Channel | | 3.1314 | | 0.0928 | | 0.0052 | |
| Fort Pierce, Hobe Sound NWR | | 2.2739 | | 0.0965 | | 0.0159 | |
| Fort Pierce, Jensen Beach | | 2.4306 | | 0.1042 | | 0.0128 | |
| Fort Pierce, Jupiter Narrows | | 2.4606 | | 0.1019 | | 0.0108 | |
| Fort Pierce, Linkport | | 1.9996 | | 0.1008 | | 0.0283 | |
| Fort Pierce, Manatee Pocket | | 2.854 | | 0.0998 | | 0.0083 | |
| Fort Pierce, Melbourne Causeway | | 3.7893 | | 0.0947 | | 0.0035 | |
| Fort Pierce, Merritt Island Causeway | | 3.3069 | | 0.0959 | | 0.0051 | |
| Fort Pierce, Middle Estuary | | 2.4666 | | 0.0984 | | 0.0124 | |
| Fort Pierce, North Fork | | 3.4881 | | 0.0996 | | 0.0033 | |
| Fort Pierce, Sebastian Inlet | | 2.12 | | 0.1036 | | 0.0216 | |
| **Overall Parameter Category** | **Pseudo-F** | | P(perms) | | P(MC) | |  |
| Pair-wise test category | t statistic | |  |  |  |  |  |
| **Mar/Apr 2017 by Site** | | | | | | |  |
| Fort Pierce, South Fork | | 3.7437 | | 0.0983 | | 0.0026 | |
| Fort Pierce, South Fork 2 | | 4.1875 | | 0.1035 | | 0.0025 | |
| Fort Pierce, Vero Beach | | 2.4789 | | 0.0972 | | 0.0133 | |
| Harbor Branch Channel, Hobe Sound NWR | | 2.7847 | | 0.0994 | | 0.0091 | |
| Harbor Branch Channel, Jensen Beach | | 3.1934 | | 0.1018 | | 0.0063 | |
| Harbor Branch Channel, Jupiter Narrows | | 2.7334 | | 0.0998 | | 0.0085 | |
| Harbor Branch Channel, Linkport | | 2.1051 | | 0.0985 | | 0.0246 | |
| Harbor Branch Channel, Manatee Pocket | | 2.8657 | | 0.1026 | | 0.0085 | |
| Harbor Branch Channel, Melbourne Causeway | | 3.2432 | | 0.1022 | | 0.0049 | |
| Harbor Branch Channel, Merritt Island Causeway | | 3.4822 | | 0.1021 | | 0.0042 | |
| Harbor Branch Channel, Middle Estuary | | 2.5252 | | 0.0952 | | 0.0126 | |
| Harbor Branch Channel, North Fork | | 3.6761 | | 0.0978 | | 0.0024 | |
| Harbor Branch Channel, Sebastian Inlet | | 2.6746 | | 0.1014 | | 0.0086 | |
| Harbor Branch Channel, South Fork | | 3.7731 | | 0.1041 | | 0.0027 | |
| Harbor Branch Channel, South Fork 2 | | 4.3075 | | 0.1005 | | 0.0017 | |
| Harbor Branch Channel, Vero Beach | | 3.2156 | | 0.0988 | | 0.0056 | |
| Hobe Sound NWR, Jensen Beach | | 2.1089 | | 0.0999 | | 0.021 | |
| Hobe Sound NWR, Jupiter Narrows | | 1.6686 | | 0.1007 | | 0.0638 | |
| Hobe Sound NWR, Linkport | | 1.6756 | | 0.0959 | | 0.0614 | |
| Hobe Sound NWR, Manatee Pocket | | 2.5292 | | 0.0986 | | 0.0101 | |
| Hobe Sound NWR, Melbourne Causeway | | 3.5401 | | 0.0997 | | 0.0032 | |
| Hobe Sound NWR, Merritt Island Causeway | | 3.0941 | | 0.0958 | | 0.0048 | |
| Hobe Sound NWR, Middle Estuary | | 2.3713 | | 0.0997 | | 0.015 | |
| Hobe Sound NWR, North Fork | | 3.4063 | | 0.098 | | 0.004 | |
| Hobe Sound NWR, Sebastian Inlet | | 1.981 | | 0.0974 | | 0.0292 | |
| Hobe Sound NWR, South Fork | | 3.6694 | | 0.1017 | | 0.003 | |
| Hobe Sound NWR, South Fork 2 | | 4.1739 | | 0.0994 | | 0.0015 | |
| Hobe Sound NWR, Vero Beach | | 2.3536 | | 0.098 | | 0.0151 | |
| Jensen Beach, Jupiter Narrows | | 2.0839 | | 0.1049 | | 0.0219 | |
| Jensen Beach, Linkport | | 1.8776 | | 0.1009 | | 0.0401 | |
| Jensen Beach, Manatee Pocket | | 2.5323 | | 0.1029 | | 0.0102 | |
| Jensen Beach, Melbourne Causeway | | 3.817 | | 0.0987 | | 0.003 | |
| Jensen Beach, Merritt Island Causeway | | 3.4969 | | 0.0987 | | 0.0045 | |
| Jensen Beach, Middle Estuary | | 2.3024 | | 0.1018 | | 0.019 | |
| Jensen Beach, North Fork | | 3.4632 | | 0.0974 | | 0.004 | |
| Jensen Beach, Sebastian Inlet | | 2.1889 | | 0.1055 | | 0.0196 | |
| Jensen Beach, South Fork | | 3.843 | | 0.104 | | 0.002 | |
| Jensen Beach, South Fork 2 | | 4.4888 | | 0.0972 | | 0.0018 | |
| Jensen Beach, Vero Beach | | 2.1705 | | 0.1028 | | 0.0203 | |
| **Overall Parameter Category** | **Pseudo-F** | | P(perms) | | P(MC) | |  |
| Pair-wise test category | t statistic | |  |  |  |  |  |
| **Mar/Apr 2017 by Site** | | | | | | |  |
| Jupiter Narrows, Linkport | | 1.7922 | | 0.096 | | 0.0475 | |
| Jupiter Narrows, Manatee Pocket | | 2.0483 | | 0.0947 | | 0.0231 | |
| Jupiter Narrows, Melbourne Causeway | | 3.409 | | 0.1029 | | 0.0047 | |
| Jupiter Narrows, Merritt Island Causeway | | 3.1739 | | 0.1 | | 0.0058 | |
| Jupiter Narrows, Middle Estuary | | 2.1449 | | 0.0979 | | 0.0254 | |
| Jupiter Narrows, North Fork | | 3.1232 | | 0.1027 | | 0.0055 | |
| Jupiter Narrows, Sebastian Inlet | | 2.1855 | | 0.0977 | | 0.0173 | |
| Jupiter Narrows, South Fork | | 3.4341 | | 0.0935 | | 0.0042 | |
| Jupiter Narrows, South Fork 2 | | 4.023 | | 0.0961 | | 0.0027 | |
| Jupiter Narrows, Vero Beach | | 2.3441 | | 0.0985 | | 0.0147 | |
| Linkport, Manatee Pocket | | 2.2835 | | 0.0978 | | 0.0175 | |
| Linkport, Melbourne Causeway | | 2.5799 | | 0.106 | | 0.0113 | |
| Linkport, Merritt Island Causeway | | 2.3203 | | 0.0986 | | 0.0165 | |
| Linkport, Middle Estuary | | 2.1435 | | 0.1008 | | 0.0239 | |
| Linkport, North Fork | | 2.9208 | | 0.1001 | | 0.0067 | |
| Linkport, Sebastian Inlet | | 1.527 | | 0.0972 | | 0.0917 | |
| Linkport, South Fork | | 3.1312 | | 0.0954 | | 0.0052 | |
| Linkport, South Fork 2 | | 3.5221 | | 0.1069 | | 0.004 | |
| Linkport, Vero Beach | | 1.665 | | 0.1047 | | 0.0646 | |
| Manatee Pocket, North Fork | | 2.9238 | | 0.0954 | | 0.0064 | |
| Manatee Pocket, Sebastian Inlet | | 2.5339 | | 0.0992 | | 0.0101 | |
| Manatee Pocket, South Fork | | 3.0913 | | 0.099 | | 0.0057 | |
| Manatee Pocket, South Fork 2 | | 3.894 | | 0.0987 | | 0.0031 | |
| Manatee Pocket, Vero Beach | | 2.8966 | | 0.0983 | | 0.0064 | |
| Melbourne Causeway, Manatee Pocket | | 3.3971 | | 0.1026 | | 0.0045 | |
| Melbourne Causeway, Merritt Island Causeway | | 3.5389 | | 0.1045 | | 0.0037 | |
| Melbourne Causeway, Middle Estuary | | 2.7178 | | 0.1008 | | 0.0094 | |
| Melbourne Causeway, North Fork | | 4.0636 | | 0.1005 | | 0.001 | |
| Melbourne Causeway, Sebastian Inlet | | 3.267 | | 0.1002 | | 0.0043 | |
| Melbourne Causeway, South Fork | | 4.2245 | | 0.0978 | | 0.0025 | |
| Melbourne Causeway, South Fork 2 | | 4.8845 | | 0.0996 | | 0.0011 | |
| Melbourne Causeway, Vero Beach | | 3.7939 | | 0.0968 | | 0.0035 | |
| Merritt Island Causeway, Manatee Pocket | | 3.3874 | | 0.1 | | 0.0044 | |
| Merritt Island Causeway, North Fork | | 3.9151 | | 0.0956 | | 0.0032 | |
| Merritt Island Causeway, Sebastian Inlet | | 2.8209 | | 0.099 | | 0.0086 | |
| Merritt Island Causeway, South Fork | | 4.1786 | | 0.098 | | 0.0017 | |
| Merritt Island Causeway, South Fork 2 | | 4.7022 | | 0.1005 | | 0.0013 | |
| Merritt Island Causeway, Vero Beach | | 3.2458 | | 0.097 | | 0.0046 | |
| Middle Estuary, Manatee Pocket | | 1.7818 | | 0.0982 | | 0.0554 | |
| **Overall Parameter Category** | **Pseudo-F** | | P(perms) | | P(MC) | |  |
| Pair-wise test category | t statistic | |  |  |  |  |  |
| **Mar/Apr 2017 by Site** | | | | | | |  |
| Middle Estuary, Merritt Island Causeway | | 2.7421 | | 0.0977 | | 0.0107 | |
| Middle Estuary, North Fork | | 1.5936 | | 0.099 | | 0.0896 | |
| Middle Estuary, Sebastian Inlet | | 2.3876 | | 0.1001 | | 0.0171 | |
| Middle Estuary, South Fork | | 1.7685 | | 0.1013 | | 0.0615 | |
| Middle Estuary, South Fork 2 | | 2.6238 | | 0.0992 | | 0.0106 | |
| Middle Estuary, Vero Beach | | 2.4585 | | 0.0995 | | 0.0136 | |
| North Fork, Sebastian Inlet | | 3.3968 | | 0.0988 | | 0.0034 | |
| North Fork, South Fork | | 2.4373 | | 0.0979 | | 0.0125 | |
| North Fork, South Fork 2 | | 3.4096 | | 0.0946 | | 0.0047 | |
| North Fork, Vero Beach | | 3.5335 | | 0.1029 | | 0.0037 | |
| Sebastian Inlet, South Fork 2 | | 4.0398 | | 0.1001 | | 0.0018 | |
| Sebastian Inlet, Vero Beach | | 2.258 | | 0.1031 | | 0.0179 | |
| South Fork 2, Vero Beach | | 4.5288 | | 0.0979 | | 0.0011 | |
| South Fork, Sebastian Inlet | | 3.6076 | | 0.0975 | | 0.0041 | |
| South Fork, South Fork 2 | | 2.677 | | 0.1038 | | 0.0106 | |
| South Fork, Vero Beach | | 3.9314 | | 0.1008 | | 0.0025 | |
| **Oct/Nov 2017 by Site** | | **4.7368** | | **0.0001** | | **0.0001** | |
| Barber Bridge, Fort Pierce | | 1.753 | | 0.1033 | | 0.0471 | |
| Barber Bridge, Harbor Branch Channel | | 1.9624 | | 0.1038 | | 0.0283 | |
| Barber Bridge, Harbortown Marina | | 2.3897 | | 0.0991 | | 0.0159 | |
| Barber Bridge, Hobe Sound NWR | | 1.7497 | | 0.0977 | | 0.0524 | |
| Barber Bridge, Jensen Beach | | 1.6542 | | 0.1033 | | 0.0634 | |
| Barber Bridge, Jupiter Narrows | | 1.7671 | | 0.0975 | | 0.0441 | |
| Barber Bridge, Linkport | | 1.4696 | | 0.1013 | | 0.1127 | |
| Barber Bridge, Manatee Pocket | | 2.0121 | | 0.0953 | | 0.027 | |
| Barber Bridge, Melbourne Causeway | | 2.0939 | | 0.0953 | | 0.0237 | |
| Barber Bridge, Merritt Island Causeway | | 2.1535 | | 0.1018 | | 0.0187 | |
| Barber Bridge, Middle Estuary | | 2.9191 | | 0.1007 | | 0.0065 | |
| Barber Bridge, North Fork | | 2.2042 | | 0.1 | | 0.0188 | |
| Barber Bridge, Round Island | | 1.4538 | | 0.0958 | | 0.1122 | |
| Barber Bridge, Sebastian Inlet | | 1.5786 | | 0.0948 | | 0.0774 | |
| Barber Bridge, South Fork | | 2.9864 | | 0.1015 | | 0.0057 | |
| Barber Bridge, South Fork 2 | | 2.1587 | | 0.1029 | | 0.0207 | |
| Barber Bridge, Vero Beach | | 1.3913 | | 0.0973 | | 0.1413 | |
| Barber Bridge, Vero Beach Marina | | 1.2341 | | 0.0984 | | 0.2266 | |
| Fort Pierce, Harbor Branch Channel | | 2.0629 | | 0.0987 | | 0.0252 | |
| Fort Pierce, Harbortown Marina | | 2.1668 | | 0.0984 | | 0.0234 | |
| Fort Pierce, Hobe Sound NWR | | 1.6807 | | 0.0958 | | 0.0625 | |
| Fort Pierce, Jensen Beach | | 1.6337 | | 0.0945 | | 0.0651 | |
| **Overall Parameter Category** | **Pseudo-F** | | P(perms) | | P(MC) | |  |
| Pair-wise test category | t statistic | |  |  |  |  |  |
| **Oct/Nov 2017 by Site** | | | | | | |  |
| Fort Pierce, Jupiter Narrows | | 1.7512 | | 0.0999 | | 0.0484 | |
| Fort Pierce, Linkport | | 1.6916 | | 0.1035 | | 0.0582 | |
| Fort Pierce, Manatee Pocket | | 2.0494 | | 0.101 | | 0.0239 | |
| Fort Pierce, Melbourne Causeway | | 2.3989 | | 0.1024 | | 0.0128 | |
| Fort Pierce, Merritt Island Causeway | | 2.176 | | 0.0969 | | 0.0199 | |
| Fort Pierce, Middle Estuary | | 2.849 | | 0.1036 | | 0.0085 | |
| Fort Pierce, North Fork | | 2.3043 | | 0.0974 | | 0.0167 | |
| Fort Pierce, Round Island | | 1.7649 | | 0.0984 | | 0.0504 | |
| Fort Pierce, Sebastian Inlet | | 1.4541 | | 0.0973 | | 0.1125 | |
| Fort Pierce, South Fork | | 2.9087 | | 0.1002 | | 0.0077 | |
| Fort Pierce, South Fork 2 | | 2.1728 | | 0.0988 | | 0.0235 | |
| Fort Pierce, Vero Beach | | 1.6835 | | 0.096 | | 0.0611 | |
| Fort Pierce, Vero Beach Marina | | 1.8444 | | 0.1002 | | 0.0382 | |
| Harbor Branch Channel, Harbortown Marina | | 2.2489 | | 0.1018 | | 0.0184 | |
| Harbor Branch Channel, Hobe Sound NWR | | 1.8838 | | 0.1056 | | 0.0376 | |
| Harbor Branch Channel, Jensen Beach | | 2.1017 | | 0.1023 | | 0.0244 | |
| Harbor Branch Channel, Jupiter Narrows | | 1.9907 | | 0.1033 | | 0.0281 | |
| Harbor Branch Channel, Linkport | | 1.8598 | | 0.0972 | | 0.0404 | |
| Harbor Branch Channel, Manatee Pocket | | 2.1286 | | 0.1018 | | 0.0223 | |
| Harbor Branch Channel, Melbourne Causeway | | 2.1041 | | 0.0955 | | 0.0232 | |
| Harbor Branch Channel, Merritt Island Causeway | | 2.1192 | | 0.0998 | | 0.0226 | |
| Harbor Branch Channel, Middle Estuary | | 2.7874 | | 0.1015 | | 0.008 | |
| Harbor Branch Channel, North Fork | | 2.2645 | | 0.103 | | 0.0153 | |
| Harbor Branch Channel, Round Island | | 1.8881 | | 0.1004 | | 0.034 | |
| Harbor Branch Channel, Sebastian Inlet | | 1.8195 | | 0.0976 | | 0.041 | |
| Harbor Branch Channel, South Fork | | 2.845 | | 0.1021 | | 0.0068 | |
| Harbor Branch Channel, South Fork 2 | | 2.0805 | | 0.1041 | | 0.0263 | |
| Harbor Branch Channel, Vero Beach | | 2.1264 | | 0.0961 | | 0.0244 | |
| Harbor Branch Channel, Vero Beach Marina | | 1.9347 | | 0.1011 | | 0.0302 | |
| Harbortown Marina, Jensen Beach | | 2.3282 | | 0.0984 | | 0.0147 | |
| Harbortown Marina, Jupiter Narrows | | 2.2982 | | 0.0954 | | 0.0159 | |
| Harbortown Marina, Linkport | | 2.3463 | | 0.1002 | | 0.0135 | |
| Harbortown Marina, Manatee Pocket | | 2.4352 | | 0.1016 | | 0.0158 | |
| Harbortown Marina, Melbourne Causeway | | 2.6563 | | 0.1018 | | 0.0087 | |
| Harbortown Marina, Merritt Island Causeway | | 2.6569 | | 0.0999 | | 0.0108 | |
| Harbortown Marina, Middle Estuary | | 2.5734 | | 0.0982 | | 0.0103 | |
| Harbortown Marina, North Fork | | 2.3296 | | 0.0975 | | 0.0167 | |
| Harbortown Marina, Round Island | | 2.5006 | | 0.1012 | | 0.0119 | |
| Harbortown Marina, Sebastian Inlet | | 2.1889 | | 0.1002 | | 0.018 | |
| Harbortown Marina, South Fork | | 2.5571 | | 0.0981 | | 0.0119 | |
| **Overall Parameter Category** | **Pseudo-F** | | P(perms) | | P(MC) | |  |
| Pair-wise test category | t statistic | |  |  |  |  |  |
| **Oct/Nov 2017 by Site** | | | | | | |  |
| Harbortown Marina, South Fork 2 | | 1.8507 | | 0.0963 | | 0.0471 | |
| Harbortown Marina, Vero Beach | | 2.6169 | | 0.0964 | | 0.0092 | |
| Harbortown Marina, Vero Beach Marina | | 2.448 | | 0.1017 | | 0.0125 | |
| Hobe Sound NWR, Harbortown Marina | | 2.3942 | | 0.0969 | | 0.0144 | |
| Hobe Sound NWR, Jensen Beach | | 1.753 | | 0.1047 | | 0.0481 | |
| Hobe Sound NWR, Jupiter Narrows | | 1.6616 | | 0.0971 | | 0.0615 | |
| Hobe Sound NWR, Linkport | | 1.6394 | | 0.1018 | | 0.0654 | |
| Hobe Sound NWR, Manatee Pocket | | 2.0544 | | 0.1073 | | 0.0267 | |
| Hobe Sound NWR, Melbourne Causeway | | 2.3088 | | 0.1084 | | 0.0168 | |
| Hobe Sound NWR, Merritt Island Causeway | | 2.1545 | | 0.1031 | | 0.0199 | |
| Hobe Sound NWR, Middle Estuary | | 2.8943 | | 0.1042 | | 0.008 | |
| Hobe Sound NWR, North Fork | | 2.321 | | 0.0975 | | 0.0156 | |
| Hobe Sound NWR, Round Island | | 1.6007 | | 0.1002 | | 0.0721 | |
| Hobe Sound NWR, Sebastian Inlet | | 1.5308 | | 0.1052 | | 0.0966 | |
| Hobe Sound NWR, South Fork | | 2.963 | | 0.0991 | | 0.0074 | |
| Hobe Sound NWR, South Fork 2 | | 2.1883 | | 0.0993 | | 0.0211 | |
| Hobe Sound NWR, Vero Beach | | 1.7881 | | 0.1013 | | 0.0454 | |
| Hobe Sound NWR, Vero Beach Marina | | 1.8158 | | 0.0997 | | 0.0398 | |
| Jensen Beach, Jupiter Narrows | | 1.5774 | | 0.0977 | | 0.076 | |
| Jensen Beach, Linkport | | 1.8039 | | 0.1041 | | 0.0401 | |
| Jensen Beach, Manatee Pocket | | 1.7281 | | 0.1031 | | 0.049 | |
| Jensen Beach, Melbourne Causeway | | 2.343 | | 0.0982 | | 0.0153 | |
| Jensen Beach, Merritt Island Causeway | | 2.3493 | | 0.0956 | | 0.0145 | |
| Jensen Beach, Middle Estuary | | 2.6672 | | 0.0975 | | 0.0082 | |
| Jensen Beach, North Fork | | 1.9411 | | 0.093 | | 0.0312 | |
| Jensen Beach, Round Island | | 1.8195 | | 0.0949 | | 0.041 | |
| Jensen Beach, Sebastian Inlet | | 1.635 | | 0.0987 | | 0.0675 | |
| Jensen Beach, South Fork | | 2.7621 | | 0.1024 | | 0.009 | |
| Jensen Beach, South Fork 2 | | 2.0868 | | 0.1005 | | 0.0275 | |
| Jensen Beach, Vero Beach | | 1.6842 | | 0.1001 | | 0.0548 | |
| Jensen Beach, Vero Beach Marina | | 1.6976 | | 0.097 | | 0.0572 | |
| Jupiter Narrows, Linkport | | 1.9578 | | 0.098 | | 0.0337 | |
| Jupiter Narrows, Manatee Pocket | | 1.6992 | | 0.0985 | | 0.0581 | |
| Jupiter Narrows, Melbourne Causeway | | 2.3429 | | 0.0979 | | 0.0147 | |
| Jupiter Narrows, Merritt Island Causeway | | 2.1638 | | 0.0935 | | 0.0224 | |
| Jupiter Narrows, Middle Estuary | | 2.5782 | | 0.1004 | | 0.0093 | |
| Jupiter Narrows, North Fork | | 1.9475 | | 0.0985 | | 0.0298 | |
| Jupiter Narrows, Round Island | | 1.9496 | | 0.0936 | | 0.0307 | |
| Jupiter Narrows, Sebastian Inlet | | 1.6728 | | 0.1011 | | 0.0618 | |
| Jupiter Narrows, South Fork | | 2.6492 | | 0.1003 | | 0.0106 | |
| **Overall Parameter Category** | **Pseudo-F** | | P(perms) | | P(MC) | |  |
| Pair-wise test category | t statistic | |  |  |  |  |  |
| **Oct/Nov 2017 by Site** | | | | | | |  |
| Jupiter Narrows, South Fork 2 | | 2.001 | | 0.0993 | | 0.0285 | |
| Jupiter Narrows, Vero Beach | | 1.784 | | 0.1015 | | 0.0492 | |
| Jupiter Narrows, Vero Beach Marina | | 1.7888 | | 0.1001 | | 0.0448 | |
| Linkport, Manatee Pocket | | 2.2265 | | 0.1067 | | 0.0162 | |
| Linkport, Melbourne Causeway | | 1.9715 | | 0.1029 | | 0.0309 | |
| Linkport, Merritt Island Causeway | | 2.1412 | | 0.1043 | | 0.0241 | |
| Linkport, Middle Estuary | | 3.0317 | | 0.0989 | | 0.0065 | |
| Linkport, North Fork | | 2.4038 | | 0.0941 | | 0.0145 | |
| Linkport, Round Island | | 1.4587 | | 0.1013 | | 0.1152 | |
| Linkport, Sebastian Inlet | | 1.6675 | | 0.1015 | | 0.0655 | |
| Linkport, South Fork | | 3.0964 | | 0.1038 | | 0.0065 | |
| Linkport, South Fork 2 | | 2.2451 | | 0.0971 | | 0.0195 | |
| Linkport, Vero Beach | | 1.6644 | | 0.1008 | | 0.0611 | |
| Linkport, Vero Beach Marina | | 1.5503 | | 0.0971 | | 0.0893 | |
| Manatee Pocket, North Fork | | 1.9457 | | 0.099 | | 0.033 | |
| Manatee Pocket, Round Island | | 2.2428 | | 0.1017 | | 0.0167 | |
| Manatee Pocket, Sebastian Inlet | | 1.8959 | | 0.1017 | | 0.0343 | |
| Manatee Pocket, South Fork | | 2.8152 | | 0.101 | | 0.0096 | |
| Manatee Pocket, South Fork 2 | | 2.111 | | 0.1002 | | 0.0235 | |
| Manatee Pocket, Vero Beach | | 2.2123 | | 0.0995 | | 0.0189 | |
| Manatee Pocket, Vero Beach Marina | | 1.9122 | | 0.0997 | | 0.0328 | |
| Melbourne Causeway, Manatee Pocket | | 2.4996 | | 0.1019 | | 0.0127 | |
| Melbourne Causeway, Merritt Island Causeway | | 2.2788 | | 0.0991 | | 0.0189 | |
| Melbourne Causeway, Middle Estuary | | 3.2007 | | 0.102 | | 0.0069 | |
| Melbourne Causeway, North Fork | | 2.5051 | | 0.0993 | | 0.013 | |
| Melbourne Causeway, Round Island | | 2.1657 | | 0.0974 | | 0.0192 | |
| Melbourne Causeway, Sebastian Inlet | | 2.1248 | | 0.0992 | | 0.0226 | |
| Melbourne Causeway, South Fork | | 3.2854 | | 0.099 | | 0.0047 | |
| Melbourne Causeway, South Fork 2 | | 2.3377 | | 0.1038 | | 0.0176 | |
| Melbourne Causeway, Vero Beach | | 2.3313 | | 0.1049 | | 0.0174 | |
| Melbourne Causeway, Vero Beach Marina | | 1.9849 | | 0.1013 | | 0.0266 | |
| Merritt Island Causeway, Manatee Pocket | | 2.4252 | | 0.1044 | | 0.0124 | |
| Merritt Island Causeway, North Fork | | 2.4445 | | 0.0995 | | 0.0138 | |
| Merritt Island Causeway, Round Island | | 2.0557 | | 0.0955 | | 0.0237 | |
| Merritt Island Causeway, Sebastian Inlet | | 1.9 | | 0.0999 | | 0.0315 | |
| Merritt Island Causeway, South Fork | | 3.0607 | | 0.1004 | | 0.0058 | |
| Merritt Island Causeway, South Fork 2 | | 2.2507 | | 0.0992 | | 0.0193 | |
| Merritt Island Causeway, Vero Beach | | 2.2369 | | 0.098 | | 0.0166 | |
| Merritt Island Causeway, Vero Beach Marina | | 2.1928 | | 0.0969 | | 0.0189 | |
| Middle Estuary, Manatee Pocket | | 2.7058 | | 0.0984 | | 0.0101 | |
| **Overall Parameter Category** | **Pseudo-F** | | P(perms) | | P(MC) | |  |
| Pair-wise test category | t statistic | |  |  |  |  |  |
| **Oct/Nov 2017 by Site** | | | | | | |  |
| Middle Estuary, Merritt Island Causeway | | 3.0004 | | 0.0973 | | 0.006 | |
| Middle Estuary, North Fork | | 1.9197 | | 0.0985 | | 0.038 | |
| Middle Estuary, Round Island | | 3.0303 | | 0.1008 | | 0.0067 | |
| Middle Estuary, Sebastian Inlet | | 2.6691 | | 0.0966 | | 0.0089 | |
| Middle Estuary, South Fork | | 1.2608 | | 0.0998 | | 0.2128 | |
| Middle Estuary, South Fork 2 | | 1.7084 | | 0.0975 | | 0.0634 | |
| Middle Estuary, Vero Beach | | 3.0442 | | 0.0988 | | 0.0063 | |
| Middle Estuary, Vero Beach Marina | | 2.9622 | | 0.099 | | 0.0071 | |
| North Fork, Round Island | | 2.3819 | | 0.1028 | | 0.0148 | |
| North Fork, Sebastian Inlet | | 2.1522 | | 0.1065 | | 0.019 | |
| North Fork, South Fork | | 2.1378 | | 0.0949 | | 0.0223 | |
| North Fork, South Fork 2 | | 1.6382 | | 0.0962 | | 0.0724 | |
| North Fork, Vero Beach | | 2.359 | | 0.1009 | | 0.014 | |
| North Fork, Vero Beach Marina | | 2.2313 | | 0.0998 | | 0.0196 | |
| Round Island, Sebastian Inlet | | 1.5295 | | 0.1019 | | 0.0947 | |
| Round Island, South Fork | | 3.097 | | 0.1013 | | 0.0064 | |
| Round Island, South Fork 2 | | 2.2658 | | 0.1006 | | 0.0174 | |
| Round Island, Vero Beach | | 1.4587 | | 0.0947 | | 0.1131 | |
| Round Island, Vero Beach Marina | | 1.5088 | | 0.1034 | | 0.0905 | |
| Sebastian Inlet, South Fork 2 | | 2.0716 | | 0.1036 | | 0.0251 | |
| Sebastian Inlet, Vero Beach | | 1.4804 | | 0.1025 | | 0.104 | |
| Sebastian Inlet, Vero Beach Marina | | 1.5857 | | 0.1008 | | 0.0789 | |
| South Fork 2, Vero Beach | | 2.2919 | | 0.0975 | | 0.0191 | |
| South Fork 2, Vero Beach Marina | | 2.2277 | | 0.0982 | | 0.0204 | |
| South Fork, Sebastian Inlet | | 2.7271 | | 0.0973 | | 0.0087 | |
| South Fork, South Fork 2 | | 1.6146 | | 0.0985 | | 0.0816 | |
| South Fork, Vero Beach | | 3.1122 | | 0.1044 | | 0.005 | |
| South Fork, Vero Beach Marina | | 3.0437 | | 0.0993 | | 0.006 | |
| Vero Beach, Vero Beach Marina | | 1.544 | | 0.0961 | |  | |
| **Apr 2018 by Site** | | **5.9778** | | **0.0001** | | **0.0001** | |
| Barber Bridge, Fort Pierce | | 2.5134 | | 0.1017 | | 0.0115 | |
| Barber Bridge, Harbor Branch Channel | | 2.1416 | | 0.1003 | | 0.0199 | |
| Barber Bridge, Harbortown Marina | | 2.26 | | 0.0979 | | 0.0182 | |
| Barber Bridge, Hobe Sound NWR | | 2.063 | | 0.1027 | | 0.0262 | |
| Barber Bridge, Jensen Beach | | 1.9171 | | 0.0989 | | 0.0345 | |
| Barber Bridge, Jupiter Narrows | | 1.9364 | | 0.096 | | 0.0295 | |
| Barber Bridge, Linkport | | 1.6553 | | 0.0992 | | 0.0691 | |
| Barber Bridge, Manatee Pocket | | 2.2269 | | 0.102 | | 0.0213 | |
| Barber Bridge, Melbourne Causeway | | 2.3054 | | 0.1001 | | 0.0178 | |
| Barber Bridge, Merritt Island Causeway | | 2.9873 | | 0.0996 | | 0.0079 | |
| **Overall Parameter Category** | **Pseudo-F** | | P(perms) | | P(MC) | |  |
| Pair-wise test category | t statistic | |  |  |  |  |  |
| **Apr 2018 by Site** | | | | | | |  |
| Barber Bridge, Middle Estuary | | 2.6417 | | 0.096 | | 0.0102 | |
| Barber Bridge, North Fork | | 2.761 | | 0.1033 | | 0.0083 | |
| Barber Bridge, Round Island | | 1.5333 | | 0.0962 | | 0.0899 | |
| Barber Bridge, Sebastian Inlet | | 1.7192 | | 0.098 | | 0.0508 | |
| Barber Bridge, South Fork | | 3.0196 | | 0.0978 | | 0.0065 | |
| Barber Bridge, South Fork 2 | | 3.5449 | | 0.0994 | | 0.0036 | |
| Barber Bridge, Vero Beach | | 1.4519 | | 0.0948 | | 0.1172 | |
| Barber Bridge, Vero Beach Marina | | 1.16 | | 0.2046 | | 0.2881 | |
| Fort Pierce, Harbor Branch Channel | | 2.6062 | | 0.0951 | | 0.0099 | |
| Fort Pierce, Harbortown Marina | | 2.3106 | | 0.1099 | | 0.0177 | |
| Fort Pierce, Hobe Sound NWR | | 2.1355 | | 0.0981 | | 0.0224 | |
| Fort Pierce, Jensen Beach | | 2.1285 | | 0.1014 | | 0.0194 | |
| Fort Pierce, Jupiter Narrows | | 2.0542 | | 0.0955 | | 0.0259 | |
| Fort Pierce, Linkport | | 2.1516 | | 0.0962 | | 0.0238 | |
| Fort Pierce, Manatee Pocket | | 2.5315 | | 0.1 | | 0.012 | |
| Fort Pierce, Melbourne Causeway | | 3.1709 | | 0.0967 | | 0.0064 | |
| Fort Pierce, Merritt Island Causeway | | 3.1795 | | 0.1 | | 0.0041 | |
| Fort Pierce, Middle Estuary | | 2.8267 | | 0.0967 | | 0.0091 | |
| Fort Pierce, North Fork | | 2.979 | | 0.1023 | | 0.0064 | |
| Fort Pierce, Round Island | | 2.1883 | | 0.1019 | | 0.0211 | |
| Fort Pierce, Sebastian Inlet | | 1.8762 | | 0.0999 | | 0.0355 | |
| Fort Pierce, South Fork | | 3.2002 | | 0.1044 | | 0.0049 | |
| Fort Pierce, South Fork 2 | | 3.6221 | | 0.1029 | | 0.0026 | |
| Fort Pierce, Vero Beach | | 2.053 | | 0.1021 | | 0.0248 | |
| Fort Pierce, Vero Beach Marina | | 2.6327 | | 0.1004 | | 0.0094 | |
| Harbor Branch Channel, Harbortown Marina | | 1.7503 | | 0.1049 | | 0.0533 | |
| Harbor Branch Channel, Hobe Sound NWR | | 2.0897 | | 0.0993 | | 0.0241 | |
| Harbor Branch Channel, Jensen Beach | | 2.4245 | | 0.101 | | 0.0143 | |
| Harbor Branch Channel, Jupiter Narrows | | 2.1201 | | 0.0967 | | 0.0233 | |
| Harbor Branch Channel, Linkport | | 1.6049 | | 0.1009 | | 0.0736 | |
| Harbor Branch Channel, Manatee Pocket | | 2.2027 | | 0.104 | | 0.0193 | |
| Harbor Branch Channel, Melbourne Causeway | | 2.2802 | | 0.1009 | | 0.0159 | |
| Harbor Branch Channel, Merritt Island Causeway | | 2.7138 | | 0.1012 | | 0.0086 | |
| Harbor Branch Channel, Middle Estuary | | 2.6548 | | 0.0971 | | 0.0104 | |
| Harbor Branch Channel, North Fork | | 2.7942 | | 0.1019 | | 0.0088 | |
| Harbor Branch Channel, Round Island | | 2.1304 | | 0.096 | | 0.0197 | |
| Harbor Branch Channel, Sebastian Inlet | | 1.976 | | 0.0991 | | 0.0269 | |
| Harbor Branch Channel, South Fork | | 2.8815 | | 0.102 | | 0.0081 | |
| Harbor Branch Channel, South Fork 2 | | 3.362 | | 0.0986 | | 0.0043 | |
| Harbor Branch Channel, Vero Beach | | 2.244 | | 0.0993 | | 0.0186 | |
| **Overall Parameter Category** | **Pseudo-F** | | P(perms) | | P(MC) | |  |
| Pair-wise test category | t statistic | |  |  |  |  |  |
| **Apr 2018 by Site** | | | | | | |  |
| Harbor Branch Channel, Vero Beach Marina | | 2.3398 | | 0.0949 | | 0.0163 | |
| Harbortown Marina, Jensen Beach | | 2.2622 | | 0.0984 | | 0.0188 | |
| Harbortown Marina, Jupiter Narrows | | 2.0712 | | 0.0986 | | 0.0289 | |
| Harbortown Marina, Linkport | | 1.7228 | | 0.0992 | | 0.0539 | |
| Harbortown Marina, Manatee Pocket | | 2.1228 | | 0.1009 | | 0.0232 | |
| Harbortown Marina, Melbourne Causeway | | 2.3124 | | 0.1043 | | 0.0162 | |
| Harbortown Marina, Merritt Island Causeway | | 2.5639 | | 0.1059 | | 0.0121 | |
| Harbortown Marina, Middle Estuary | | 2.2595 | | 0.0983 | | 0.0207 | |
| Harbortown Marina, North Fork | | 2.5008 | | 0.1034 | | 0.0128 | |
| Harbortown Marina, Round Island | | 2.2029 | | 0.0983 | | 0.0196 | |
| Harbortown Marina, Sebastian Inlet | | 1.9815 | | 0.0992 | | 0.0305 | |
| Harbortown Marina, South Fork | | 2.3825 | | 0.1018 | | 0.0156 | |
| Harbortown Marina, South Fork 2 | | 2.5985 | | 0.1007 | | 0.011 | |
| Harbortown Marina, Vero Beach | | 2.2285 | | 0.0972 | | 0.0201 | |
| Harbortown Marina, Vero Beach Marina | | 2.4276 | | 0.0978 | | 0.0124 | |
| Hobe Sound NWR, Harbortown Marina | | 2.0432 | | 0.1042 | | 0.0257 | |
| Hobe Sound NWR, Jensen Beach | | 1.885 | | 0.1005 | | 0.0381 | |
| Hobe Sound NWR, Jupiter Narrows | | 1.5628 | | 0.1035 | | 0.0813 | |
| Hobe Sound NWR, Linkport | | 1.7263 | | 0.0986 | | 0.0526 | |
| Hobe Sound NWR, Manatee Pocket | | 2.1464 | | 0.1012 | | 0.022 | |
| Hobe Sound NWR, Melbourne Causeway | | 2.747 | | 0.1043 | | 0.0091 | |
| Hobe Sound NWR, Merritt Island Causeway | | 2.965 | | 0.0985 | | 0.0066 | |
| Hobe Sound NWR, Middle Estuary | | 2.5509 | | 0.1028 | | 0.0112 | |
| Hobe Sound NWR, North Fork | | 2.8632 | | 0.1022 | | 0.0082 | |
| Hobe Sound NWR, Round Island | | 1.8984 | | 0.1019 | | 0.0367 | |
| Hobe Sound NWR, Sebastian Inlet | | 1.7052 | | 0.1052 | | 0.056 | |
| Hobe Sound NWR, South Fork | | 2.9996 | | 0.1003 | | 0.0063 | |
| Hobe Sound NWR, South Fork 2 | | 3.5274 | | 0.0985 | | 0.0043 | |
| Hobe Sound NWR, Vero Beach | | 1.9074 | | 0.1017 | | 0.0355 | |
| Hobe Sound NWR, Vero Beach Marina | | 2.2631 | | 0.1021 | | 0.0175 | |
| Jensen Beach, Jupiter Narrows | | 1.6759 | | 0.1017 | | 0.0612 | |
| Jensen Beach, Linkport | | 1.9688 | | 0.1042 | | 0.0312 | |
| Jensen Beach, Manatee Pocket | | 2.0869 | | 0.0996 | | 0.0209 | |
| Jensen Beach, Melbourne Causeway | | 2.8862 | | 0.1016 | | 0.0062 | |
| Jensen Beach, Merritt Island Causeway | | 3.2055 | | 0.0994 | | 0.0058 | |
| Jensen Beach, Middle Estuary | | 2.4688 | | 0.1029 | | 0.0127 | |
| Jensen Beach, North Fork | | 2.6941 | | 0.1002 | | 0.0091 | |
| Jensen Beach, Round Island | | 1.8694 | | 0.0996 | | 0.0364 | |
| Jensen Beach, Sebastian Inlet | | 1.6363 | | 0.1017 | | 0.0741 | |
| Jensen Beach, South Fork | | 2.9789 | | 0.0966 | | 0.0047 | |
| **Overall Parameter Category** | **Pseudo-F** | | P(perms) | | P(MC) | |  |
| Pair-wise test category | t statistic | |  |  |  |  |  |
| **Apr 2018 by Site** | | | | | | |  |
| Jensen Beach, South Fork 2 | | 3.5756 | | 0.1011 | | 0.004 | |
| Jensen Beach, Vero Beach | | 1.6916 | | 0.1011 | | 0.0572 | |
| Jensen Beach, Vero Beach Marina | | 1.9658 | | 0.096 | | 0.0295 | |
| Jupiter Narrows, Linkport | | 1.8135 | | 0.1057 | | 0.0466 | |
| Jupiter Narrows, Manatee Pocket | | 1.8614 | | 0.099 | | 0.0382 | |
| Jupiter Narrows, Melbourne Causeway | | 2.5851 | | 0.0989 | | 0.0095 | |
| Jupiter Narrows, Merritt Island Causeway | | 2.8379 | | 0.1013 | | 0.0087 | |
| Jupiter Narrows, Middle Estuary | | 2.2977 | | 0.0939 | | 0.0171 | |
| Jupiter Narrows, North Fork | | 2.4739 | | 0.1052 | | 0.0117 | |
| Jupiter Narrows, Round Island | | 1.8652 | | 0.1011 | | 0.0394 | |
| Jupiter Narrows, Sebastian Inlet | | 1.7449 | | 0.0989 | | 0.0488 | |
| Jupiter Narrows, South Fork | | 2.694 | | 0.0986 | | 0.0105 | |
| Jupiter Narrows, South Fork 2 | | 3.2158 | | 0.1026 | | 0.0059 | |
| Jupiter Narrows, Vero Beach | | 1.8601 | | 0.106 | | 0.0377 | |
| Jupiter Narrows, Vero Beach Marina | | 2.0816 | | 0.0983 | | 0.0256 | |
| Linkport, Manatee Pocket | | 2.1272 | | 0.1035 | | 0.0221 | |
| Linkport, Melbourne Causeway | | 2.0036 | | 0.0931 | | 0.0272 | |
| Linkport, Merritt Island Causeway | | 2.4024 | | 0.1027 | | 0.014 | |
| Linkport, Middle Estuary | | 2.5089 | | 0.1033 | | 0.0126 | |
| Linkport, North Fork | | 2.6303 | | 0.1032 | | 0.0103 | |
| Linkport, Round Island | | 1.5786 | | 0.0994 | | 0.0838 | |
| Linkport, Sebastian Inlet | | 1.5805 | | 0.1054 | | 0.0831 | |
| Linkport, South Fork | | 2.7755 | | 0.0993 | | 0.008 | |
| Linkport, South Fork 2 | | 3.1417 | | 0.1016 | | 0.0059 | |
| Linkport, Vero Beach | | 1.7167 | | 0.1005 | | 0.06 | |
| Linkport, Vero Beach Marina | | 1.8644 | | 0.0984 | | 0.041 | |
| Manatee Pocket, North Fork | | 2.3685 | | 0.1022 | | 0.0142 | |
| Manatee Pocket, Round Island | | 2.2702 | | 0.1038 | | 0.0183 | |
| Manatee Pocket, Sebastian Inlet | | 1.9652 | | 0.1029 | | 0.028 | |
| Manatee Pocket, South Fork | | 2.5011 | | 0.1005 | | 0.0138 | |
| Manatee Pocket, South Fork 2 | | 3.1636 | | 0.1025 | | 0.006 | |
| Manatee Pocket, Vero Beach | | 2.294 | | 0.1001 | | 0.0169 | |
| Manatee Pocket, Vero Beach Marina | | 2.3076 | | 0.0947 | | 0.0174 | |
| Melbourne Causeway, Manatee Pocket | | 2.6308 | | 0.1008 | | 0.0098 | |
| Melbourne Causeway, Merritt Island Causeway | | 2.6496 | | 0.102 | | 0.0087 | |
| Melbourne Causeway, Middle Estuary | | 2.9875 | | 0.1006 | | 0.0052 | |
| Melbourne Causeway, North Fork | | 3.0743 | | 0.103 | | 0.0066 | |
| Melbourne Causeway, Round Island | | 2.4904 | | 0.098 | | 0.0125 | |
| Melbourne Causeway, Sebastian Inlet | | 2.2721 | | 0.0977 | | 0.019 | |
| Melbourne Causeway, South Fork | | 3.2392 | | 0.0988 | | 0.0037 | |
| **Overall Parameter Category** | **Pseudo-F** | | P(perms) | | P(MC) | |  |
| Pair-wise test category | t statistic | |  |  |  |  |  |
| **Apr 2018 by Site** | | | | | | |  |
| Melbourne Causeway, South Fork 2 | | 3.7559 | | 0.0981 | | 0.0023 | |
| Melbourne Causeway, Vero Beach | | 2.5816 | | 0.0977 | | 0.0133 | |
| Melbourne Causeway, Vero Beach Marina | | 2.5832 | | 0.0984 | | 0.0114 | |
| Merritt Island Causeway, Manatee Pocket | | 2.923 | | 0.0993 | | 0.0067 | |
| Merritt Island Causeway, North Fork | | 3.2979 | | 0.1015 | | 0.0043 | |
| Merritt Island Causeway, Round Island | | 2.7863 | | 0.1024 | | 0.0076 | |
| Merritt Island Causeway, Sebastian Inlet | | 2.4932 | | 0.1009 | | 0.012 | |
| Merritt Island Causeway, South Fork | | 3.5058 | | 0.102 | | 0.0035 | |
| Merritt Island Causeway, South Fork 2 | | 3.8918 | | 0.0989 | | 0.002 | |
| Merritt Island Causeway, Vero Beach | | 2.958 | | 0.1022 | | 0.0073 | |
| Merritt Island Causeway, Vero Beach Marina | | 3.2059 | | 0.1016 | | 0.0052 | |
| Middle Estuary, Manatee Pocket | | 2.0903 | | 0.0942 | | 0.0203 | |
| Middle Estuary, Merritt Island Causeway | | 3.3173 | | 0.1001 | | 0.0044 | |
| Middle Estuary, North Fork | | 1.7947 | | 0.1041 | | 0.0415 | |
| Middle Estuary, Round Island | | 2.6973 | | 0.1001 | | 0.0087 | |
| Middle Estuary, Sebastian Inlet | | 2.3888 | | 0.0999 | | 0.0138 | |
| Middle Estuary, South Fork | | 1.4487 | | 0.1012 | | 0.1137 | |
| Middle Estuary, South Fork 2 | | 2.5892 | | 0.1013 | | 0.0092 | |
| Middle Estuary, Vero Beach | | 2.6411 | | 0.0985 | | 0.0106 | |
| Middle Estuary, Vero Beach Marina | | 2.7856 | | 0.0979 | | 0.0091 | |
| North Fork, Round Island | | 2.7964 | | 0.1001 | | 0.0078 | |
| North Fork, Sebastian Inlet | | 2.5539 | | 0.1025 | | 0.0101 | |
| North Fork, South Fork | | 1.9372 | | 0.1007 | | 0.0298 | |
| North Fork, South Fork 2 | | 2.902 | | 0.1003 | | 0.0087 | |
| North Fork, Vero Beach | | 2.7767 | | 0.1005 | | 0.0077 | |
| North Fork, Vero Beach Marina | | 2.8581 | | 0.0996 | | 0.007 | |
| Round Island, Sebastian Inlet | | 1.5071 | | 0.1043 | | 0.099 | |
| Round Island, South Fork | | 3.0163 | | 0.1026 | | 0.0067 | |
| Round Island, South Fork 2 | | 3.4838 | | 0.0982 | | 0.0043 | |
| Round Island, Vero Beach | | 1.2416 | | 0.102 | | 0.2247 | |
| Round Island, Vero Beach Marina | | 1.6726 | | 0.1008 | | 0.0603 | |
| Sebastian Inlet, South Fork 2 | | 3.1253 | | 0.0991 | | 0.0043 | |
| Sebastian Inlet, Vero Beach | | 1.5268 | | 0.0974 | | 0.0911 | |
| Sebastian Inlet, Vero Beach Marina | | 1.8056 | | 0.1028 | | 0.0451 | |
| South Fork 2, Vero Beach | | 3.4746 | | 0.1041 | | 0.0039 | |
| South Fork 2, Vero Beach Marina | | 3.7667 | | 0.0951 | | 0.0025 | |
| South Fork, Sebastian Inlet | | 2.7254 | | 0.0958 | | 0.0086 | |
| South Fork, South Fork 2 | | 2.1209 | | 0.0967 | | 0.022 | |
| South Fork, Vero Beach | | 3.0141 | | 0.0977 | | 0.007 | |
| South Fork, Vero Beach Marina | | 3.1873 | | 0.0965 | | 0.006 | |
| **Overall Parameter Category** | **Pseudo-F** | | P(perms) | | P(MC) | |  |
| Pair-wise test category | t statistic | |  |  |  |  |  |
| **Apr 2018 by Site** | | | | | | |  |
| Vero Beach, Vero Beach Marina | | 1.528 | | 0.1003 | | 0.1046 | |
| **Sites by Sampling Periods** | | | | | | | |
| **Barber Bridge Oct/Nov 2017, Apr 2018** | | **2.2** | | **0.097** | | **0.11** | |
| **Fort Pierce by Sampling Period** | | **3.0** | | **0.0002** | | **0.0005** | |
| Mar/Apr 2017, Apr 2018 | | 1.4 | | 0.10 | | 0.15 | |
| Mar/Apr 2017, Aug/Sept 2016 | | 2.0 | | 0.10 | | 0.032 | |
| Mar/Apr 2017, Oct/Nov 2017 | | 1.5 | | 0.098 | | 0.10 | |
| Apr 2018, Aug/Sept 2016 | | 2.0 | | 0.10 | | 0.032 | |
| Apr 2018, Oct/Nov 2017 | | 1.5 | | 0.099 | | 0.093 | |
| Aug/Sept 2016, Oct/Nov 2017 | | 2.0 | | 0.10 | | 0.029 | |
| **Harbor Branch Channel by Sampling Period** | | **1.8** | | **0.0001** | | **0.0025** | |
| Mar/Apr 2017, Apr 2018 | | 1.4 | | 0.094 | | 0.12 | |
| Mar/Apr 2017, Aug/Sept 2016 | | 1.6 | | 0.10 | | 0.062 | |
| Mar/Apr 2017, Oct/Nov 2017 | | 1.7 | | 0.10 | | 0.064 | |
| Apr 2018, Aug/Sept 2016 | | 1.9 | | 0.10 | | 0.039 | |
| Apr 2018, Oct/Nov 2017 | | 1.5 | | 0.097 | | 0.092 | |
| Aug/Sept 2016, Oct/Nov 2017 | | 1.8 | | 0.098 | | 0.053 | |
| **Hobe Sound by Sampling Period** | | **3.4** | | **0.0001** | | **0.0009** | |
| Mar/Apr 2017, Apr 2018 | | 1.5 | | 0.099 | | 0.11 | |
| Mar/Apr 2017, Aug/Sept 2016 | | 2.1 | | 0.10 | | 0.022 | |
| Mar/Apr 2017, Oct/Nov 2017 | | 1.5 | | 0.10 | | 0.095 | |
| Apr 2018, Aug/Sept 2016 | | 2.2 | | 0.100 | | 0.020 | |
| Apr 2018, Oct/Nov 2017 | | 1.5 | | 0.10 | | 0.10 | |
| Aug/Sept 2016, Oct/Nov 2017 | | 2.2 | | 0.098 | | 0.021 | |
| **Harbortown Marina Oct/Nov 2017, Apr 2018** | | **2.4** | | **0.10** | | **0.088** | |
| **Jensen Beach by Sampling Period** | | **3.0** | | **0.0002** | | **0.0014** | |
| Mar/Apr 2017, Apr 2018 | | 1.5 | | 0.094 | | 0.097 | |
| Mar/Apr 2017, Aug/Sept 2016 | | 1.8 | | 0.10 | | 0.043 | |
| Mar/Apr 2017, Oct/Nov 2017 | | 1.6 | | 0.098 | | 0.077 | |
| Apr 2018, Aug/Sept 2016 | | 1.9 | | 0.10 | | 0.033 | |
| Apr 2018, Oct/Nov 2017 | | 1.5 | | 0.096 | | 0.11 | |
| Aug/Sept 2016, Oct/Nov 2017 | | 2.0 | | 0.10 | | 0.029 | |
| **Jupiter Narrows by Sampling Period** | | **2.4** | | **0.0001** | | **0.0046** | |
| Mar/Apr 2017, Apr 2018 | | 1.5 | | 0.10 | | 0.092 | |
| Mar/Apr 2017, Aug/Sept 2016 | | 1.5 | | 0.099 | | 0.090 | |
| Mar/Apr 2017, Oct/Nov 2017 | | 1.6 | | 0.097 | | 0.067 | |
| Apr 2018, Aug/Sept 2016 | | 1.7 | | 0.095 | | 0.058 | |
| Apr 2018, Oct/Nov 2017 | | 1.2 | | 0.098 | | 0.22 | |
| Aug/Sept 2016, Oct/Nov 2017 | | 1.6 | | 0.096 | | 0.076 | |
|  | |  | |  | |  | |
| **Overall Parameter Category** | **Pseudo-F** | | P(perms) | | P(MC) | |  |
| Pair-wise test category | t statistic | |  |  |  |  |  |
| **Linkport by Sampling Period** | | **2.4** | | **0.0001** | | **0.0052** | |
| Mar/Apr 2017, Apr 2018 | | 1.3 | | 0.10 | | 0.19 | |
| Mar/Apr 2017, Aug/Sept 2016 | | 1.4 | | 0.10 | | 0.15 | |
| Mar/Apr 2017, Oct/Nov 2017 | | 1.4 | | 0.099 | | 0.16 | |
| Apr 2018, Aug/Sept 2016 | | 1.9 | | 0.098 | | 0.038 | |
| Apr 2018, Oct/Nov 2017 | | 1.3 | | 0.10 | | 0.17 | |
| Aug/Sept 2016, Oct/Nov 2017 | | 1.9 | | 0.098 | | 0.035 | |
| **Melbourne Causeway by Sampling Period** | | **5.4** | | **0.0001** | | **0.0002** | |
| Mar/Apr 2017, Apr 2018 | | 2.1 | | 0.10 | | 0.022 | |
| Mar/Apr 2017, Aug/Sept 2016 | | 2.7 | | 0.10 | | 0.0094 | |
| Mar/Apr 2017, Oct/Nov 2017 | | 2.2 | | 0.10 | | 0.022 | |
| Apr 2018, Aug/Sept 2016 | | 2.4 | | 0.10 | | 0.015 | |
| Apr 2018, Oct/Nov 2017 | | 1.9 | | 0.10 | | 0.036 | |
| Aug/Sept 2016, Oct/Nov 2017 | | 2.7 | | 0.10 | | 0.010 | |
| **Middle Estuary by Sampling Period** | | **4.7** | | **0.0002** | | **0.0002** | |
| Mar/Apr 2017, Apr 2018 | | 1.3 | | 0.099 | | 0.19 | |
| Mar/Apr 2017, Aug/Sept 2016 | | 1.6 | | 0.090 | | 0.090 | |
| Mar/Apr 2017, Oct/Nov 2017 | | 2.4 | | 0.10 | | 0.016 | |
| Apr 2018, Aug/Sept 2016 | | 2.1 | | 0.10 | | 0.026 | |
| Apr 2018, Oct/Nov 2017 | | 2.3 | | 0.10 | | 0.015 | |
| Aug/Sept 2016, Oct/Nov 2017 | | 3.1 | | 0.094 | | 0.0058 | |
| **Merritt Island Causeway by Sampling Period** | | **5.5** | | **0.0001** | | **0.0001** | |
| Mar/Apr 2017, Apr 2018 | | 2.7 | | 0.096 | | 0.011 | |
| Mar/Apr 2017, Aug/Sept 2016 | | 3.1 | | 0.10 | | 0.0051 | |
| Mar/Apr 2017, Oct/Nov 2017 | | 2.1 | | 0.10 | | 0.029 | |
| Apr 2018, Aug/Sept 2016 | | 2.5 | | 0.097 | | 0.012 | |
| Apr 2018, Oct/Nov 2017 | | 1.8 | | 0.10 | | 0.049 | |
| Aug/Sept 2016, Oct/Nov 2017 | | 2.3 | | 0.096 | | 0.018 | |
| **Manatee Pocket by Sampling Period** | | **2.0** | | **0.0001** | | **0.0141** | |
| Mar/Apr 2017, Apr 2018 | | 1.3 | | 0.10 | | 0.20 | |
| Mar/Apr 2017, Aug/Sept 2016 | | 1.4 | | 0.10 | | 0.13 | |
| Mar/Apr 2017, Oct/Nov 2017 | | 1.3 | | 0.10 | | 0.17 | |
| Apr 2018, Aug/Sept 2016 | | 1.5 | | 0.10 | | 0.094 | |
| Apr 2018, Oct/Nov 2017 | | 1.2 | | 0.10 | | 0.25 | |
| Aug/Sept 2016, Oct/Nov 2017 | | 1.7 | | 0.10 | | 0.066 | |
| **North Fork by Sampling Period** | | **3.4** | | **0.0001** | | **0.0005** | |
| Mar/Apr 2017, Apr 2018 | | 1.4 | | 0.096 | | 0.14 | |
| Mar/Apr 2017, Aug/Sept 2016 | | 2.3 | | 0.10 | | 0.018 | |
| Mar/Apr 2017, Oct/Nov 2017 | | 1.8 | | 0.10 | | 0.051 | |
| Apr 2018, Aug/Sept 2016 | | 2.3 | | 0.098 | | 0.022 | |
| Apr 2018, Oct/Nov 2017 | | 1.4 | | 0.10 | | 0.13 | |
| **Overall Parameter Category** | **Pseudo-F** | | P(perms) | | P(MC) | |  |
| Pair-wise test category | t statistic | |  |  |  |  |  |
| **North Fork by Sampling Periods** | | | | | | |  |
| Aug/Sept 2016, Oct/Nov 2017 | | 2.1 | | 0.10 | | 0.024 | |
| **Round Island Oct/Nov 2017, Apr 2018** | | **3.1** | | **0.10** | | **0.046** | |
| **South Fork by Sampling Period** | | **5.0** | | **0.0002** | | **0.0001** | |
| Mar/Apr 2017, Apr 2018 | | 1.3 | | 0.098 | | 0.21 | |
| Mar/Apr 2017, Aug/Sept 2016 | | 2.0 | | 0.10 | | 0.027 | |
| Mar/Apr 2017, Oct/Nov 2017 | | 2.7 | | 0.10 | | 0.0083 | |
| Apr 2018, Aug/Sept 2016 | | 2.0 | | 0.099 | | 0.032 | |
| Apr 2018, Oct/Nov 2017 | | 2.4 | | 0.10 | | 0.015 | |
| Aug/Sept 2016, Oct/Nov 2017 | | 2.6 | | 0.10 | | 0.011 | |
| **Sebastian Inlet by Sampling Period** | | **2.5** | | **0.0002** | | **0.0066** | |
| Mar/Apr 2017, Apr 2018 | | 1.3 | | 0.099 | | 0.21 | |
| Mar/Apr 2017, Aug/Sept 2016 | | 1.4 | | 0.096 | | 0.15 | |
| Mar/Apr 2017, Oct/Nov 2017 | | 1.6 | | 0.10 | | 0.074 | |
| Apr 2018, Aug/Sept 2016 | | 1.7 | | 0.097 | | 0.055 | |
| Apr 2018, Oct/Nov 2017 | | 1.3 | | 0.10 | | 0.19 | |
| Aug/Sept 2016, Oct/Nov 2017 | | 2.0 | | 0.095 | | 0.032 | |
| **South Fork 2 by Sampling Period** | | **3.8** | | **0.0005** | | **0.0005** | |
| Mar/Apr 2017, Apr 2018 | | 1.4 | | 0.10 | | 0.15 | |
| Mar/Apr 2017, Aug/Sept 2016 | | 2.7 | | 0.10 | | 0.0092 | |
| Mar/Apr 2017, Oct/Nov 2017 | | 1.9 | | 0.10 | | 0.044 | |
| Apr 2018, Aug/Sept 2016 | | 2.4 | | 0.097 | | 0.015 | |
| Apr 2018, Oct/Nov 2017 | | 1.7 | | 0.096 | | 0.067 | |
| Aug/Sept 2016, Oct/Nov 2017 | | 1.9 | | 0.099 | | 0.041 | |
| **Vero Beach by Sampling Period** | | **4.0** | | **0.0001** | | **0.0003** | |
| Mar/Apr 2017, Apr 2018 | | 1.5 | | 0.10 | | 0.100 | |
| Mar/Apr 2017, Aug/Sept 2016 | | 2.2 | | 0.10 | | 0.022 | |
| Mar/Apr 2017, Oct/Nov 2017 | | 2.1 | | 0.10 | | 0.023 | |
| Apr 2018, Aug/Sept 2016 | | 1.9 | | 0.10 | | 0.039 | |
| Apr 2018, Oct/Nov 2017 | | 1.6 | | 0.10 | | 0.066 | |
| Aug/Sept 2016, Oct/Nov 2017 | | 2.6 | | 0.098 | | 0.013 | |
| **Vero Beach Marina Oct/Nov 2017, Apr 2018** | | **3.5** | | **0.099** | | **0.042** | |

^a^Bold text is associated with testing the overall differences within a category and ^b^regular text is associated with pair-wise testing. ^c^P(perms) stands for permutational p value, P(MC) for Monte-Carlo p value, ^e^TOM for total organic matter, ^f^Cu for copper, ^g^IRL stands for Indian River Lagoon and ^h^SLE for St. Lucie Estuary.
