# Supplementary material for: Defining the sediment prokaryotic communities of the Indian River Lagoon, FL, USA, an Estuary of National Significance
Source: PLoS One. 2020 Oct 26;15(10):e0236305. doi: 10.1371/journal.pone.0236305 (PMC7588086; doi:10.1371/journal.pone.0236305)
Supplement: S8 Table — aMarginal statistical tests are displayed for all variables and bsequential statistical results are shown only if the variable was determined to contribute a statistically significant amount of variation between microbial samples (p value < 0.05). cSS for sum of squares and dAICc stands for An Information Criterion. (DOCX) [file pone.0236305.s013.docx]

S8 Table: Distance-based linear model results

| Total Survey Distance Based Linear Model Results | | | | | |
| --- | --- | --- | --- | --- | --- |
| Marginal Tests^a^ | | | | | |
| Variable | SS(trace)^c^ | Pseudo-F | p value | Proportion. |  |
| Sediment temperature | 15264 | 4.7 | 0.0001 | 0.023 |  |
| Total organic matter | 55843 | 18 | 0.0001 | 0.083 |  |
| Porewater salinity | 61596 | 20 | 0.0001 | 0.092 |  |
| Percent gravel | 8416 | 2.6 | 0.003 | 0.013 |  |
|  |  |  |  |  |  |
| Copper concentration | 26399 | 8.3 | 0.0001 | 0.039 |  |
| Sequential tests^b^ | | | | | |
| Variable | AICcd | SS(trace) | Pseudo-F | p value | Proportion |
| Porewater salinity | 1637 | 61596 | 20 | 0.0001 | 0.092 |
| Total organic matter | 1624 | 43004 | 15 | 0.0001 | 0.064 |
| Copper concentration | 1618 | 20500 | 7.5 | 0.0001 | 0.031 |
| Sediment temperature | 1615 | 14827 | 5.5 | 0.0001 | 0.022 |

^a^Marginal statistical tests are displayed for all variables and ^b^sequential statistical results are shown only if the variable was determined to contribute a statistically significant amount of variation between microbial samples (p value < 0.05). ^c^SS for sum of squares and ^d^AICc stands for An Information Criterion.
