# Supplementary material for: Defining the sediment prokaryotic communities of the Indian River Lagoon, FL, USA, an Estuary of National Significance
Source: PLoS One. 2020 Oct 26;15(10):e0236305. doi: 10.1371/journal.pone.0236305 (PMC7588086; doi:10.1371/journal.pone.0236305)
Supplement: Attachment — Submitted filename: Response to Reviewers.docx [file pone.0236305.s015.docx]

We would like to thank Dr. Hans-Uwe Dahms and the reviewers for their time and comments. Please find below our response to each of your comments in bold. Thank you again for the opportunity to revise our manuscript with your helpful edits.

Reviewer comments made on pdf version of manuscript

Title/Authors/Affiliations Page

1. Comment while highlighting “microbiome” in Line 1: “Is it better to use prokaryotes or archaea/bacteria throughout the ms? You did not look into fungal, viral, protozoan diversities.”

**The reviewer correctly indicated that this is not a full microbiome study, thus all instances of “microbiome” or “microbial communities” that are in reference to just this study were changed to prokaryotes or bacterial/archaeal communities starting with the title (Starting with Line 4 of Revised Manuscript With Track Changes).**

Abstract

2. Edit of removing “a” from Line 19; corrected Line 22
3. Comment while highlighting “higher” in Lines 27-28: “higher in terms of abundance? or species richness?”

**Higher Betaproteobacteriales and Flavobacteriales were clarified to indicate that this referred to their relative abundances (Lines 31-32).**

4. Comment while highlighting “freshwater” in Line 27: “exclusively?”

**Thank you for indicating this needed clarification. Betaproteobacteriales is not exclusively a freshwater-associated bacterial group but has been shown in other studies to have a higher relative abundance in freshwater-associated regions of estuaries [1,2]. The words “a freshwater associated organism” were removed from the Abstract (Lines 31-32). This statement is more appropriate in just the Discussion section of the document where it can be further explained (Lines 483-484).**

5. Reviewer crossed out “organism” in Line 28 replacing with “bacterial group”, “organisms” in Line 30 replacing with “prokaryotes” and highlighted “organism” in Line 32.

**Thank you for providing alternative verbiage for the use of organism(s). References to organism(s) were changed to bacterial group(s) or prokaryote(s) throughout the document to reflect a more appropriate word choice starting with the Abstract (Starting with Line 26).**

Introduction

6. Comment while highlighting sentence “This study…TOM samples.” in Lines 78-82: “Can be more specific as you only looked into bacterial/archaeal diversities.”

**Please see response to Reviewer Comment #1 above.**

7. Comment while highlighting “Aug-Sep 2016 (W16), Mar-Apr 2017 (D17), Oct-Nov 2017(W17), and Apr 2018 (D18).” in Lines 115-116: “Can these be more specific? Were equal numbers of samples taken during each of the periods? Are the samples representative of the sites/seasons?”

**Thank you for indicating to us that our sampling strategy was unclear. The “Site selection” (Lines 96-108) and “Sample collection” (Lines 129-135) sections have been updated to make it more clear that 15 sites were sampled during the first two sampling periods and 19 sites during the second two sampling periods in triplicate to get representative samples of those sites and sampling periods.**

8. Reviewer highlighted MΩ in Line 131.

**This statement was updated to indicate that ultrapure water was used instead of defining the water used by its resistance (**MΩ**) (Line 150 and 159). The authors were unsure why this was highlighted and hope that this addresses the reviewer’s concern.**

Results

9. Comment while highlighting paragraph starting with Measured air temperatures…South Fork 2 site (S4 Table)” in Lines 199-204: “How can the data be treated as baseline data for future studies when the air temperatures and rainfall were abnormal during the sample years?

**As the reviewer correctly pointed out, this study will act as comparison data for future studies instead of as a baseline due to the abnormal environmental conditions during the sampling periods. The manuscript was updated to reflect this in the Abstract (Lines 39-40) and Conclusions (Lines 556).**

10. Edit add “.” to end of sentence in Line 278; corrected Line 308
11. Comment while highlighting subtitle “Microbial community makeup of estuaries” in Line 302: “Individual samples should be compared to examine variations of the prokaryotic diversities at the sample level. How variable were the diversities between samples collected at the different sampling sites during the different sampling months?”

**The authors appreciate this comment and agree that that the IRL versus SLE comparison will mask changes at the sample level. But, the comparison between the IRL and SLE samples is still important to explore due to the differences in freshwater discharge rates between the regions during the study and the distinct separation of samples in these regions in the PCoA (Fig 6). To further stress this point we added a paragraph outlining the differences in freshwater discharges between regions and sampling periods (Lines 227-234) and added another table (S4C Table) to the S4 Table group stressing the differences between the IRL and SLE.**

**To complement this regional comparison, we also grouped similar sample types together despite their regional and sampling period differences to focus on ecologically important anthropogenic impacts of muck and copper accumulation. This also allowed us to increase statistical power. This was clarified in Lines 101-104.**

**The reviewer is correct in that comparing samples at the site level over time or between sites during one sampling season is important. But due to our study design and alternative goals this is a low statistical power comparison since there were only three samples at each site during each sampling period. But to address this concern we conducted PERMANOVA analysis at each site between sampling seasons and during each sampling period between sites and added the results to S7 Table. The major trends are briefly discussed in Lines 387-402.**

12. Edit “make” to “made” in Line 332; corrected Line 362

Reviewer comments summarized in email

13. Reviewer #1: The reporting is straight-forward and clear. However, I do not agree with the approach of grouping of results from the individual samples based on the study locations (IRL and SLE). This would have masked the variations between the samples.

**Please see response to Comment #11 above.**

14. Reviewer #1: Also, the sampling strategy needs to be elaborated regarding the number of samples collected at each of the sites and their frequencies during the sampling periods.
15. Reviewer #2: The sampling strategies need more detail regarding the number, sites, and frequencies of the collected samples throughout the sampling periods.

**Please see response to Comment #7 above.**

16. Reviewer #1: The quality of the figures is too low.
17. Reviewer #2: Contrast of figures need to be enhanced.
18. While revising your submission, please upload your figure files to the Preflight Analysis and Conversion Engine (PACE) digital diagnostic tool, <https://pacev2.apexcovantage.com/>. PACE helps ensure that figures meet PLOS requirements. To use PACE, you must first register as a user. Registration is free. Then, login and navigate to the UPLOAD tab, where you will find detailed instructions on how to use the tool. If you encounter any issues or have any questions when using PACE, please email PLOS at [figures@plos.org](mailto:figures@plos.org). Please note that Supporting Information files do not need this step.

**PACE was utilized to determine that the figures did actually meet PLOS requirements since PACE did not make any corrections. PACE was used to rename the files according to PLOS ONE standards.**

**In reference to the above reviewer’s comments, the authors believe that the image quality was affected by being converted to the compiled submission PDF. This is supported by the following statement on PLOS ONE’s submission guidelines page (**[**https://journals.plos.org/plosone/s/submission-guidelines**](https://journals.plos.org/plosone/s/submission-guidelines)**) “The compiled submission PDF includes low-resolution preview images of the figures after the reference list. The function of these previews is to allow you to download the entire submission as quickly as possible. Click the link at the top of each preview page to download a high-resolution version of each figure. Links to download Supporting Information files are also available after the reference list.”**

19. Reviewer #2: Existing REFERENCES need to be updated.

**The authors thank the reviewer for indicating that we needed to update our references. To accomplish this, all websites provided were checked to make sure they still worked, and were updated if they did not, and all “cited …” statements were updated to 6 Sep 2020 to reflect this. We used the citation manager Mendeley with the PLOS ONE style they had on file. One citation was removed since it was the preprint for QIIME2 not the published Nature Biotechnology article.**

20. Reviewer #2: The ecological significance of the obtained results need to be discussed particular with respect to functional differences.

**The authors agree that understanding the functional differences between prokaryotic communities is important but, since this is a 16S study, that is outside the scope of our data. We did not feel comfortable making conclusions regarding functional differences between samples and sample types unless the trait in question can be attributed to all or most of the members of a bacterial group. We covered this in Lines 497-504 where we discussed the major orders and their possible contributions to the nutrient cycling in the sediment. To address this limitation, we expanded our conclusions section to stress the importance of future shotgun sequencing studies Lines 563-566.**

Notes from email:

21. Please ensure that your manuscript meets PLOS ONE's style requirements, including those for file naming.

**Thank you for reminding us to double-check our formatting, the authors are sorry that they missed some style requirements and the Title, Authors, and Affiliations page was edited to reflect these requirements. Commas were switched to after the author labels (Line 7) and the current address for Nicholas J. Dickens was removed (Line 13) to reflect that his contributions to this work only occurred during his time at Harbor Branch Oceanographic Institute. Based upon careful review of the main body style requirements, no edits were made since the document already reflected those requirements. All file names were changed to directly match what was stated in email, guidance from website, and using PACE.**

22. In your Methods section, please provide additional information regarding the permits you obtained for the work. Please ensure you have included the full name of the authority that approved the field site access and, if no permits were required, a brief statement explaining why.

**The authors appreciate you pointing out this missing statement. No permits were necessary and a statement reflecting this is included in lines 129-130.**

23. Thank you for stating the following in the Financial Disclosure section: "This research was supported by the following organizations and agencies: the Harbor Branch Oceanographic Institute Foundation (https://hboifoundation.org/) (DJB), the Everglades Foundation (https://evergladesfoundation.org/) (DJB), and the Save Our Seas License Plate (https://myfloridaspecialtyplate.com/save-our-seas.html) (PJM).The funders had no role in study design, data collection and analysis, decision to publish, or preparation of the manuscript." We note that you received funding from a commercial source: MyFloridaSpecialtyPlate Please provide an amended Competing Interests Statement that explicitly states this commercial funder, along with any other [relevant declarations](about:blank) relating to employment, consultancy, patents, products in development, marketed products, etc.

Within this Competing Interests Statement, please confirm that this does not alter your adherence to all PLOS ONE policies on sharing data and materials by including the following statement: "This does not alter our adherence to PLOS ONE policies on sharing data and materials.” (as detailed online in our guide for authors [http://journals.plos.org/plosone/s/competing-interests](about:blank)).  If there are restrictions on sharing of data and/or materials, please state these. Please note that we cannot proceed with consideration of your article until this information has been declared.

Please include your amended Competing Interests Statement within your cover letter. We will change the online submission form on your behalf.

Please know it is PLOS ONE policy for corresponding authors to declare, on behalf of all authors, all potential competing interests for the purposes of transparency. PLOS defines a competing interest as anything that interferes with, or could reasonably be perceived as interfering with, the full and objective presentation, peer review, editorial decision-making, or publication of research or non-research articles submitted to one of the journals. Competing interests can be financial or non-financial, professional, or personal. Competing interests can arise in relationship to an organization or another person. Please follow this link to our website for more details on competing interests: [http://journals.plos.org/plosone/s/competing-interests](about:blank)

**Thank you for informing us about this discrepancy, the Competing Interests Statement (below) has been amended to address the above issue.**

**The authors have declared that no competing interests exist. In specific relation to the commercial funder MyFloridaSpecialtyPlate, none of the authors have any of the following financial competing interests to declare: ownership of stocks or shares, paid employment or consultancy, board membership, patent applications, or marketed products. None of the funders involved in this research have any policies nor do any of the authors have an involvement with these funders which would alter our adherence to PLOS ONE policies on sharing data and materials.**

24. Thank you for stating the following in the Competing Interests section:"The authors have declared that no competing interests exist." We note that one or more of the authors are employed by a commercial company: Amazon.com, Inc..

4.1. Please provide an amended Funding Statement declaring this commercial affiliation, as well as a statement regarding the Role of Funders in your study. If the funding organization did not play a role in the study design, data collection and analysis, decision to publish, or preparation of the manuscript and only provided financial support in the form of authors' salaries and/or research materials, please review your statements relating to the author contributions, and ensure you have specifically and accurately indicated the role(s) that these authors had in your study. You can update author roles in the Author Contributions section of the online submission form.

Please also include the following statement within your amended Funding Statement.“The funder provided support in the form of salaries for authors [insert relevant initials], but did not have any additional role in the study design, data collection and analysis, decision to publish, or preparation of the manuscript. The specific roles of these authors are articulated in the ‘author contributions’ section.”

If your commercial affiliation did play a role in your study, please state and explain this role within your updated Funding Statement.

4.2. Please also provide an updated Competing Interests Statement declaring this commercial affiliation along with any other relevant declarations relating to employment, consultancy, patents, products in development, or marketed products, etc.

Within your Competing Interests Statement, please confirm that this commercial affiliation does not alter your adherence to all PLOS ONE policies on sharing data and materials by including the following statement: "This does not alter our adherence to  PLOS ONE policies on sharing data and materials.” (as detailed online in our guide for authors [http://journals.plos.org/plosone/s/competing-interests](about:blank)).  If this adherence statement is not accurate and  there are restrictions on sharing of data and/or materials, please state these. Please note that we cannot proceed with consideration of your article until this information has been declared.

Please include both an updated Funding Statement and Competing Interests Statement in your cover letter. We will change the online submission form on your behalf.

Please know it is PLOS ONE policy for corresponding authors to declare, on behalf of all authors, all potential competing interests for the purposes of transparency. PLOS defines a competing interest as anything that interferes with, or could reasonably be perceived as interfering with, the full and objective presentation, peer review, editorial decision-making, or publication of research or non-research articles submitted to one of the journals. Competing interests can be financial or non-financial, professional, or personal. Competing interests can arise in relationship to an organization or another person. Please follow this link to our website for more details on competing interests: [http://journals.plos.org/plosone/s/competing-interests](about:blank)

**Thank you for pointing this out, as stated above, the current address for Nicholas J. Dickens was removed to reflect that his involvement in this study only occurred while he was exclusively a member of the Harbor Branch Oceanographic Institute faculty before his present employment by Amazon.com Inc. He is still affiliate faculty at HBOI and his email address in this role is dickensn@fau.edu.**

**The authors believe that the above adjustment to the Funding and Competing Interest Statement is now no longer needed due to this change and clarification. In relation to Dr. Dickens Author Contributions Role as a funder, his startup funds provided by the Harbor Branch Oceanographic Institute Foundation was used to fund the Research Assistantship of David J. Bradshaw during Fall 2018, Spring 2019, Fall 2019, and Spring 2020. The Funding Statement (below) has been updated to reflect this.**

**This research was supported by the following organizations and agencies: the Harbor Branch Oceanographic Institute Foundation (https://hboifoundation.org/) (DJB, NJD), the Everglades Foundation (https://evergladesfoundation.org/) (DJB), and the Save Our Seas License Plate (https://myfloridaspecialtyplate.com/save-our-seas.html) (PJM).The funders had no role in study design, data collection and analysis, decision to publish, or preparation of the manuscript.**

25. We note that 1 in your submission contain map images which may be copyrighted. All PLOS content is published under the Creative Commons Attribution License (CC BY 4.0), which means that the manuscript, images, and Supporting Information files will be freely available online, and any third party is permitted to access, download, copy, distribute, and use these materials in any way, even commercially, with proper attribution. For these reasons, we cannot publish previously copyrighted maps or satellite images created using proprietary data, such as Google software (Google Maps, Street View, and Earth). For more information, see our copyright guidelines: http://journals.plos.org/plosone/s/licenses-and-copyright.

We require you to either (1) present written permission from the copyright holder to publish these figures specifically under the CC BY 4.0 license, or (2) remove the figures from your submission:

**Fig 1 was recreated using a public domain source for the image (Florida Fish and Wildlife Conservation Commission), thus it is not copyrighted. The content permission statement is thus not needed, but the figure legend has been updated to state the source of the image (Line 123-124).**
